# Regulated cell death (RCD) in cancer: key pathways and targeted therapies

**DOI:** 10.1038/s41392-022-01110-y

**Published:** 2022-08-13

**Authors:** Fu Peng, Minru Liao, Rui Qin, Shiou Zhu, Cheng Peng, Leilei Fu, Yi Chen, Bo Han

**Affiliations:** 1 West China School of Pharmacy, State Key Laboratory of Biotherapy and Cancer Center, Department of Gastrointestinal Surgery, West China Hospital, Sichuan University, Chengdu, 610041 China; 2 State Key Laboratory of Southwestern Chinese Medicine Resources, Hospital of Chengdu University of Traditional Chinese Medicine, School of Pharmacy, Chengdu University of Traditional Chinese Medicine, Chengdu, 611137 China; 3 Sichuan Engineering Research Center for Biomimetic Synthesis of Natural Drugs, School of Life Science and Engineering, Southwest Jiaotong University, Chengdu, 610031 China

**Keywords:** Drug discovery, Medicinal chemistry

## Abstract

Regulated cell death (RCD), also well-known as programmed cell death (PCD), refers to the form of cell death that can be regulated by a variety of biomacromolecules, which is distinctive from accidental cell death (ACD). Accumulating evidence has revealed that RCD subroutines are the key features of tumorigenesis, which may ultimately lead to the establishment of different potential therapeutic strategies. Hitherto, targeting the subroutines of RCD with pharmacological small-molecule compounds has been emerging as a promising therapeutic avenue, which has rapidly progressed in many types of human cancers. Thus, in this review, we focus on summarizing not only the key apoptotic and autophagy-dependent cell death signaling pathways, but the crucial pathways of other RCD subroutines, including necroptosis, pyroptosis, ferroptosis, parthanatos, entosis, NETosis and lysosome-dependent cell death (LCD) in cancer. Moreover, we further discuss the current situation of several small-molecule compounds targeting the different RCD subroutines to improve cancer treatment, such as single-target, dual or multiple-target small-molecule compounds, drug combinations, and some new emerging therapeutic strategies that would together shed new light on future directions to attack cancer cell vulnerabilities with small-molecule drugs targeting RCD for therapeutic purposes.

## Introduction

The biennial report 2020–2021 issued by the international agency for research on cancer (IARC) of the World Health Organization points out that the reality of high incidence of cancer and the rising trend also make cancer one of the main reasons that threaten human life. Cancer has become one of the most common diseases in China,^1^ which also has unique epidemiological characteristics and patient types. For example, the epidermal growth factor receptor (EGFR) mutation rate of lung adenocarcinoma patients in China is 61%, while that in the United States is only 11%.^2^ Cancer is a heterogeneous disease characterized by cell death disorder. In the face of the high incidence of cancer and the rising trend, it is urgent to clarify its deep pathogenesis and carry out the targeted treatment.

Cell death can be classified according to the morphological criteria, cellular context and triggering stimulus. In 2018, hundreds of scientists in the field of cell death jointly published an article in the journal Cell Death & differentiation, entitled “molecular mechanisms of cell death: recommendations of the Nomenclature Committee on cell death 2018”.^3^ Scientists divided the types of cell death into regulated cell death (RCD) and accidental cell death (ACD).^3^ ACD is an uncontrolled process of cell death, which is triggered by accidental injury stimuli. These injury stimuli exceed the adjustable ability of cells, resulting in cell death. RCD refers to the autonomous and orderly death of cells controlled by genes in order to maintain the stability of the internal environment. Its induction and execution are mainly regulated by the formation of signal amplification complexes that play an evolutionarily important role in development and immune response.^4^ RCD, which occurs under physiological conditions, is also known as programmed cell death (PCD).^3^ Currently known RCD types mainly include: autophagy-dependent cell death, apoptosis, necroptosis, pyroptosis, ferroptosis, parthanatos, entosis, NETosis, lysosome-dependent cell death (LCD), alkaliptosis, and oxeiptosis. Mammalian cells exposed to unrecoverable disturbances in the intracellular or extracellular microenvironment can activate one of many signal transduction cascades and eventually lead to their death. Each of these RCD patterns is initiated and transmitted by molecular mechanisms that show a considerable degree of interconnection. In addition, each type of RCD can show the full spectrum of morphological characteristics from complete necrosis to complete apoptosis, as well as the immunomodulatory characteristics from anti-inflammatory and tolerance to promoting inflammation and immunogenicity.^3,5^

Different lethal subroutines during RCD can affect cancer progression and response to treatment. In the early stage of onset, cancer cells may have the characteristics of anti-cancer treatment because of the mutation that destroys the RCD pathway, and avoiding RCD is one of the important signs of cancer. The application of RCD signal to a specific cancer type or multiple target drugs can be avoided by single or combined application of RCD signal. Based on the current research results, paying attention to the crosstalk between different RCD pathways may be a new direction of cancer treatment in the future. This manuscript will briefly describe the characteristics of the regulatory cell death mechanism and its application in tumor treatment, in order to provide new targets and new ideas for tumor treatment.

## Crucial signaling pathways of RCD subroutines in cancer

Generally, apoptosis and autophagy-dependent cell death are considered as crucial subroutines of RCD, which could induce degradation of organelles or cell death under the cellular stress and play a vital role in targeted therapy and regulation of cancer cell death.^6^ Apoptosis has been recognized as a critical intracellular process that maintains organism homeostasis and controls cell population. Several morphological characteristics of apoptosis include cell shrinkage, chromatin condensation, membrane blebbing, deoxyribonucleic acid (DNA) fragmentation, and apoptotic body formation.^7,8^ Apoptosis mainly occurs in two canonical pathways: the extrinsic pathway, stimulated by the activation of death receptors, and the intrinsic pathway, mediated by mitochondria. Binding of death ligands, namely, tumor necrosis factor α (TNFα), Fas ligand (FasL), and TNF-related apoptosis-inducing ligand (TRAIL), to the homologous death domain of its target cell surface receptors, i.e., TNF receptor 1(TNFR1), Fas, and death receptor (DR) 4/5, respectively, triggers the activation of the extrinsic apoptosis pathway, activating caspase-8 and then initiating the terminal phase or execution phase of apoptosis^9^ The intrinsic pathway is initiated when irreparable damage to cellular components occurs and is commonly modulated by B-cell lymphoma 2 (Bcl-2) family proteins. These proteins regulate the release of cytochrome c (Cyt-C) and second mitochondria-derived activator of caspases/direct IAP-binding protein with low pI (SMAC/DIABLO). Cyt-C interacts with apoptotic protease activating factor 1 (Apaf-1) proteins, which then activates caspase 9 to induce apoptosis in cancer cells.^10,11^

Autophagy, a phagocytic biological process, can disintegrate damaging proteins or organelles through lysosomal fusion and is essential for maintaining cell function and homeostasis.^12^ Autophagy has been proved to exert the dual functions in tumor progression, and it could promote or inhibit cancer development according to tumor subtype and mutation status.^13^ In the precancerous stage, the inhibition of autophagy will lead to the accumulation of reactive oxygen species (ROS), and genomic dysfunction, which collectively results in the endoplasmic reticulum (ER) pressure increased and DNA damaged, thus promoting the formation of tumors. However, when stimulated by starvation or oxidative stress, autophagy can provide energy and nutrients to tumors, which can elicit the survival of cancer cells.^14,15^ The autophagic process is controlled by autophagy-related genes. Unc-51-like kinase 1 (ULK1), Beclin-1, light chain 3 (LC3), p62, forkhead box O (FoxO), and other autophagy-related genes are involved in the regulation of autophagy, among which ULK1 acts as a promoter of autophagy and regulates the initiating function of autophagy.^16^ Autophagy-associated signaling pathways, including phosphatidylinositol 3 kinase complex 1 (PI3KC1)- protein kinase B (Akt)-mammalian target of rapamycin complex 1 (mTORC1), Ras-Raf-mitogen activated protein kinases (MAPKs) and nuclear factor kappa-B (NF-κB) pathways, also play a vital role in combating tumor progression and metastasis.

Apoptosis and autophagy are the central mechanisms that maintain cellular homeostasis and regulate cell fate. Meanwhile, there is a certain interaction between apoptosis and autophagy, which can promote cell death through an independent or complementary relationship.^17^ The targeted regulation of apoptosis and autophagy by small-molecule compounds has fully demonstrated its therapeutic potential in cancer agent development.^18^ For example, Ampelopsin (Amp) has been shown to trigger apoptosis and autophagy-dependent cell death by promoting ROS generation and the activation of c-Jun N-terminal kinase (JNK) in glioma cells.^19^ Galectin-1 is a member of the galactose lectin family with multiple biological activities. It is highly expressed in numerous tumors and regulates the proliferation, migration, and growth of tumor cells.^20^ Shikonin could be a promising anti-colorectal agent to attenuate tumor growth. It was shown that shikonin could target galectin-1 and activate the JNK signaling pathway to induce apoptosis and autophagy in colorectal carcinoma (CRC) cells.^21^ Interestingly, dihydroartemisinin (DHA), as an active metabolite, regulated apoptosis, and autophagic cell death by blocking the Wnt/β-catenin signaling pathway and stimulating the p38/MAPK pathway in multiple myeloma (MM).^22^ F1012-2, an active component isolated from Eupatorium lindleyanum DC., effectively inhibited cell growth by triggering apoptosis via intrinsic and extrinsic pathways in triple negative breast cancer (TNBC) cells. Besides, the induced apoptosis could be increased by inhibiting autophagy.^23^

Necroptosis is a regulatory cell death mode driven by receptor-interacting serine/threonine kinase protein (RIPK) 1 through its kinase function to form complex IIB, which leads to cell necroptosis. It has the morphological characteristics of necroptosis cells and a signal mechanism similar to apoptotic cells. Morphologically, it is characterized by cell membrane perforation, increased intracellular osmotic pressure, resulting in cell rounding and swelling, organelle swelling, mitochondrial dysfunction, loss of mitochondrial membrane potential, loss of nuclear chromatin, and explosive rupture of the plasma membrane. The cancer cell contents released after cell rupture exacerbate the peripheral inflammatory response. The difference from necrosis is that necroptosis strictly follows the cancer intracellular signal regulation and has the characteristics of active energy consumption. After TNF-α binds to TNFR1 on the plasma membrane, downstream protein molecules are recruited to form complex I. The protein of RIPK1 is transformed into the cytoplasmic receptor of RIPK1.^24^ Depending on the stimuli or the cancer cellular microenvironment, complex I activates different signaling pathways downstream through the regulation of RIPK1, resulting in two death modes, apoptosis, and necroptosis. Polyubiquitination of the Lys63 domain of RIPK1 promotes the recruitment of Ikappa B kinase (IKK) and transforming growth factor kinase (TAK) into a complex, and both TAK and IKKα/IKKβ complexes activate NF-κB and promote cancer cell survival (NF-κB dependent).^25,26^ In addition, IKKα/IKKβ, TANK-binding kinase 1 (TBK1), and IKKε also inactivate the phosphorylation of RIPK1 and prevent its translocation into complex II, thereby preventing RIPK1-dependent cell death (Non-NF-κB dependent).^27,28^ Intracellular death promoting protein RIPK1 promotes the recruitment of pro-caspase-8 and produces activated caspase-8, which leads to apoptosis.^29,30^ If caspase-8 is inhibited or not expressed in cancer cells, RIP3 is recruited to form RIPK1-rip3 complex, which causes ripk3 phosphorylation to recruit executive protein mixed lineage kinase domain-like pseudokinase (MLKL), form necrotic body (also known as complex IIB), and trigger necroptosis in cancer.^31,32^ Activated RIPK1 acts as an intermediate bridging complex I and complex II during TNF-α-induced apoptosis.^33^ Linking complex I and complex II through the formation of RIPK1, which is also observed in partial necroptosis, a marker that can be used to determine the outcome of complex I disruption.^33^ Complex I will ultimately determine whether cancer cells survive, apoptosis or necroptosis by regulating the functional conversion of RIPK1. Apoptosis and necrosis are the two earliest and most classical ways of cell death. As an autonomous and orderly death mode of cells controlled by genetic genes, the former is not only a phenomenon that occurs in the specific growth and development stage of most cells in organisms, but also an essential process for cells in organisms to maintain normal activity and function. Different from apoptosis, necrosis is generally considered to be uncontrollable, which is a way of death defined by morphological characteristics. Excessive external inhibitory factors can directly cause necrosis, which is a passive cell death affected by the environment. However, recent studies have shown that necrosis can also be regulated by the intracellular signal transduction pathway, which cannot be mediated by caspase. Therefore, it can still play a role when the apoptotic pathway is inhibited, and its cell morphology is consistent with conventional necrosis. In 2005, regulated necrosis was first found. This includes many ways, such as secondary necrosis, self-death, iron apoptosis, pyroptosis, parp-1-dependent cell death, necrotic apoptosis, cyclophilin necrosis, and so on. It has the morphological characteristics of necrosis, such as nuclear fragmentation, swelling of cells and organelles, rupture of the plasma membrane, and so on. Many studies have proved that necrotic apoptosis or programmed necrosis plays an important role in the occurrence, development, invasion, metastasis, and drug resistance of malignant tumors. Cell resistance to necroptosis is often mediated by oncogenes, suggesting that escape from necroptosis may be a potential tumor marker similar to escape from apoptosis. Tumor therapy based on necroptosis is a new strategy of cancer therapy, but its feasibility is still controversial. Supporters believe that because necroptosis and apoptosis play a role through different signal pathways, inducing necroptosis of tumor cells has the potential to be used as an alternative therapy for anti-apoptotic malignant tumors. According to the current research, this hypothesis has been preliminarily verified. However, skeptics believe that congenital or acquired defects in the necrosis mechanisms have been observed in many cancer cells. Whether the used necrosis inducers can selectively kill cancer cells without interfering with normal cell activities and whether they will lead to de inflammation in organisms need further research. Apoptosis is a form of programmed cell death; its autonomous cell lysis will not cause inflammation and actively participate in the process of life and death balance of tumor cells.

Pyroptosis is a form of programmed cell death associated with an inflammatory response. Gasdermins family is the primary executor of pyroptosis, including gasdermin-a (GSDMA), gasdermin-b (GSDMB), gasdermin-c (GSDMC), gasdermin-d (GSDMD), gasdermin-e (GSDME, also known as DNFA5), DFNB59 and other proteins. The characteristics of pyroptosis in cancer are mainly the cleavage and polymerization of gasdermins family proteins, the cleavage of N-terminal and C-terminal junction domains of gasdermins, and the release of activated N-terminal regions. The N-terminal binds to membrane lipids, phosphatidylinositol, and cardiolipin, and forms pore in the cell membrane, resulting in cell osmotic swelling, plasma membrane rupture, and death.^34,35^ Gasdermins family proteins form 10 to 20 nm holes in the cell membrane, and the cell contents are slowly released through the membrane holes (which can trigger amplified inflammatory reactions). The cells gradually flatten and produce 1–5 μm apoptotic body-like protrusions (focal dead bodies). The cells gradually swell to the rupture of plasma membrane, with nuclear concentration and chromatin DNA breakage.^36^ The pyroptosis pathway can be divided into classical and non-classical pyroptosis pathways in cancer. The activation of the classical pyroptosis pathway is initiated by pathogen-associated molecular patterns (PAMPs) or sterile molecular patterns (DAMPs).^37,38^ They are recognized by cytoplasmic pattern recognition receptors (PRRS). After recognizing specific stimuli, nod-like receptors (NLRs) or melanoma deficiency factor 2-like receptors (ALRs) initiate assembly to form inflammatory bodies and process to form activated caspase-1. Caspase-1 cleaves GSDMD, and the N-terminal of GSDMD is localized and aggregated into pores on the cell membrane. In addition, caspase-1 cleaves pro-IL-1β and pro-IL-18 to form mature IL-1β and IL-18, and the intracellular contents are secreted outside the membrane through the membrane pore. The nonclassical pyrolytic pathway depends on the activation of caspase-4/caspase-5/caspase-11. After the cytoplasm is stimulated by lipopolysaccharide (LPS), caspase-4/caspase-5/caspase-11 (the human counterpart of mouse caspase-11 caspase-4/caspase-5) can directly bind to the conserved structure of LPS, lipoprotein A, causing oligomerization, leading to activation, further cutting GSDMD, causing the N-terminal of GSDMD to be cleaved and localized to the cell membrane to form membrane pores.^39^ Compared with apoptosis, pyroptosis occurs faster and more violently, accompanied by the release of many pro-inflammatory factors. Inflammatory corpuscles and GSDM family proteins are the key substrates causing cell scorch. At present, in the research on digestive system tumors, hematological system tumors, respiratory system tumors, and reproductive system tumors, it has been found that the above two are involved in inhibiting the growth of tumor cells and promoting tumor cell death. In addition, some research results also suggest that cell scorch can also promote the growth of tumors in different kinds of tumor cells. This shows that cell pyroptosis plays a dual role in promoting and inhibiting tumors. It is also necessary to further study the relationship between cell pyroptosis and tumor occurrence and progression.

Ferroptosis is a new form of oxidative and non-apoptotic programmed cell death found in recent years. It is different from other cell death modes such as apoptosis, autophagy, and necrosis in morphology, genetics, and molecular biology.^40^ The main characteristics of ferroptosis are cell death induced by iron-dependent lipid peroxide injury in mitochondria, accompanying with the deficiency of activity of the lipid repair enzyme glutathione peroxidase 4 (GPX4). Its biochemical characteristics are mainly manifested in the inhibition of cystine/glutamate transporter system, the increase of nicotinamide adenine dinucleotide phosphate (NADPH) oxidation, and the accumulation of ROS caused by iron overload, and the increase of lipid peroxidation products. Ferroptosis has unique morphological and bioenergetic characteristics. Its morphological characteristics are that the cell membrane is not broken, the plasma membrane blisters, mitochondria shrink, the density of the mitochondrial membrane increases, the mitochondrial cristae decreases or disappears, and the nuclear size is normal, but the chromatin does not condense. The level of intracellular lipid peroxidation is finely regulated: on the one hand, the highly expressed polyunsaturated fatty acids on the cell membrane are very vulnerable to the attack of lipid ROS induced by divalent iron or oxidized by lipoxygenase, leading to the cascade reaction of lipid peroxidation and the accumulation of a large number of lipid peroxides; On the other hand, GPX4 of the antioxidant system will reduce the lipid peroxide to the corresponding lipid alcohol, so as to reduce the burden of lipid peroxidation and protect the cell membrane from damage. Only when this regulation system is out of balance, and the accumulation of lipid peroxide reaches a lethal amount will ferroptosis occur. The occurrence of ferroptosis is closely related to the accumulation of iron in cells, the production of free radicals, the supply of fatty acids, and lipid peroxidation. Extracellular Fe^3+^ binds to transferrin (TF) and is transported into cells through transferrin receptor 1 (TfR1) and reduced to Fe^2+^.^41^ After that, it was stored in the intracellular labile iron pool (LIP) with the help of intracellular divalent metal transporter 1 (DMT1) and zinc transporter 8/14 (ZIP8/14).^42^ Fe^2+^ can transfer electrons through Fenton reaction with peroxide to produce free radicals with oxidation ability. When intracellular iron is overloaded, a large number of free radicals can react with polyunsaturated fatty acid (PUFA) of cell membrane phospholipids under the catalysis of ester oxygenase and iron to produce a large number of lipid peroxides, resulting in cell death.^8^ At the same time, the intracellular antioxidant stress system mainly relies on GPX4 to remove excess lipid peroxides. Cystine/glutamate antiport, also known as system x_c_^−^ is responsible for transporting glutamate out of cells and the same amount of cystine into cells. When it is blocked by system x_c_^−^ inhibitors such as erastin, it prevents cystine from entering cells, resulting in the reduction of the content of cysteine necessary for the synthesis of glutathione (GSH) and the obstruction of the synthesis of GSH. GSH is involved in the process of GPX4 hydrolyzing lipid peroxide. The inhibition of GSH synthesis or the inactivation of GPX4 can make the excess lipid peroxide in cells unable to be removed, resulting in cell oxidative damage and inducing ferroptosis.^43^ Therefore, inhibiting system x_c_^−^, consuming GSH, and inactivating GPX4 are the key nodes to induce ferroptosis. Therefore, many ferroptosis inducers have been developed. Ferroptosis, non-apoptotic regulatory cell death found in recent years, is a hot issue in biological and medical research. However, at present, the compounds that induce ferroptosis are only effective for some tumor cells, and different kinds of cancer seem to have different sensitivity to ferroptosis. Loading ferroptosis inducer, reactant of ferroptosis process or traditional Chinese medicine preparation through nanotechnology to target the tumor site and make the drug concentration gather at the tumor site, which may bring new options for cancer treatment based on ferroptosis.

Poly (ADP-ribose) polymerase-1 (PARP-1)-dependent cell death (parthanatos) is a new type of regulatory cell death. It was named parthanatos in 2008.^44^ “Par” stands for par (poly ADP ribose), and the suffix “Thanatos” comes from ancient Greek mythology, which means “death”. The process of parthanatos is different from apoptosis and other regulatory necrosis. It is mainly manifested in: when parthanatos occurs, PARP-1 is abnormally activated and produces a large amount of par; When the mitochondrial membrane is depolarized, the levels of ATP and NADPH decrease, and the apoptosis-inducing factor (AIF) enters the nucleus from mitochondria; Although caspase is activated in the late stage of parthanatos, caspase inhibitor can not inhibit the regulatory cell necrosis, but PARP-1 inhibitor or PARP-1 gene knockout can prevent its occurrence;^45^ AIF is transferred to the nucleus, where chromatin condenses and produces a large number of DNA fragments ranging from 15 KB to 50 KB. Parthanatos widely occurs in many pathological processes such as inflammatory injury, ROS-induced injury and tumor. The occurrence process will lead to abnormal activation of PARP-1 and produce a large number of ADP ribose polymers (PAR) connected by glycosidic bonds. Par itself has toxic effects on cells. Therefore, the signal transduction of par polymer to mitochondria and the transfer of AIF from mitochondria to the nucleus is the crucial way to causing parthanatos. Parthanatos is a new type of programmed death different from apoptosis and necrosis. Its main feature is that caspase is not involved in this process. PARP-1 inhibitor or PARP-1 gene deletion can completely block the occurrence of parthanatos, while caspase inhibitors can not inhibit the occurrence of parthanatos. In addition to brain injury diseases, many factors such as ROS, ultraviolet irradiation and alkylating agents can also cause DNA breakage, which can lead to the over activation of PARP-1 and the occurrence of parthanatos. The overactivation of PARP-1, par accumulation, and AIF nuclear displacement are the main signs of parthanatos. Parthanatos has some of the same characteristics as necroptosis, apoptosis, and autophagy, but the molecular mechanism is different. Compared with apoptosis, parthanatos could not form small fragments of DNA fragments and apoptotic bodies; Compared with cell necrosis, parthanatos could not cause organelle swelling; Compared with autophagy, parthanatos did not form autophagic vesicles and lysosomal degradation; Compared with necroptosis, parthanatos did not cause swelling of cell membrane and organelles, cell lysis, and RIPK1 activation. Different from apoptosis and necrosis, they are the process of chromatin degradation and particle release into extracellular space.

**Fig. 1:**
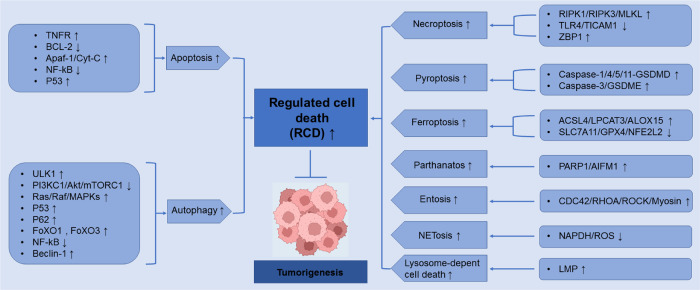
Crucial signaling pathways of RCD subroutines in cancer

In this review, we not only focus on apoptosis and autophagy, two pivotal pathways that regulate cell death, but also involve other RCD subroutines such as necroptosis, pyroptosis, ferroptosis, parthanatos, entosis, NETosis and Lysosome-dependent cell death (LCD). Therefore, we summarize some representative small-molecule compounds which can target these subroutines, control cancer cells’ survival, and thus improve the efficacy of cancer therapy (Fig. 1).

### Apoptotic signaling pathways in cancer

**Fig. 2:**
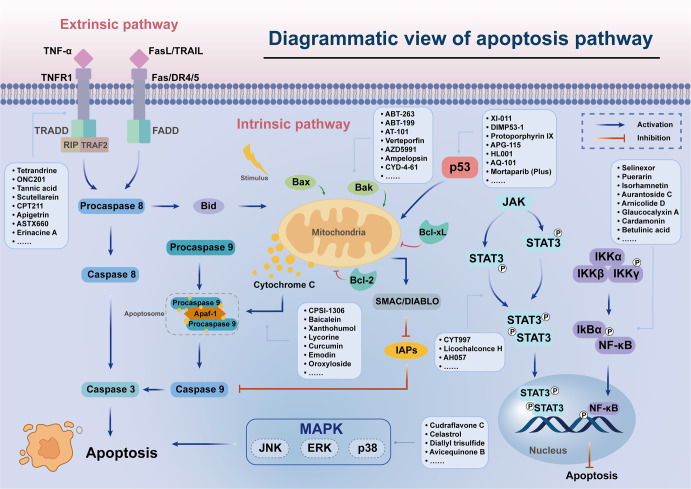
Small-molecule compounds targeting apoptosis-related pathways in cancer. There are two core apoptosis pathways, intrinsic and extrinsic. The extrinsic pathway is initiated by multiple death receptors, such as TNFR1, Fas, and DR4/5. The intrinsic pathway is mediated by Bcl-2 family proteins. Activation of either pathway ultimately triggers a cascade of caspases, thus inducing caspase-dependent nucleosome fragmentation leading to cell death. In addition, NF-κB, JAK-STAT3, and MAPKs signaling pathways play an essential role in regulating cell apoptosis

Apoptosis refers to the spontaneous and orderly death of cells controlled by multiple genes in order to maintain the stability of the internal environment. Inhibition or resistance of cell death often leads to the occurrence of tumors.^46,47^ Therefore, the regulation of the apoptosis signaling pathway is one of the crucial methods to improve cancer treatment.^48^ Then, we focus on summarizing some representative small-molecule compounds that ultimately induce cancer cell death through the regulation of some crucial apoptotic signaling pathways and targets, such as TNF-related ligands and their receptors, Bcl-2 family, Apaf-1 and Cyt-C, NF-κB pathway, p53, etc. (Fig. 2).

#### Targeting TNF-related ligands and their receptors

Tumor necrosis factor (TNF) is a regulatory cytokine, as well as an essential signal transduction protein. The death receptor-mediated apoptosis pathway could be triggered by binding of TNF-related ligands such as FasL, TNFα, and TRAIL to their corresponding receptors Fas, TNFR1, and DR4/5, respectively.^11,49^ This interaction subsequently activates the recruitment of death domain-containing adaptor proteins, like Fas-associated protein with death domain (FADD) and TNFR1-associated death domain (TRADD), which could bridge the death effector domain (DED) to pro-caspase 8, forming the death-inducing signaling complex (DISC). DISC promotes the activation of pro-caspase-8 by cleavage and then activates other caspase proteins, leading to apoptosis execution.^50,51^ In this review, we have summarized some small-molecule compounds that promoted cell apoptosis and suppressed the growth of cancer cells by targeting TNF-related receptors and their ligands.

TRAIL, also called TNF superfamily 10, is a pleiotropic cytokine from the TNF superfamily. TRAIL has been shown to selectively induce apoptosis in various tumor cells without affecting normal cells.^52^ In this report, Shishodia et al. found that tetrandrine (TET) could sensitize resistant and mildly sensitive prostate cancer cells to TRAIL-induced apoptosis, and these effects were regulated by upregulating the messenger ribonucleic acid (mRNA) expression of DR4/DR5.^53^ ONC201, a TRAIL-inducing compound expressed the potential anti-cancer activity in numerous cancer cell lines. ONC201 bound to dopamine receptors DRD2 and DRD3, as well as mitochondrial caseinolytic protease P (ClpP), resulting in activation of activating transcription factor 4 (ATF4), which leads to DR5 upregulation and cell death dependent on C/EBP homologous proteins (CHOP).^54,55^ ABBV-621 is a TRAIL agonist that could enhance caspase-8 aggregation and the formation of death signal complexes independent of FcγR-mediated cross-linking. The research showed that ABBV-621 could induce cell death in ~36% of solid cancer cell lines in vitro at subnanomolar concentrations. ABBV-621 could overcome the resistance in ABBV-621 therapy when combined with chemotherapeutic agents or selective inhibitors of B-cell lymphoma-extra large (Bcl-xL). In a word, ABBV-621 shows therapeutic antitumor efficacy in phase 1 clinical trial (NCT03082209).^56^ Tannic acid (TA), as a natural polyphenol compound, has a more effective anticancer activity. TA was shown to arrest sub-G1 cell cycle arrest and induce apoptosis through enhancing the generation of mitochondrial (mROS) and further stimulating the TRAIL-induced extrinsic apoptosis pathway in NCCIT cells.^57^

Traditional Chinese Medicine (TCM) has been considered a new source of candidate small-molecule agents due to natural products possessing diverse bioactivities. Scutellarein (SCU), a flavone compound isolated from the perennial herb *Scutellaria baicalensis*, was reported to present antitumorigenic effects by promoting Hep3B cell apoptosis. SCU increased the expression level of Fas and FasL to activate caspase 8 and caspase 3, eventually causing Fas-mediated extrinsic apoptosis.^58^ CPT211, a novel camptothecin derivative, had been reported to suppress the proliferation and induce apoptosis of MDA-MB-231 cells effectively by activating Fas/FADD/caspase-8 signaling.^59^ Moreover, Apigetrin, a flavonoid glycoside compound, was shown to exert an antiproliferation effect on AGS cells. It could upregulate extrinsic apoptosis proteins expression like Fas, FasL, and DR4, as well as induce autophagy by increasing the beclin-1 and p62 proteins.^60^ Imipramine was a tricyclic antidepressant that triggered extrinsic apoptosis by upregulating FasL and y activating caspase 8/3 in glioblastoma cells. In an in vivo experiment, imipramine could also effectively attenuate tumor growth by suppressing the extracellular signal-regulated kinase (ERK)/NF-κB pathway activation.^61^ Gentian violet (GV) has an inhibitory effect on the survival and growth of cutaneous T-cell lymphoma (CTCL) tumors. GV could kill the CTCL cells by upregulating DR4/5, TRAIL, and FasL expression, triggering the extrinsic apoptosis pathway.^62^

Epigenetic inheritance is critical for gene expression and stability, and its disruption is thought to play an important role in the development of many tumor types.^63^ Histone deacetylation is an essential epigenetic event involved in the development and progression of cancer by regulating DNA expression.^64^ Compound 3 was a potent histone deacetylase (HDAC) inhibitor that could inhibit lung cancer cell growth and is an effective compound for the epigenetic remodeling activity of A549 cells. Compound 3 induced cancer cell apoptosis through both extrinsic and intrinsic pathways. It facilitated the expression of procaspase 8, FasL/Fas, and TNF-α to activate the extrinsic pathway and upregulated Bax, downregulated Bcl-2, thereby releasing Cyt-C to activate the intrinsic pathway.^65^ In non-small cell lung cancer (NSCLC) cell lines, after pemetrexed treatment, the expression of TNFRSF10B and a vesicular trafficking regulator protein, yip domain family 2 (YIPF2), was increased. YIPF2 facilitated chemotherapeutic drug-mediated apoptosis by promoting TNFRSF10B cell membrane circulation in NSCLC.^66^ ASTX660 as a cIAP1/2 and X-linked inhibitor of apoptosis protein (XIAP) antagonist could sensitize murine oral cancer (MOC1) cells to TNF-α and induce apoptosis of TNFR superfamily downstream cells. Besides, ASTX660 combined with cisplatin and PD-1 blockade could delay tumor growth.^67^

Recently, with the development of metal complexes as anti-cancer agents, their mechanism of action has gained more attention. The research has shown that ruthenium (Ru) complex 2c could target death receptors like DR5 and Fas to trigger the extrinsic apoptosis pathway. In addition, complex 2c entered the nucleus and interacted with DNA to activate p53 protein, ultimately promoting apoptosis. Compared with the chemotherapeutic drug cisplatin, complex 2 possessed lower toxicity in vivo and had considerable antitumor activity.^68^ Furthermore, the Mn^III^ complex was reported to enhance the activity of caspase 8 and caspase 9, upregulate the Bax/Bcl-2 ratio expression, and promote the binding of TNF-α to its receptor, indicating a simultaneous activation of both intrinsic and extrinsic apoptotic pathways in MDA-MB-231 cells.^69^ Apart from the compounds mentioned above, other small-molecule compounds that induce apoptosis by targeting TNFR-related proteins are also summarized in Table 1.^70–77^

**Table 1:** Compounds targeting TNF-related ligands and their receptors in cancer

Compound name and structure	Target	Mechanism in RCD	Cancer cell line (activity)	Tumor type	Clinical trial identifier	Ref.
Tetrandrine	TRAIL/DR4/5↑	Induce apoptosis	LNCaP (IC_50_ = 5–10 μM)	Prostate cancer		^53^
ONC201	TRAIL/DR5↑	Induce apoptosis		Endometrial carcinoma, breast cancer	NCT03394027 (phase 2)	^54^
Tannic acid	TRAIL, DR4/5, TRADD↑	Induce apoptosis	NCCIT (IC_50_ = 50 μM)	Embryonic carcinoma		^57^
Scutellarein	Fas/FasL↑	Induce apoptosis	Hep3B cells	Hepatocellular carcinoma		^58^
CPT211	Fas/FADD/caspase-8↑	Induce apoptosis	MDA-MB-231 (IC_50_ = 478.4 nM)	Triple negative breast cancer		^59^
Apigetrin	Fas, FasL, DR4↑	Induce apoptosis	AGS (IC_50_ = 52.13 ± 2.19 μM)	Gastric cancer		^60^
Imipramine	Fas/FasL↑	Induce apoptosis	U-87 MG and GBM8401 cells	Glioblastomas	NCT04863950 (phase 2)	^61^
Gentian violet	DR4/5, TRAIL, FasL, caspase 8↑	Induce apoptosis	MyLa, HH, SZ4, Hut-78	Cutaneous T-cell lymphoma		^62^
Compound 3	FasL/FasR, TNF-α, caspase 8↑ Cyt-C↑Bax↑Bcl-2↓	Induce apoptosis	A549	Non-small cell lung cancer		^65^
Pemetrexed	TNFR↑	Induce apoptosis	H1792, H1299, and A549	Non-small cell lung cancer	NCT01769066 (phase 2/3)	^66^
ASTX660	FasL, TNF-α, TRAIL↑	Induce apoptosis	Murine oral cancer cell lines	Head and neck squamous cell carcinomas	NCT05245682 (phase 1)	^67^
Ruthenium complex 2c	Fas, DR5↑ p53↑	Induce apoptosis	A375 (IC_50_ = 16.9 ± 3.1 μM), MCF-7 (IC_50_ = 30.2 ± 4.3 μM), A549 (IC_50_ = 59.3 ± 6.1 μM)	Melanoma; breast cancer, lung cancer		^68^
Mn^III^ complex	TNF-α/TNFR↑ Bcl-2↓ Bax↑ caspase-8,9↑	Induce apoptosis	MDA-MB-231 (IC_50_ = 2.28 ± 0.38 μM)	Triple negative breast cancer		^69^
Erinacine A	TNFR1, Fas/FasL↑ Bcl-2, Bcl-xl↓	Induce apoptosis	DLD-1 cells	Colorectal cancer		^70^
C20E	TNFR1/ASK1/JNK↑	Induce apoptosis	MDA-MB-231 (IC_50_ = 40 μM)	Triple negative breast cancer		^71^
1,3-diphenyl-2-benzyl-1,3-propanedione (DPBP)	FasL↑	Induce apoptosis	B16F10 (IC_50_ = 6.25 μM)	Melanoma		^72^
Licochalcone B	DR4/5↑ Apaf-1, Bax↑	Induce apoptosis	A375 (IC_50_ = 13.7 μM), A431 (IC_50_ = 19.1 μM)	Melanoma, squamous cell carcinoma		^73^
Erinacine S	FasL, TRAIL↑ Bcl-2, Bcl-xL↓	Induce apoptosis	ASG (IC_50_ = 3−5 μM), TSGH-9201 (IC_50_ = 8−10 μM)	Gastric cancer		^74^
Cedrol	Fas/FasL/Caspase-8↑ Bax↑Bcl-2↓	Induce apoptosis	DBTRG-05MG (IC_50_ = 91.65 μM)	Glioblastoma		^75^
3β-Acetyl-nor-erythrophlamide (C5)	TNFR1↑	Induce apoptosis	Ramos and A549 Cells	Lung cancer, lymphoma		^76^
Demethylzeylasteral (T-96)	Caspase 8/3↑	Induce apoptosis	DU145 and PC3 cells	Prostate cancer		^77^

↓ decrease/inhibition, ↑ increase/activation

#### Targeting Bcl-2 family

The B-cell lymphoma-2 (Bcl-2) family is an essential regulatory factor in the mitochondria-mediated apoptosis pathway, controlling apoptosis and survival through the interaction of pro-apoptotic and anti-apoptotic molecules, and is the most extensive class of proteins in apoptosis research.^78^ Bcl-2 family proteins are divided into three groups, which are comprised of anti-apoptotic proteins (Bcl-2, Bcl-xL, Bcl-w, and Mcl-1), pro-apoptotic proteins (Bax, Bak, and Box), and Bcl-2 homology domain 3 (BH3)-only proteins (Bad, Bim, and Bid).^79,80^ Overexpression of anti-apoptotic Bcl-2 family proteins or loss of pro-apoptotic proteins are frequently observed in various human tumors.^81^ Therefore, targeting these proteins with small molecules was proving to be an attractive strategy for anticancer therapy. Meanwhile, targeting anti-apoptotic proteins could also restore the sensitivity of cancer cells to apoptotic stimulation.

Some small-molecule inhibitors targeting Bcl-2 family proteins have been developed as classic therapy for cancers. For example, ABT-263 (navitoclax) is a Bcl-2/Bcl-xL inhibitor, which could block Bcl-xL to sequester activator BH3-only molecules (BH3s) without the obvious effect on Bax. The response to ABT-263 is closed to the expression of Mcl-1 protein in small cell lung cancer (SCLC) cells.^82^ However, low expression of Mcl-1 in NSCLC cells could not imply that ABT-263 is of therapeutic significance. It was found that increased the expression of intracellular ROS could upregulate the sensitivity of NSCLC cells to a certain extent, which could be used as a new marker for diagnosis and treatment.^83^ In addition, ABT-263 was found to induce apoptosis in human oral cancer-derived cell lines via increasing the expression of C/EBP-homologous protein (CHOP) and its mRNA.^84^ A selective Bcl-2 inhibitor, ABT-199 (Venetoclax), has received FDA approval for the treatment of chronic lymphocytic leukemia (CLL) and acute myelocytic leukemia (AML). Meanwhile, numerous trials have been conducted on other malignancies.^80^ Lochmann et al. reported that ABT-199 could trigger Bim-dependent apoptosis in SCLC cell lines via the disruption of Bim and Bcl-2 complexes. Besides, ABT-199 could also inhibit tumor growth and promote tumor regression in vivo. And ABT-199 combined with doxorubicin (DOX) or dinaciclib could effectively improve the therapeutic outcome of SCLC.^85^ Another Bcl-2 inhibitor, AT-101, was used to explore its antitumor activity and the mechanism of targeting cancer stem cells (CSCs) and anti-apoptotic proteins in gastro-esophageal cancers (GEC).^86^ AT-101 could induce apoptosis of cells with Bcl-2/Mcl-1 high expression in gastric cancer tissues and then inhibit cell proliferation and growth. In vivo studies had shown that AT-101 combined with docetaxel increased antitumor activity and significantly decreased CSCs biomarkers (YAP1/SOX9). In a preliminary clinical trial, 13 patients received AT-101 in combination with chemoradiotherapy for locally advanced esophageal or gastroesophageal junction cancer. (NCT00561197) The results showed relief of clinical symptoms and improved overall survival.^86^

Yes-associated protein 1 (YAP) is an essential downstream factor in the Hippo signal cascade that regulates cell proliferation, apoptosis, and angiogenesis. YAP is overexpressed in several types of malignant tumors and is involved in the occurrence and development of tumors, which may be a potential therapeutic target for cancer therapy.^87^ Verteporfin as a photosensitizer could inhibit the proliferation of pancreatic ductal adenocarcinoma (PDAC) PANC-1 and SW1990 cells, blocking cells at the G1 phase and further inducing apoptosis. It could suppress the interaction in YAP and TEAD by downregulating the expression of cyclinD1, cyclinE1, and Bcl-2 protein.^88^ Furthermore, (E)-2-benzylidene-3-(cyclohexylamino)-2,3-dihydro-1H-inden-1-one (BCI) is a phosphatase 1/6 and MAPK inhibitor that could inhibit the viability of lung cancer cells. In NCI-H1299 cells, BCl could downregulate the level of Bcl-2 protein and upregulate the Bax protein expression, thereby promoting the release of Cyt-C and activating caspase 8 to trigger apoptosis.^89^

McL-1 is an anti-apoptotic protein in the Bcl-2 family proteins, which is essential for the survival of normal cells. It is overexpressed in various cancers, such as lung cancer, colon cancer, multiple myeloma, etc., and is closely associated with poor prognosis.^90,91^ Therefore, targeting McL-1 is a promising therapeutic strategy for cancer. Zhu et al. had developed 3,5-dimethyl-4-sulfonyl-1H-pyrrole-based compound 40 as an Mcl-1 inhibitor and found that it could trigger the apoptosis pathway in Mcl-1 dependent way. Additionally, oral compound 40 could attenuate tumor growth in MV4-11 xenograft models.^92^ Similarly, AZD5991 had shown robust antitumor activity in multiple myeloma and acute myeloid leukemia (AML) models. It could induce apoptosis through activating the Bak-dependent intrinsic apoptosis pathway to activate caspase 3 by binding to Mcl-1. Based on these experimental data, a phase 1 clinical trial (NCT03218683) was initiated to evaluate the validity of AZD5991 in hematological malignant tumors.^93^

It was worth noting that natural compounds or their synthetic derivatives have gradually become a new source for discovering antitumor drug candidates. Garciniaxanthone I (GXI) was a novel active compound isolated from the bark of *G. xanthochymus*. It could trigger HepG2 cell apoptosis via upregulating Bax protein levels and decreasing the Bcl-2, Bcl-xL, and Mcl-1 levels. Besides, GXI could inhibit cell migration by downregulating the expression of MMP-7 and MMP-9.^94^ Ampelopsin, a plant-derived natural compound, possesses various pharmacology properties, including anticancer, anti-inflammatory, antibacterial, and so far. Ampelopsin was shown to activate the apoptosis pathway by regulating Bcl-2 family proteins in K563 and HL60 leukemia cells. Furthermore, it could inhibit cell growth by suppressing the Akt and NF-κB pathways.^95^ It could be a potential agent for the treatment of leukemia. A natural flavone compound, 5,3′-dihydroxy-3,6,7,8,4′-pentamethoxyflavone (PMF), was reported to induce an intrinsic apoptotic pathway in MCF-7 cells via enhancing the expression of Bax, Cyt-C, and PARP-1, decreasing the Bcl-2 level.^96^ Some other natural products, like Tracheloside (TCS) and Deoxypodophyllotoxin (DPT), could significantly inhibit the growth of CRC cells.^97,98^

Fu et al. synthesized a series of trimethoxyphenyl-1,2,3-triazole compounds, among which triazole containing coumarin structure 19c possessed the best antitumor activity, superior to colchicine. Compound 19c was shown to trigger apoptosis via upregulating the expression of Bax and DR5, as well as downregulating the Bcl-xL and XIAP protein levels. Further, the research showed that compound 19c could bind to the colchicine site to inhibit tubulin polymerization.^99^ A recent study showed compound 8 as a novel steroidal-chalcone compound was synthesized, which used two hydrophilic amide linkages to synthesize a steroidal hybrid molecule and exerted anti-TNBC activity.^100^ It triggered apoptosis by downregulating the Bcl-2/Bax protein ratio and activating caspase-3. In addition, upregulating ROS levels could accelerate the apoptosis of MDA-MB-231 cells.^100^

Among the new anticancer drugs currently studied, metal-based drugs have become an important one. More and more metal-based complexes with high efficiency, low toxicity, and high anticancer activity have been synthesized.^101^ The Pt(IV) complexes 14 and 17 designed and synthesized by Huang et al., showed better anticancer activity in human cancer cells than the mother Pt(II) counterparts, but their antitumor activity was different due to the difference in carbon chain lengths. They could induce apoptosis of HepG-2 cells through the release of Cyt-C, downregulation of Bcl-2, upregulation of Bax, and activation of caspase 9/3.^102^ In addition to platinum-based complexes, other metal cores have been explored, such as Ir, Co, Zn, and Cu, which were considered to be new therapeutic drug candidates.^103^ For example, iridium (III) complexes possess strongly anticancer activity. [Ir(ppy)_2_(THPDP)]PF_6_ (Ir-1) was synthesized to induce cell apoptosis by activating ROS to cause mitochondrial dysfunction, which was indicated by the expression level of the Bcl-2 family and the release of Cyt-C. Besides, the experiment result showed that Ir-1 could also induce apoptosis by suppressing the PI3K/Akt/mTOR pathway.^104^ Moreover, copper complexes containing benzimidazole exhibit favorable anticancer and antimicrobial activities but with toxic side effects. Therefore, Qi et al. introduced dipeptides into Cu(II) complex to alleviate its toxicity. [Cu(Gly-*L*-leu)(HPBM)(H_2_O)]ClO_4_ had been synthesized and shown excellent stability in the buffer solutions. The group had further explored its anticancer mechanism, and the results indicated that it could regulate Bcl-2 family proteins level and activate ROS to trigger apoptosis of HeLa cells.^105^ Likewise, numerous small-molecule compounds could trigger apoptosis of cancer cells by regulating Bcl-2 family proteins^106–113^ (Table 2).

**Table 2:** Compounds targeting Bcl-2 family in cancer

Compound name and structure	Target	Mechanism in RCD	Cancer cell line (activity)	Tumor type	Clinical trial identifier	Ref.
ABT-263 (Navitoclax)	Bcl-2, Bcl-xL↓	Induce apoptosis	H2171 (EC_50_ = 0.02 μM), DMS53 (EC_50_ = 0.1482 μM), H446 (EC_50_ = 1.87 μM), H82 (EC_50_ = 5.206 μM), DMS114 (EC_50_ = 5.509 μM), H196 (EC_50_ = 6.018 μM), SW1271 (EC_50_ = 6.699 μM)	Small cell lung cancer	NCT00445198 (phase 1/2)	^83^
ABT-199 (Venetoclax)	Bcl-2↓	Induce apoptosis	NCI-H510A and DMS-53	Small cell lung cancer	NCT02391480 (phase 1)	^85^
AT-101	Bcl-2, Mcl-1↓	Induce apoptosis	YES-6, GT5, YCC1 cells	Gastro-esophageal cancers	NCT00561197 (phase 1/2)	^86^
Verteporfin	Bcl-2, cyclinD1, cyclinE1↓	Induce apoptosis	PANC-1 (IC_50_ = 1.4 μM), SW1990 (IC_50_ = 1.7 μM)	Pancreatic ductal adenocarcinoma	NCT03033225 (phase 2)	^88^
(E)-2-benzylidene-3-(cyclohexylamino)-2,3-dihydro-1H-inden-1-one (BCI)	Bcl-2↓ Bax↑ Cyt-C↑	Induce apoptosis	NCI-H1299, A549, and NCI-H460	Lung cancer		^89^
4-((4-acetylphenyl)sulfonyl)-1-(4-(4-chloro-3,5-dimethylphenyl)butyl)-3,5-dimethyl-1H-pyrrole-2-carboxylic acid	Mcl-1↓	Induce apoptosis	H929 (IC_50_ = 0.36 ± 0.09 μM), MV4-11 (IC_50_ = 0.70 ± 0.07 μM) SK-BR-3 (IC_50_ = 2.84 ± 0.66 μM) NCI-H23 (IC_50_ = 3.02 ± 1.35 μM)	Myeloid leukemia		^92^
AZD5991	Mcl-1↓	Induce apoptosis	Mcl-1 (IC_50_ = 0.72 nM)	Multiple myeloma, acute myeloid leukemia	NCT03218683 (phase 1/2)	^93^
Garciniaxanthone I	Bcl-2, Bcl-xL, Mcl-1↓ Bax↑	Induce apoptosis	HepG2 (IC_50_ = 24.61 ± 1.89 μM)	Liver cancer		^94^
Ampelopsin	Bcl-2, Bcl-xL↓ Bax↑	Induce apoptosis	K562 (IC_50_ = 135.2 μM), HL60 (IC_50_ = 45.1 μM)	Acute myeloid leukemia, chronic myeloid leukemia		^95^
5,3′-dihydroxy-3,6,7,8,4′-pentamethoxyflavone (PMF)	Bcl-2↓ Bax↑ Cyt-C↑	Induce apoptosis	MCF-7 (IC_50_ = 1.5 μM)	Breast cancer		^96^
Tracheloside	Bcl-2, Bcl-xL↓ Bax↑	Induce apoptosis	CT26	Colorectal cancer		^97^
Deoxypodophyllotoxin	Bcl-xL↓ Bax↑	Induce apoptosis	HT29, DLD1, Caco2	Colorectal cancer		^98^
Trimethoxyphenyl-1,2,3-triazole-coumarin 19c	Bax, DR5↑ Bcl-xL↓	Induce apoptosis	PC3 (IC_50_ = 0.34 ± 0.04 μM), MGC803 (IC_50_ = 0.13 ± 0.01 μM) HepG2 (IC_50_ = 1.74 ± 0.54 μM)	Prostate cancer, gastric cancer, liver cancer		^99^
(10R,13S,17R)-N-(2-((E)-3-(2-methoxyphenyl)acrylamido)ethyl)-10,13-dimethyl-3-oxo-6,7,8,9,10,11,12,13,14,15,16,17-dodecahydro-3H-cyclopenta[a]phenanthrene-17-carboxamide	Bcl-2↓ Bax↑ caspase-3↑	Induce apoptosis	MDA-MB-231 (IC_50_ = 0.42 μM)	Triple negative breast cancer		^100^
Pt(IV) complex 14 and 17	Bcl-2↓ Bax, Cyt-C↑	Induce apoptosis	HepG-2 (14: IC_50_ = 2.23 μM; 17: IC_50_ = 4.65 μM) NCI-H460 (14: IC_50_ = 0.97 μM; 17: IC_50_ = 3.66 μM)	Liver cancer, lung cancer		^102^
[Ir(ppy)_2_(THPDP)]PF_6_ (Ir-1)	Bcl-2↓ Bax, Bad↑	Induce apoptosis	B16 (IC_50_ = 1.0 ± 0.02 μM), A549 (IC_50_ = 1.4 ± 0.03 μM), Eca-109 (IC_50_ = 1.6 ± 0.06 μM)	Melanoma, lung cancer, esophagus cancer		^104^
[Cu(Gly-*L*-leu)(HPBM)(H_2_O)]ClO_4_	ROS, Bax↑ Bcl-2↓	Induce apoptosis	HeLa (IC_50_ = 7.88 ± 0.3 μM), A549 (IC_50_ = 8.39 ± 0.4 μM), PC-3 (IC_50_ = 11.49 ± 0.6 μM)	Cervical cancer, lung cancer, prostatic cancer		^105^
CYD-4-61	Bax, Cyt-C↑	Induce apoptosis	MDA-MB-231 (IC_50_ = 0.07 μM), MCF-7 (IC_50_ = 0.06 μM)	Breast cancer		^106^
HG30	Mcl-1, surviving, XIAP↓ Bid, Bim↑	Induce apoptosis	A549 (IC_50_ = 2.808 ± 0.09 μM) H1299 (IC_50_ = 0.545 ± 0.113 μM)	Lung caner		^107^
Licochalcone A	Bax/Bcl-2, Cyt-C, ROS↑	Induce apoptosis	T24 (IC_50_ = 40.23 μM) 5637 (IC_50_ = 42.47 μM)	Bladder cancer		^108^
(Z)-3-(morpholinomethyl)-5-(3,4,5-trimethoxybenzylidene)thiazolidine-2,4-dione	Bcl-2, Bcl-xL, Mcl-1↓ Bak, Bax, Bim↑	Induce apoptosis	MCF-7 MDA-MB-231 (average: IC_50_ = 1.27 μM)	Breast cancer		^109^
(E)-2-hydroxy-5-(3-(3,4,5-trimethoxyphenyl)acryloyl)benzamide	ROS↑ Bcl-2↓	Induce apoptosis	HepG2 (IC_50_ = 38.33 μM)	Liver cancer		^110^
7-([1,1′-biphenyl]-4-ylmethoxy)-6,8-dibromo-N-((4-chloro-3-nitrophenyl)sulfonyl)-2-(4-cyanobenzyl)-1,2,3,4-tetrahydroisoquinoline-3-carboxamide	Bcl-2, Bcl-xL↓	Induce apoptosis	Jurkat (IC_50_ = 15.5 ± 1.5 μM)	Leukemia		^111^
Dioscin	Akt1, Bcl-2↓ Bax, Cyt-C, Apaf-1↑	Induce apoptosis	ASPC-1 PANC-1 (IC_50_ = 2.9–5.8 μM)	Pancreatic cancer		^112^
Cinobufagin	Bcl-2↓ Bax, Cyt-C, Apaf-1↑	Induce apoptosis	HK-1 (IC_50_ = 0.061 μM)	Nasopharyngeal carcinoma		^113^

↓ decrease/inhibition, ↑ increase/activation

#### Targeting Apaf-1 and Cyt-C

Cyt-C, a member of the mitochondrial electron transport chain, is a vital factor promoting cell apoptosis.^114^ Apaf-1 is a cytoplasmic protein with a cysteine protease recruitment domain at its amino-terminal, which is the core of apoptosome and plays a pivotal role in activating the caspase cascade. Cyt-C/Apaf-1 are the essential classical pathway in the initiation of apoptosis in mitochondria.^115,116^ In the process of apoptosis, the permeability of mitochondrial intima was increased, and the mitochondrial membrane potential was reduced, which promoted the release of pro-apoptotic factors such as Cyt-C, Apaf-1, and AIF from the mitochondria membrane space into the cytoplasm and then bound with Apaf-1 to activate downstream caspase 9 and caspase 3, and finally, lead to the occurrence of apoptosis.^117^

Macrophage migration inhibitory factor (MIF) is a pro-inflammatory factor overexpressed in several solid tumors to accelerate tumor development and metastasis. CPSI-1306 as a MIF inhibitor was found to decrease tumor growth and metastasis both in vitro and in vivo. CPSI-1306 enhanced ROS levels in TNBC cells and promoted the release of Cyt-C and AIF from mitochondria, leading to the induction of cell apoptosis.^118^ Ginseng is a kind of traditional Chinese medicine with various pharmacological activities. Piperazine groups introduced into ginsenoside can improve ROS generation and stimulate the apoptosis of cancer cells.^119^ In this work, xiao et al. synthesized a series of novel ginsenoside piperazine derivatives and tested the antiproliferative activity against PC-3 cells. Ginsenoside piperazine derivative 6g with an IC_50_ value of 1.98 ± 0.34 μM was identified as the most potent compound. It could induce apoptosis in PC-3 cells, and this induction was mediated by the enhanced ROS production and Cyt-C release. It could also upregulate the expression of Cl-PARP, Cl-Caspase-3, and Cl-Caspase-9, leading to apoptosis.^119^ Moreover, ((E)-2-(3-benzyl-4,4-dimethyl-2-oxooxazolidin-5-ylidene)-N,N-diethylacetamide (OI), the derivative of oxazolidinones, exhibited good antitumor activity. OI treatment could activate ROS generation, causing the reduction of mitochondrial membrane potential, further increasing the expression of caspase 9 and the release of Cyt-C, leading to apoptosis of MCF-7 and HeLa cells.^120^ Compound 19b as a boehmeriasin A derivative was found to trigger apoptosis of liver cancer cell lines by promoting the release of Cyt-C, the cleavage of PARP, and arrest of the cell cycle at the SubG1 phase.^121^

Additionally, ethyl 5-(2-cyano-3-(furan-2-yl)acrylamido)-1,3-diphenylpyrazole-4-carboxylate 5 as a synthesized compound had shown significant cytotoxic effect against colon cancer and initiated the intrinsic apoptosis through increasing the protein level of Cyt-C, Apaf-1, and SMAC/DIABLO. Besides, other apoptosis-related genes had also been stimulated, such as Bax, Bcl-2, p53, MMP-1, etc.^122^

Traditional Chinese medicine or natural medicine possesses tremendous development potential in anticancer drug research. For example, baicalein (BA), a natural flavonoid compound, was shown to inhibit the viability of A549 and H1299 lung cancer cells and trigger apoptosis. BA made the mitochondrial impairment, which would cause changes in mitochondrial membrane potential, as well Cyt-C and AIF in mitochondria would be released into the cytoplasm. In addition, BA could also activate the adenosine 5′-monophosphate-activated protein kinase (AMPK)/mitochondrial fission pathway to elicit apoptosis and autophagy.^123^ Hexokinase 2 (HK2) is overexpressed in human cancers, accelerating glucose uptake and forming the HK2-VDAC complex to generate apoptosis resistance.^124^ Liu et al. found that xanthohumol exerted a significant antitumor effect on CRC cells by downregulating HK2 expression and suppressing glycolysis. Besides, it could promote the release of Cyt-C to activate apoptosis.^125^ Lycorine, as a natural compound isolated from the *Amaryllidaceae* plant, was shown to induce mitochondrial apoptosis in HepG2 cells via the promotion of Cyt-C release into the cytosol.^126^ Natural products, curcumin and emodin, also exhibited significant antitumor effects against melanoma and liver cancer cells, respectively.^127,128^ Except as mentioned above, oroxyloside, the metabolite of oroxylin A, was reported to be a promising agent for human glioma treatment. It was found that apoptosis was suppressed by oroxyloside by improving the cleavage of caspase 9, caspase 3, and PARP, as well as promoting the release of Cyt-C into the cytoplasm and increase of Apaf-1^129^ (Table 3).

**Table 3:** Compounds targeting Apaf-1 and Cyt-C in cancer

Compound name	Target	Mechanism in RCD	Cancer cell line (activity)	Indication of tumor type	Clinical trial identifier	Ref.
CPSI-1306	ROS, Cyt-C↑	Induce apoptosis	MDA-MB-468 (IC_50_ = 0.84 μM), MDA-MB-231 (IC_50_ = 1.16 μM)	Triple negative breast cancer		^118^
Ginsenoside piperazine derivative 6g	ROS, Cyt-C↑	Induce apoptosis	PC-3 (IC_50_ = 1.98 ± 0.34 μM)	Prostate cancer		^119^
((E)-2-(3-benzyl-4,4-dimethyl-2-oxooxazolidin-5-ylidene)-N,N-diethylacetamide (OI)	Cyt-C↑	Induce apoptosis	MCF-7 (IC_50_ = 17.66 μM), HeLa (IC_50_ = 31.10 μM)	Cervix adenocarcinoma, breast cancer		^120^
Compound 19b	Cyt-C↑	Induce apoptosis	Huh7 (IC_50_ = 0.002 μM), Hep3B (IC_50_ = 0.017 μM)	Liver cancer		^121^
Ethyl 5-(2-cyano-3-(furan-2-yl)acrylamido)-1,3-diphenylpyrazole-4-carboxylate 5	Cyt-C, Apaf-1↑	Induce apoptosis	HCT116 (IC_50_ = 30.6 μM)	Colon cancer		^122^
Baicalein	Cyt-C, AIF↑	Induce apoptosis	A549 H1299	Lung cancer		^123^
Xanthohumol	Cyt-C↑	Induce apoptosis	HCT116 HT29	Colorectal cancer	NCT02432651 (phase 1)	^125^
Lycorine	Cyt-C↑	Induce apoptosis	HepG2 (IC_50_ = 10–20 μM)	Liver cancer		^126^
Curcumin	Cyt-C↑	Induce apoptosis	A375 (IC_50_ = 40 μM)	Melanoma		^127^
Emodin	Cyt-C, Apaf-1↑	Induce apoptosis	Bel-7402	Liver cancer		^128^
Oroxyloside	Cyt-C, Apaf-1↑	Induce apoptosis	U87-MG (IC_50_ = 36.87 μM) U251-MG (IC_50_ = 52.36 μM) U138-MG (IC_50_ = 59.67 μM)	Human glioma		^129^

↓ decrease/inhibition, ↑ increase/activation

#### Targeting NF-κB

NF-κB, a crucial nuclear transcription factor in cells, participates in various complex biological processes such as cell proliferation, invasion, and angiogenesis.^130,131^ NF-κB is also a crucial regulatory member of the anti-apoptosis signaling pathway. Typically, NF-κB exists in the cytoplasm in the form of dimer and binds to the specific inhibitor IκBα. Upon certain stimuli, IκBα is phosphorylated and degraded in a proteasome-dependent manner, which releases NF-κB and ultimately transfers to the nucleus, regulating the transcription of target genes, thus affecting the occurrence and development of tumors.^132,133^ Blocking the activity of NF-κB could change the survival/death balance of tumor cells. Therefore, targeting NF-κB to induce apoptosis of cancer cells has been considered to be an effective way to treat human cancers. We have collected several potential small-molecule compounds to treat human cancers by targeting the NF-κB pathway.

Selinexor has been shown to inhibit XPO1-mediated nuclear export from exerting anticancer activity. Survivin was an inhibitor of apoptosis, which could be regulated by the transcription of NF-κB. Selinexor attenuates the growth of cancer cells and induces selinexor-resistant cells to become sensitive to selinexor by stabilizing IκB to inhibit the NF-κB pathway and then downregulating the expression of survivin protein. Nair et al. reported that selinexor A has an inhibitory effect on various sarcoma cells with IC_50_ values ranging from 50 nM to 2.5 μM, among them the liposarcoma (LS141) showing the most substantial sensitivity. Besides, when combined with proteasome inhibitor carfilzomib, it caused resistant cell lines to be more sensitive to Selinexor.^134^ A phase I clinical trial (NCT01607905) showed that selinexor possessed antitumor activity against advanced solid tumors. Among the 157 patients treated, 7 had reduced target lesions, and 27 had stable disease control for four months or longer.^135^ MicroRNAs (miRNAs), as a class of non-coding ribonucleic acid (RNA) molecules, could directly participate in mRNA expression or inhibit the translation processes from regulating the related-protein level, which was also associated with cell apoptosis, proliferation, and metastasis. Puerarin, a flavonoid compound, had been observed in bladder cancer cell lines to significantly inhibit cell viability and proliferation, inactivating the NF-κB pathway by upregulating the mRNA level of mir-16 and thereby enhancing cell apoptosis.^136^

The emergence of multidrug resistance (MDR) was a major obstacle to treating cancer diseases.^137^ Studies have shown that NF-κB could increase the expression of the MDR1 gene, and then inhibition of NF-κB activity could enhance the sensitivity of drug-resistant cancer cells to chemotherapy drugs.^138^ Abdin et al. proposed that the combination of NF-κB inhibitors (Pentoxifylline/Bortezomib) and DOX could reduce DOX resistance in breast cancer cells. DOX/NF-κB inhibitor combination therapy could inhibit the migration of cancer cells, activate apoptosis-related proteins, and induce cell apoptosis. It could be an effective therapeutic strategy for overcoming multidrug resistance in cancer cells.^139^ Radiotherapy was the main treatment for non-small cell carcinoma, while radioresistance would be induced, leading to a low reactivity.^140^ The research showed that isorhamnetin (ISO), a flavonoid extracted from *Hippophae L*., could be a potent natural radiosensitizer to increase the incidence of apoptosis, the change of MMP, as well as suppress the upregulation of NF-κB p65 triggered by irradiation in A549 cells. Additionally, the expression of interleukin-13 (IL-13) was related to the strength of ISO-mediated radiosensitization. Therefore, ISO treatment could enhance the expression of IL-13.^141^

Aurantoside C (C828), a natural product obtained from marine sponge *Manihinea lynbeazleyae*, had shown potent cytotoxic activity against TNBC cells.^142^ C828 was found to trigger apoptosis by inhibiting the phosphorylation of Akt/mTOR and NF-κB pathways and upregulating the expression of p38/MAPK and SAPK/JNK pathways without cytotoxic effects on normal cells. Furthermore, C828 exhibited a 20-fold and 35-fold higher cytotoxic effect than chemotherapeutic drugs DOX and cisplatin. Therefore, C828 could be a promising lead compound for developing anti-TNBC agents.^142^ Another natural flavonoid glycoside product, hyperoside (quercetin 3-o-β-d-galactopyranoside), exhibited antitumor, antidepressant, and anti-inflammatory effects. It was reported to suppress the viability and migration ability of MCF-7 and 4T1 cells, as well as promote apoptosis through the inhibition of ROS generation, further suppressing the activation of the NF-κB signaling pathway. Besides, the finding from the in vivo study suggested that it could decrease the size of tumors in mouse model.^143^ Arnicolide D (Ar-D), a sesquiterpene lactone compound, was shown to exert an anti-melanoma effect. This effect was mediated by inhibiting the expression of IKKα/β, the degradation of IκBα, and the phosphorylation of NF-κB p65, ultimately eliciting the apoptosis of melanoma cells.^144^ Furthermore, Glaucocalyxin A (GLA) could also induce mitochondrial apoptosis through the inhibition of the NF-κB/p65 pathway.^145^ Other natural small-molecule compounds such as cardamonin, ginsenoside Rk1, and betulinic acid exhibited potent anticancer effects and triggered cancer cell apoptosis through the inhibition of the NF-κB pathway^146–148^ (Table 4).

**Table 4:** Compounds targeting NF-κB in cancer

Compound name and structure	Target	Mechanism in RCD	Cancer cell line (activity)	Tumor type	Clinical trial identifier	Ref.
Selinexor	NF-κB↓	Induce apoptosis	CHP100; LS141; MPNST; SK-UT1 (IC_50_ = 50 nM–2.5 μM)	Liposarcoma, uterine leiomyosarcoma	NCT02269293 (phase 1)	^134^
Puerarin	miR-16↑ NF-κB↓	Induce apoptosis	T24	Bladder cancer		^136^
Pentoxifylline/ Bortezomib	NF-κB↓	Induce apoptosis	MDA-MB-231 (PTX: IC_50_ = 2.6 ± 1.2 μM; BTZ: IC_50_ = 8 ± 1.2 μM) MCF-7 (PTX: IC_50_ = 3 ± 0.06 μM; BTZ: IC_50_ = 7 ± 1.1 μM)	Breast cancer	NCT00188669 (phase 2); NCT00028639 (phase 2)	^139^
Isorhamnetin	NF-κB p65↓	Induce apoptosis	A549 (IC_50_ = 40 μM) H460 (IC_50_ = 50 μM)	Non-small cell lung cancer		^141^
Aurantoside C	Akt/mTOR,NF-κB↓	Induce apoptosis	SUM159PT (IC_50_ = 0.56 ± 0.01 μM) MDA-MB-231 (IC_50_ = 0.61 ± 0.01 μM) SUM149PT (IC_50_ = 0.81 ± 0.02 μM)	Triple negative breast cancer		^142^
Hyperoside	ROS, NF-κB↓	Induce apoptosis	MCF-7 4T1	Breast cancer		^143^
Arnicolide D	NF-κB/p65↓	Induce apoptosis	A375 B16F10 (IC_50_ = 1–2 μM)	Melanoma		^144^
Glaucocalyxin A	NF-κB/p65↓	Induce apoptosis	A375 (IC_50_ = 18.21 μM) A2058 (IC_50_ = 20.28 μM)	Melanoma		^145^
Cardamonin	NF-κB mTOR↓	Induce apoptosis	SKOV3 (IC_50_ = 8.04 μM) PDC (IC_50_ = 45.87 μM)	Ovarian cancer		^146^
Ginsenoside Rk1	NF-κB↓	Induce apoptosis	A549 (IC_50_ = 69.25 μM) PC9 (IC_50_ = 66.12 μM)	Lung adenocarcinoma		^147^
Betulinic acid	NF-κB↓	Induce apoptosis	U266 RPMI 8226	Multiple myeloma		^148^

↓ decrease/inhibition, ↑ increase/activation

#### Targeting p53

The p53 protein, a notable tumor suppressor, plays a principal role in the regulation of cell cycle arrest, cell differentiation, cell metastasis, and apoptosis.^149^ In response to cellular stress, p53 is activated and then exerts its transcriptional regulatory function to influence the level of Bcl-2 family protein, resulting in increased cellular level of pro-apoptotic members of the Bcl-2 family and concomitantly decreased level of anti-apoptotic proteins, thereby preventing malignant transformation and leading to apoptotic cell death.^150^ A large number of studies had reported that the dysfunction of p53 owing to the mutation of p53 protein was observed in ~50% of all human tumors, which was always accompanied by angiogenesis, tumor progression, and drug resistance.^151^ Mutations in tumor suppressor proteins could lead to the loss of tumor suppressor function or promote the acquisition of new cancer phenotypic functions, such as excessive proliferation, invasive enhancement, and chemo-resistance.^152,153^ Therefore, targeting mutant p53 with small-molecule compound to induce apoptosis could be an attractive strategy for the development of anticancer therapy.

Murine double minute 2 (MDM2) is a negative regulator directly bound to p53 protein and inhibits the activation of p53, causing aberrant cell proliferation and growth.^154^ The report showed that p53 protein could be degraded by the complex of human papillomaviruses (HPV) oncoprotein E6 and E6-associated protein (E6AP) ubiquitin ligase.^155^ MDMX could inhibit the trans-activation of p53-mediated target genes and increase the activity of MDM2 through stabilization.^156,157^ XI-011 (NSC146109) was an MDMX inhibitor that could trigger apoptosis and suppress the growth of cervical cancer cells by restoring the stability of p53 and enhancing its transcription activity.^158^ And a new tryptophanol-derived oxazoloisoindolinone, DIMP53-1, bound to p53 suppressing its interaction with MDM2 and MDMX, which inhibit the migration and invasion of colon adenocarcinoma HCT116 cell.^159^ p73 was a tumor suppressor whose structure and function were similar to p53 protein, but it rarely mutated in cancers and was easily inactivated when combined with MDM2, MDM4, etc.^160^ Protoporphyrin IX (PpIX), as a metabolite of aminolevulinic acid, could inhibit the p53/MDM2 and p53/MDM4 interactions, and further elicit the accumulation of p53 and Tap73 to promote apoptosis in CLL cells.^161^ Therefore, targeting MDMX-mediated inhibition of p53 functions as a novel anticancer drug development strategy. Additionally, there are also numerous small molecule compounds that target to inhibit MDM2-activated p53. For example, APG-115 was a novel MDM2/p53 inhibitor, and the report showed that it could improve the radiosensitivity effects in gastric cancer cells by regulating the expression of MDM2-p53 pathway-related proteins.^162^ It also observed that cell apoptosis increased and cell cycle arrested after treatment of APG-115. Besides, APG-115 combined with radiosensitivity could enhance the antitumor activity both in vitro and in vivo.^162^ HL001 was a Cyclophilin A (CypA) inhibitor, which restored the expression of p53 by suppressing the MDM2-mediated ubiquitination of p53, thus inducing the cell cycle arrest and apoptosis of NSCLC cells.^163^ Similarly, anthraquinone (AQ) analog, AQ-101, promoted the apoptosis of acute lymphoblastic leukemia (ALL) cells via downregulating the MDM2 level to activate p53 protein.^164^

Mortalin/GRP75 belongs to the heat-shock protein (Hsp70) family, which is found to overexpress in several cancers like ovarian, colorectal, and hepatocellular carcinoma. p53 protein binding to mortalin could inhibit its translocation, further eliminating its tumor suppression function.^165^ Therefore, Sari et al. synthesized a new small compound, Mortaparib (Plus), which was shown to block the interaction between mortalin and p53, as well as reactivate the p53 expression, causing the induction of apoptosis in CRC cells.^166^ Moreover, Mortaparib (Plus) could also activate the p53 pathway to induce apoptosis in MCF-7 breast cancer cells.^167^ In some cancers, c-Myc could induce stem cells, suppress cell senescence and differentiation, and promote the survival of eliminating leukemic stem cells (LSCs) in leukemia. A small-molecule compound, DJ34, was reported to inhibit the transcription of c-Myc and activate p53 protein to selectively and synergistically eliminate LSCs.^168^ In human ovarian cancer, placenta-specific protein 1 (PLAC1) was overexpressed and could cause cell proliferation and metastasis. p53 inhibited PLAC1 transcription, while mutation of p53 attenuated this effect. Studies have shown that the p53 agonist, HO-3867, could restore the transcription inhibition of PLAC1 by mutant p53 in ovarian cancer cells, inhibit cell growth, and ultimately induce apoptosis. Treatment of ovarian cancer with HO-3867 may be used as adjunctive therapy to improve outcomes in these patients.^169^ Another compound, andrographlide (ANDRO), was reported to suppress the expression of mutant p53 and promote the combination of the Hsp70 and mutant p53, further inducing proteasomal degradation of p53 and ultimately inhibiting cell growth.^170^

Some natural and semi-synthetic compounds were reported to target p53 protein to induce apoptosis. For example, renieramycin T (RT) was a tetrahydroisoquinoline alkaloid obtained from the Thai blue sponge *Xestospongia* sp. RT treatment could significantly activate the expression of p53 and induce the degradation of McL-1 in lung cancer cells, thereby triggering apoptosis.^171^ Protopine was a natural isoquinoline alkaloid that could stabilize p53 protein to enhance the p53-mediated transcriptional level, leading to the induction of apoptosis and inhibition of HCT116 colon cancer cells proliferation.^172^ Furthermore, Actinomycin V and TCCP could also activate p53 expression to trigger cancer cell apoptosis.^173,174^ Reddy et al. had synthesized a series of methyl β-orsellinate based 3, 5-disubstituted isoxazole hybrids and tested the proliferative activity against four human cancer cell lines. Among them, compound 12 was the most active hybrid with an IC_50_ value of 7.9 ± 0.07 μM against MCF-7 cells. It could block the cell cycle at the G2/M phase and induce apoptosis through the activation of p53 and PTEN, further promoting the expression of Bax and Cyt-C.^175^ It was also found that indolizine derivatives, compound 3, and resveratrol derivative, trans-3, 5, 4′-trimethoxystilbene (TMS), promoted the activation of p53, causing the apoptosis of cancer cells.^176,177^ TMS cotreatment with TRAIL could reverse the resistance to apoptosis in cells.^177^

Several studies proved that the p53 pathway could involve gold complexes mediated apoptosis. [di-(1,3-diethylbenzylimidazol-2-ylidene)] gold(I) iodide (MC3) as a gold(I) NHC complex had shown potent cytotoxic effects against CRC cell lines with different p53 profiles. MC3 treatment could upregulate ROS level and p21 expression, whatever the status of p53, but with WT p53 cells exhibiting the highest pro-apoptotic activity.^178^ Ma et al. introduced 6-bromocoumarin-3-carboxylic acid into Pt(IV) complex to synthesize bromocoumarinplatin 1. It was found that bromocoumarinplatin 1 could also activate p53 protein to enhance the anticancer activity and overcome the resistance of cisplatin through the p53 pathway.^179^ Diplatin, a novel platinum complex, had shown that the antitumor activity of diplatin against lung cancer cell lines was superior to that of carboplatin. In the mouse xenotransplantation model, diplatin could significantly improve some therapeutic indicators and inhibit the growth of lung cancer cells that were resistant to cisplatin. Diplatin induced tumor cell apoptosis by activating ROS/JNK/p53 signaling pathway. Accordingly, compared with cisplatin and carboplatin, diplatin had better therapeutic efficiency and safety^180^ (Table 5).

**Table 5:** Compounds targeting p53 in cancer

Compound name and structure	Target	Mechanism in RCD	Cancer cell line (activity)	Tumor type	Ref.
XI-011 (NSC146109)	E6-E6AP-p53↑	Induce apoptosis	Hela Caski Siha (IC_50_ = 0.5–1.0 μM)	Cervical cancer	^158^
DIMP53-1	p53↑	Induce apoptosis	HCT116	Colon adenocarcinoma	^159^
Protoporphyrin IX	p53, Tap73↑	Induce apoptosis	EHEB (IC_50_ = 2.5 μM) HL60 (IC_50_ = 2.4 μM)	Chronic lymphocytic leukemia	^161^
APG-115	p53↑	Induce apoptosis	AGS (IC_50_ = 18.9 ± 15.6 nM) MKN45 (IC_50_ = 103.5 ± 18.3 nM)	Gastric cancer	^162^
HL001	p53↑	Induce apoptosis	A549 H460 H292	Non-small cell lung cancer	^163^
AQ-101	p53↑	Induce apoptosis	EU-1 EU-3 EU-8 (IC_50_ = 0.25–0.5 μM)	Acute lymphoblastic leukemia	^164^
Mortaparib (Plus)	p53↑	Induce apoptosis	HCT116 (IC_50_ = 4–5 μM) DLD-1 (IC_50_ = 2–3 μM)	Colorectal cancer	^166^
DJ34	p53↑ c-Myc↓	Induce apoptosis	BCR-Abl cells	Chronic myeloid leukemia, acute lymphoid leukemias	^168^
HO-3867	p53↑	Induce apoptosis	OVCAR3 ES-2	Ovarian cancer	^169^
Andrographlide	mutant p53↓	Induce apoptosis	PANC-1 HCT116 MKN45 (IC_50_ = 25–40 μM)	Pancreatic cancer, colorectal cancer, gastric cancer	^170^
Renieramycin T	p53↑ Mcl-1↓	Induce apoptosis	H460 (IC_50_ = 1.93 ± 0.4 μM) H292 (IC_50_ = 0.88 ± 0.06 μM) H23 (IC_50_ = 2.47 ± 0.14 μM) A549 (IC_50_ = 3.77 ± 0.38 μM)	Lung cancer	^171^
Protopine	p53↑	Induce apoptosis	HCT116	Colon cancer	^172^
Luteoloside	p53↑	Induce apoptosis	Hela cells	Cervical cancer	^489^
Actinomycin V	p53↑	Induce apoptosis	BEAS-2B (IC_50_ = 4.2 ± 0.48 μM) A549 (IC_50_ = 0.68 ± 0.06 μM) NCI-H1299 (IC_50_ = 16.37 ± 1.07 μM)	Non-small-cell lung carcinoma	^173^
TCCP	p53↑	Induce apoptosis	MDA-MB-231	Triple negative breast cancer	^174^
Compound 12	p53, PTEN↑	Induce apoptosis	MCF-7 (IC_50_ = 7.9 ± 0.07 μM)	Breast cancer	^175^
Indolizine derivative 3	p53↑	Induce apoptosis	HepG2 (IC_50_ = 25–50 μM)	Liver cancer	^176^
Trans-3, 5, 4′-trimethoxystilbene (TMS)	p53↑	Induce apoptosis	Saos-2 (IC_50_ = 100–200 nM)	Osteosarcoma	^177^
MC3	p53, p21, ROS↑	Induce apoptosis	HCT116 WT (IC_50_ = 0.62 ± 0.26 μM) HCT116 p53−/− (IC_50_ = 0.99 ± 0.27 μM) HT-29 (IC_50_ = 1.67 ± 0.62 μM)	Colorectal cancer	^178^
Bromocoumarinplatin 1	p53↑	Induce apoptosis	HCT-116 (IC_50_ = 3.94 ± 0.39 μM) MCF-7 (IC_50_ = 7.85 ± 1.45 μM)	Colon cancer, breast cancer	^179^
Diplatin	ROS/JNK/p53↑	Induce apoptosis	HCC827 (IC_50_ = 25.0 μM) H292 (IC_50_ = 30.9 μM) LTEP-A-2 (IC_50_ = 24.6 μM)	Lung cancer	^180^

↓ decrease/inhibition, ↑ increase/activation

#### Other targets

The mitogen-activated protein kinase (MAPK) signaling pathway has a critical role in regulating cell growth, proliferation, differentiation, and apoptosis.^181^ Abnormal expression of MAPK in tumor cells may cause the uncontrolled proliferation and resistance to apoptosis of tumor cells. MAPK could be classified into three distinct cascades: ERK1/2, JNK1/2, and p38 MAPK.^182,183^ Therefore, some small-molecule compounds that target MAPK related signaling pathway provide a promising strategy for cancer treatment. Cudraflavone C has been shown to activate the MAPKs pathway (the phosphorylation of p38, ERK, and JNK) to enhance the expression of apoptotic proteins, thus leading to the apoptosis of melanoma cells.^184^ Celastrol, a natural triterpene compound, could induce apoptosis of nasopharyngeal carcinoma cells and oral cancer cells by increasing the expression level of p38/MAPK, ERK1/2, and activating the JNK1/2 signaling pathway, respectively.^185,186^ Diallyl trisulfide (DATS)-mediated antitumor activity involved multiple pathways that induced apoptosis by activating the JNK/p38 MAPK pathway and decreasing Nrf2 and Akt protein expression. When DAT was combined with cisplatin (DDP), the antitumor activity was increased, with side effects decreased.^187^ Another one, Avicequinone-B, a furanonaphthoquinone derivative with poor water solubility, was prepared into liposomes to improve the anticancer activity against cutaneous squamous cell carcinoma (SCC) cells. Liposomal Avicequinone-B promoted the induction of apoptosis by activating the ERK, p38, and JNK pathways.^188^

AMPK is a serine/threonine protein kinase that regulates downstream signal molecules by phosphorylation to exert its biological activity. AMPK could induce tumor cell apoptosis through different signaling pathways.^189^ In CRC cell lines, gomisin A was shown to trigger caspase-dependent apoptosis through the regulation of the AMPK/p38 pathway.^190^ Metformin is a hypoglycemic agent in treating type 2 diabetes, and previous studies have shown that it could exert antitumor effects by regulating the AMPK pathway.^191^ Lu et al. investigated the anti-proliferation and pro-apoptotic activities of metformin. It showed that metformin could trigger apoptosis in AGS cells by increasing phosphorylation of AMPK, inhibiting phosphorylation of ERK, p38, and JNK.^192^

The phosphatidylinositol 3-kinase/protein kinase B/mammalian target of rapamycin PI3K/Akt/mTOR pathway is one of the most important signaling pathways regulating cell progression,^193^ which is overactivated in the occurrence and development of cancers^194^ PKI-402 was a dual PI3K/mTOR inhibitor that inhibited the growth of cisplatin-resistant ovarian cancer epithelial cell SKOV3. It could enhance the degradation of anti-apoptotic protein Mcl-1 via the inhibition of the PI3K/Akt/mTOR signaling pathway, thus promoting the apoptotic pathways in SKOV3 cells.^195^ 1,7-Bis(4-hydroxyphenyl)-1,4-heptadien-3-one (EB30) was a curcumin analog that could block the cell cycle and activate ROS production, which induced lung cancer cells apoptosis by inhibiting the PI3K/Akt pathway and activating the ERK1/1 pathway.^196^ Apigenin 7-O-glucoside (AGL) was shown to trigger apoptosis of HeLa cells via the inhibition of the PTEN/PI3K/Akt pathway in a concentration-dependent manner.^197^

Small molecules inhibit the overexpression of the Wnt/β-catenin pathway in cancer cells to regulate cell proliferation and apoptosis.^198^ C644-0303, a small-molecule inhibitor, was shown to suppress the Wnt/β-catenin pathway to inhibit cell migration and induce apoptosis in CRC cell lines.^199^ Obatoclax as a pan-Bcl-2 inhibitor had been shown to obstruct BH3-mediated binding of BH3-only proteins or Bax/Bak to induce apoptosis. In this study, obatoclax could suppress the expression of anti-apoptotic protein surviving and inhibit the activation of the Wnt/β-catenin signaling pathway, further triggering apoptosis in HCT116 cells.^200^

Generally, the expression, phosphorylation, or activation of signal transducer and activator of transcription 3 (STAT3) would induce apoptosis and prevent tumor progression.^201^ CYT997 could enhance the ROS accumulation and trigger apoptosis by inhibiting the JAK2-STAT3 pathway.^202^ Licochalconce H (LCH), a synthesized compound, exhibited a potent antiproliferative effect and induced apoptosis in oral squamous cell carcinoma (OSCC) cells via the suppression of JAK2-STAT3 signaling pathway.^203^ AH057, a JAK inhibitor, was reported to induce apoptosis by blocking the JAK-STAT pathways^204^ (Table 6).

**Table 6:** Compounds targeting other targets of apoptosis in cancer

Compound name and structure	Target	Mechanism in RCD	Cancer cell line (activity)	Tumor type	Clinical trial identifier	Ref.
Cudraflavone C	ROS↑ p38 MAPK pathway↑	Induce apoptosis	A375.S2 (IC_50_ = 3.42 μM)	Melanoma		^184^
Celastrol	p38 MAPK↑ ERK1/2↑	Induce apoptosis	NPC-039 NPC-BM	Nasopharyngeal carcinoma		^185^
	JNK1/2 pathway		SASV16 (IC_50_ = 0.5–1.0 μM)	Oral cancer		^186^
Diallyl trisulfide	Nrf2/Akt↓ JNK/p38 MAPK↑	Induce apoptosis	BGC-823 (IC_50_ = 115.2 ± 4.3 μM)	Gastric cancer		^187^
Avicequinone-B	p38 MAPK pathway↑	Induce apoptosis	HSC-1 (IC_50_ = 1–2 μM)	Human cutaneous squamous cell carcinoma		^188^
Gomisin A	AMPK/p38 pathway↑	Induce apoptosis	CT26 MC38 HT29 SW620	Colorectal cancer		^190^
Metformin	AMPK↑ Akt/mTOR↓	Induce apoptosis	AGS (IC_50_ = 30–40 mM)	Gastric cancer	NCT04033107 (phase 2)	^192^
PKI-402	PI3K/Akt/mTOR↓	Induce apoptosis	SKOV3 (IC_50_ = 4–5 μM)	Ovarian cancer		^195^
1,7-Bis(4-hydroxyphenyl)-1,4-heptadien-3-one (EB30)	PI3K/Akt pathway↓	Induce apoptosis	A549 (IC_50_ = 8.61 μM), NCI-H292 (IC_50_ = 12.71 μM)	Lung cancer		^196^
Apigenin 7-O-glucoside (AGL)	PTEN/PI3K/Akt pathway↓	Induce apoptosis	HeLa (IC_50_ = 47.26 μM)	Cervical cancer		^197^
C644-0303	WNT/β-catenin↓	Induce apoptosis	HCT‐116‐Luc (IC_50_ = 17.69 μM)	Colorectal cancer		^199^
Obatoclax	survivin↓ WNT/β-catenin↓	Induce apoptosis	HCT 116 (IC_50_ = 89.96 ± 1.68 nM) DLD-1 (IC_50_ = 257.19 ± 1.46 nM)	Colorectal cancer		^200^
CYT997	JAK2/STAT3↓	Induce apoptosis	SGC-7901 (IC_50_ = 70.35 nM), MKN45 (IC_50_ = 77.92 nM) AGS (IC_50_ = 62.74 nM) BGC-823 (IC_50_ = 67.34 nM)	Gastric cancer		^202^
Licochalconce H	JAK2/STAT3↓	Induce apoptosis	HN22 (IC_50_ = 11.8 μM) HSC4 (IC_50_ = 14.4 μM)	Oral squamous cell carcinoma		^203^
AH057	JAK1/2↓	Induce apoptosis	HeLa (IC_50_ = 0.62 μM) CaSki (IC_50_ = 11.28 μM) SiHa (IC_50_ = 17.53 μM)	Cervical cancer		^204^

↓ decrease/inhibition, ↑ increase/activation

### Autophagy-dependent cell death signaling pathways in cancer

**Fig. 3:**
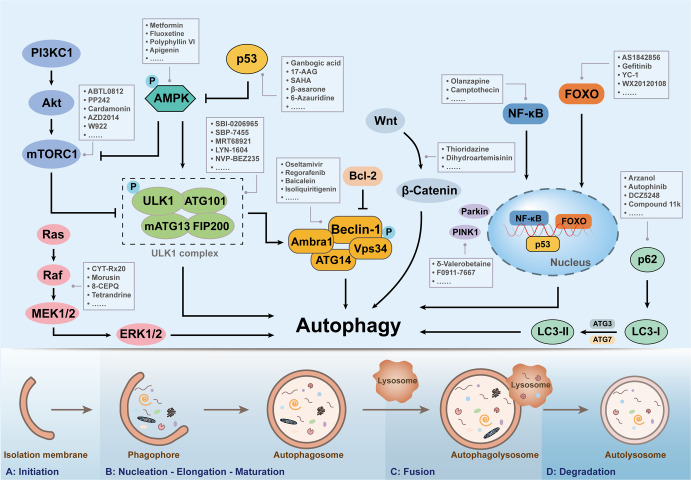
Small-molecule compounds targeting autophagy-dependent cell death pathways in cancer. Autophagy is a complex process regulated by multiple signaling pathways. ULK1 complex is essential during the early-stage initiation of autophagy. ULK1 could be phosphorylated by mTOR or AMPK, which promotes the binding of Beclin-1 to vacuolar protein sorting 34 (VPS34) and ultimately participates in the regulation of autophagy. Autophagy-related signaling pathways, including the Ras/Raf/MEK/ERK pathway, PI3KC1/Akt/mTORC1 pathway, and NF-κB pathway, are significant to autophagy signal transduction and mediate the occurrence of autophagic cell death. p53 is a tumor suppressor protein. Nuclear p53 stimulates autophagy through translocation activation, and cytoplasmic p53 represses autophagy. FoxO regulates autophagy by transcriptional dependent mechanism. p62 is also a key regulator of autophagy that can directly bind to LC3 to promote the formation of autophagosomes

Autophagy is a process of degrading aging and damaged organelles, recycling degradation products, and renewing organelles.^205^ Autophagy plays a dual role in the occurrence and development of tumors. In the early stage of tumor occurrence, autophagy exerts a preventive effect in controlling or killing cancer cells, while in the formed tumor cells, autophagy could maintain the survival of cancer cells and promote development.^205,206^ In the following section, we will focus on the autophagy-related signaling cascade, namely, ULK1 complex, PI3KC1-Akt-mTORC1, Ras-Raf-MAPKs, p53, p62, FoxO, NF-κB, Beclin-1, etc. Meanwhile, we have discussed the research progress of the interaction between small molecule compounds and related signaling pathways in cancer treatment (Fig. 3).

#### Targeting ULK1 complex

ULK1 is the only serine/threonine (S/T) kinases in mammals, as well as a core component of the autophagy pathway.^207^ ULK1 could form a protein complex with focal adhesion kinase interacting protein of 200 KD (FIP200), autophagy-related protein 13 (ATG13), and ATG101, which plays a crucial role in autophagy induction. ULK1 is associated with early autophagosome formation and activates downstream Beclin-1 protein to initiate the autophagy cascade.^208,209^ As a potential multifunctional target, ULK1 plays different roles according to the types and stages of tumors. Both inhibition and activation of ULK1 show significant effects on tumor treatment. For example, blocking early autophagy by inhibiting ULK1 contributed to attenuating cell growth and overcoming the development of drug resistance in tumor therapy, while the activation of ULK1 could mediate the poor prognosis of tumors.^210^ At present, several small-molecule compounds that regulate the ULK1 have been extensively employed in cancer therapy (Table 7).

**Table 7:** Compounds targeting ULK1 and PI3KC1/Akt/mTORC1 in cancer

Compound name and structure	Target	Mechanism in RCD	Cancer cell line (activity)	Tumor type	Clinical trial identifier	Ref.
SBI-0206965	ULK1↓	Inhibit autophagy-dependent cell death	ULK1 (IC_50_ = 108 nM), ULK2 (IC_50_ = 711 nM)	Non-small cell lung cancer		^211^
SBP-7455	ULK1/2↓	Inhibit autophagy-dependent cell death	ULK1 (IC_50_ = 13 nM)	Triple negative breast cancer		^490^
MRT68921	ULK1↓	Inhibit autophagy-dependent cell death	ULK1 (IC_50_ = 2.9 nM), ULK2 (IC_50_ = 1.1 nM)	Lung cancer, colorectal cancer, glioma		^213^
Compound 3s	ULK1↓	Inhibit autophagy-dependent cell death	A549 (IC_50_ = 1.94 ± 2.35 μM)	Non-small cell lung cancer		^214^
Rocaglamide (RocA)	ULK1↓	Inhibit autophagy-dependent cell death	H460 H1975 A549	Non-small cell lung cancer		^215^
Phloretin (PH)	mTOR/ULK1↓	Inhibit autophagy-dependent cell death	MCF7 MDA-MB-231 (IC_50_ = 100 μM)	Breast cancer		^216^
LYN-1604	ULK1↑	Induce autophagy-dependent cell death	ULK1 (EC_50_ = 18.94 nM)	Triple negative breast cancer		^219^
NVP-BEZ235	AMPK/ULK1↑	Induce autophagy-dependent cell death	HCT116 SW48	Colon cancer		^220^
Disulfiram (DSF)	ULK1↑	Induce autophagy-dependent cell death	HT29 (IC_50_ = 0.3 μM), RKO (IC_50_ = 0.25 μM)	Colorectal cancer		^221^
ABTL0812	Akt/mTORC1↓	Induce autophagy-dependent cell death	A549 MiaPaCa-2	Lung cancer, pancreatic cancer	NCT03417921 (phase 1/2)	^227^
PP242	mTORC1↓	Induce autophagy-dependent cell death	HeLa cells	Cervical cancer		^228^
Cardamonin	mTORC1↓	Induce autophagy-dependent cell death	SKOV3 (IC_50_ = 15 μM)	Ovarian cancer		^229^
N-(1-benzyl-3,5-dimethyl-1H-pyrazol-4-yl)benzamide	mTORC1↓	Induce autophagy-dependent cell death	MIA PaCa-2 (EC_50_ = 0.62 μM)	Pancreatic cancer		^230^
Ginsenoside Rg5	PI3K/Akt/mTORC1↓	Induce autophagy-dependent cell death	MG-63 HOS U2OS (EC = 160–1280 nM)	Human osteosarcoma		^231^
AZD2014	mTOR↓	Induce autophagy-dependent cell death	8505C	Thyroid undifferentiated carcinoma		^232^
W922	PI3K/Akt/mTOR↓	Induce autophagy-dependent cell death	HCT116 (IC_50_ = 0.1 ± 1 μM)	Colorectal cancer		^233^
Compound 7j	PI3K/Akt/mTOR↓ ULK1↑	Induce autophagy-dependent cell death	BEL-7402 (IC_50_ = 5.77 ± 0.59 μM) MGC-803 (IC_50_ = 7.21 ± 0.44 μM) T24 (IC_50_ = 3.97 ± 0.23 μM)	Bladder cancer		^234^
Tanshinone IIA	PI3K/Akt/mTOR↓	Induce autophagy-dependent cell death	MCF-7 (IC_50_ = 1–4 μM)	Breast cancer		^235^
20(*S*)-Ginsenoside Rh2	PI3K/Akt/mTOR↓	Induce autophagy-dependent cell death	Reh Jurkat	Acute lymphoblastic leukemia		^236^

↓ decrease/inhibition, ↑ increase/activation

SBI-0206965 is a highly selective ULK1 inhibitor (ULK1: IC_50_ = 108 nM; ULK2: IC_50_ = 711 nM), which could downregulate the ULK1-mediated phosphorylation to inhibit autophagy and survival of NSCLC cells.^211^ Moreover, SBI-0206965 also inhibits the growth of neuroblastoma cell lines and promotes apoptosis by targeting the autophagic kinase ULK1.^212^ Subsequently, the group reported another ULK1/2 dual inhibitor, SBP-7455, with higher activity than SBI-0206965, which could inhibit survival and proliferation of MDA-MB-468 cells via the inhibition of starvation-induced autophagic flux.216 MRT68921 is a dual NUAK family SNF1-like kinase 1 (NUAK1)/ULK1 inhibitor with a strong anticancer effect, which could block ULK1-dependent protective autophagy.^213^

Recently, Sun et al. designed and synthesized a series of new ULK1 inhibitors based on the structure of previously reported ULK1 inhibitors. Among them, compound 3s, 5-bromo-4-(2-fluoro-4-nitrophenoxy)-N-(3,4,5-trimethoxyphenyl) pyrimidin-2-amine showed potent anticancer activity and blocked A549 cell autophagy by inhibiting ULK1 kinase activity, which was accompanied by increasing the expression of p62 and decreasing the level of beclin-1.^214^ Moreover, some natural compounds were shown potent anticancer activity via targeting ULK1. For example, rocaglamide (RocA), a natural product, was reported to inhibit autophagy in NSCLC cells, thus enhancing the sensitivity of NSCLC cells to natural killer (NK) cell-mediated killing. This effect was induced by the suppression of ULK1.^215^ Phloretin (PH) effectively inhibited cytoprotective autophagy via the downregulation of the mTOR/ULK1 pathway, further restoring the sensitivity of breast cancer cells to chemotherapeutic drugs.^216^ Additionally, the research showed that bromodomain and extraterminal domain (BET) inhibitor, JQ1, was considered a hopeful epigenetic agent for the treatment of various tumor types. Studies reported that pro-survival autophagy could cause drug resistance of stem cells to BET inhibitors, and inhibition of autophagy by targeting AMPK-ULK1 could improve the sensitivity of drug-resistant cells to therapeutic drugs (BET inhibitors).^217^

Several types of research have shown that ULK1 was under-expressed in some tumor tissues like breast cancer. Therefore, it suggested that the activation of ULK1 to induce autophagy and thus inhibit tumor growth could be an effective treatment strategy for some cancers.^209,218^ Zhang et al. found a ULK1 activator, LYN-1604, through silico screening and chemical synthesis, which targeted ULK1 and interacted with activating transcription factor 3 (ATF3), RAD21, and caspase 3 to induce autophagy-dependent cell death. Besides, LYN-1604 activated ULK1 with a median effective concentration (EC_50_) value of 18.94 nm in an in vitro kinase assay.^219^ Liu et al. found that a dual PI3K/mTOR, NVP-BEZ235, promoted autophagy colon cancer cell death via the upregulation of the AMPK/ULK1 pathway.^220^ Disulfiram (DSF) was an anti-alcohol drug that could suppress malignant tumor growth. Hu et al. reported that DSF combined with Cu could significantly inhibit the viability of CRC cells and trigger autophagic cell death instead of apoptosis via enhancing the ULK1 level.^221^

#### Targeting PI3KC1/Akt/mTORC1

PI3KC1/Akt/mTORC1 signaling pathway has been considered as the pivotal regulator in various cell processes. PI3K, a class of lipid kinases, could be divided into three types, including PI3KC1, PI3KC2, and PI3KC3; among them, PI3KC1 is the most widely studied.^222^ mTOR is a highly conserved serine/threonine (Ser/Thr) kinases, which belong to the PI3K protein kinase family and is a downstream effector protein of the PI3K/Akt signaling pathway. mTOR exists as a form of two complexes, mTORC1 and mTORC2, of which mTORC1 is related to nutrients and growth factors and is an essential negative regulator of autophagy.^223–225^ This pathway regulates autophagy by phosphorylating various substrates downstream of mTORC1, such as ULK1 complex and beclin-1.^222^ Targeting PI3KC1/Akt/mTORC1-mediated autophagy was a promising therapeutic strategy for multiple tumors and exerted essential role in suppressing cell growth and improving the chemosensitivity of tumor cells.^226^ Numerous small-molecule compounds target PI3KC1/Akt/mTORC1-mediated autophagy, leading to tumor cell death.

ABTL0812, a novel first-in-class compound, was found to inhibit the Akt/mTORC1 pathway and induce autophagy-dependent cancer cell death by high-throughput silicon screening comparison. This mechanism was mediated by activating the expression of the Tribbles-3 pseudokinase (TRIB3) gene.^227^ ABTL0812 was in clinical evaluation in phase 1/1b trial in advanced cancer patients (NCT02201823). PP242 was an mTOR inhibitor with stronger pro-autophagy activity than rapamycin, which could activate lysosomal by the blockade of the mTORC1 level.^228^ In ovarian cancer SKOV3 cells, cardamonin was able to induce autophagy via the inhibition of mTORC1, and the downregulation of Raptor was involved.^229^ Additionally, the synthesized compound, N-(1-benzyl-3,5-dimethyl-1H-pyrazol-4-yl)benzamide, could prevent the reactivation of mTORC1 and increase the accumulation of LC3-II to promote Induce autophagy-mediated cell death.^230^ Inhibition of the PI3K/Akt/mTOR pathway could promote tumor cell apoptosis by increasing the level of autophagy. The natural product, ginsenoside Rg5, was reported to trigger human osteosarcoma cell apoptosis via the LC3-mediated autophagy pathway by inhibiting the activation of PI3K/Akt/mTORC1.^231^

In addition to the small molecules mentioned above, more small molecules showed potent antitumor activity via the regulation of autophagy. For example, AZD2014, as a dual mTOR inhibitor, could suppress proliferation and induce autophagy to sensitize thyroid undifferentiated carcinoma (ATC) cells to paclitaxel (PTX).^232^ Another novel PI3K/Akt/mTOR inhibitor, W922, was shown to attenuate the growth of CRC cells via the induction of autophagy and when treated in combination with chloroquine, caused a large production of apoptotic cells.^233^ Chen et al. synthesized a quinazolinyl-arylurea derivative, compound 7j, which possessed a lower IC_50_ value and good selectivity. Compound 7j regulated PI3K/Akt/mTOR/ULK1 and Sxc(-)/GPX4/ROS pathways to induce autophagy and ferroptosis of cancer cells at high concentrations while inducing apoptosis at low concentrations.^234^ It had been reported that some active substances of traditional Chinese medicine had strong antitumor effects, such as Tanshinone IIA (Tan IIA), 20(S)-ginsenoside Rh2 (20 (S)-GRh2), could induce cancer cell cycle arrest and autophagy by inhibiting PI3K/Akt/mTOR pathway, accompanied by increased expression of LC3-II protein^235,236^ (Table 7).

#### Targeting Ras/Raf/MAPKs

MAPK pathway is the main pathway for transmitting signals from the cell membrane to the nucleus, which could be divided into three subtypes: ERK1/2, JNK, and p38 MAPK.^237^ Among them, ERK1/2 plays a critical role in autophagic regulation in various tumor cells, and ERK1/2 could be phosphorylated and activated to regulate autophagy through the Ras/Raf/MEK/ERK axis.^12,238^ The Ras/Raf/MEK/ERK pathway was closely associated with tumorigenesis. Therefore, small-molecule compounds induce autophagy-dependent cell death by the Ras/Raf/MAPKs pathway may be a potential therapeutic strategy for cancer.

For example, a small molecule 2′-dihydroxy-4,4′-dimethoxydihydrochalcone had been reported to induce autophagy-dependent cell death in MKN45 cells via activating ROS/MEK/ERK pathway, as well as upregulating Beclin-1, Atg5 and, Atg7 levels.^239^ CYT-Rx20, a synthetic β-nitrostyrene derivative, could induce autophagy which was regulated by the MEK/ERK pathway.^240^ In A549 and NCI–H292 cells, morusin triggered the inhibition of Akt and activation of JNK and ERK pathways to induce autophagy-mediated cell death and apoptosis.^241^ 8-CEPQ was shown to attenuate the growth of colon cancer cells via triggering autophagy-dependent cell death by the activation of ERK.^242^ Furthermore, the administration of tetrandrine at the maximum noncytotoxic dose of 50 mg/kg could induce autophagy of nasopharyngeal carcinoma cells by inhibiting the MEK/ERK pathway and enhancing the sensitivity of nasopharyngeal carcinoma to radiotherapy without side effects occurs^243^ (Table 8).

**Table 8:** Compounds targeting Ras/Raf/MAPKs, p53, p62, FoxO, and NF-κB in cancer

Compound name and structure	Target	Mechanism in RCD	Cancer cell line (activity)	Tumor type	Clinical trial identifier	Ref.
2′-dihydroxy-4,4′-dimethoxydihydrochalcone	ROS/MEK/ERK↑	Induce autophagy-dependent cell death	MKN45 (IC_50_ = 8 μM)	Gastric cancer		^239^
CYT-Rx20	MEK/ERK↑	Induce autophagy-dependent cell death	MDA-MB-231 (IC_50_ = 1.82 ± 0.05 μM), MCF-7 (IC_50_ = 0.81 ± 0.04 μM), ZR75-1 (IC_50_ = 1.12 ± 0.06 μM),	Breast cancer		^240^
Morusin	Akt↓ JNK, ERK↑	Induce autophagy-dependent cell death	A549 (IC_50_ = 12.32 μM), NCI–H292 (IC_50_ = 7.92 μM)	Lung cancer		^241^
8-C-(E-phenylethenyl) quercetin (8-CEPQ)	ERK↑	Induce autophagy-dependent cell death	SW620 (IC_50_ = 20 μM), HCT116 (IC_50_ = 15 μM)	Colon cancer		^242^
Tetrandrine	MEK/ERK↓	Induce autophagy-dependent cell death	CNE1 CNE2 C666-1	Nasopharyngeal carcinoma		^243^
Gambogic acid	Mutant p53↓	Induce autophagy-dependent cell death	MDA-MB-231 DLD1	Triple negative breast cancer, colorectal cancer		^245^
17-AAG	Mutant p53↓	Induce autophagy-dependent cell death	OCI-AML3 NB4	Acute myeloid leukemia	NCT00079404 (phase 1)	^247^
Suberoylanilide hydroxamic acid (SAHA)	Mutant p53↓	Induce autophagy-dependent cell death	MDA-MB-231 DLD1	Triple negative breast cancer, colorectal cancer	NCT00126451 (phase 2)	^248^
*Trans*-chalcone	p53↑ β-catenin↓	Induce autophagy-dependent cell death	HuH7.5 (IC_50_ = 23.66 μM)	Hepatocellular Carcinoma		^249^
β-asarone	p53↑	Induce autophagy-dependent cell death	U251 (IC50 = 720 μM)	Glioma		^250^
6-Azauridine	p53↑	Induce autophagy-dependent cell death	HCT116	Colorectal cancer		^251^
[4-NH2-2-Me(Q)H][VO(bcma)(H2O)]2H2O (T1)	p53, p21↑	Induce autophagy-dependent cell death	PANC-1 (IC_50_ = 44.67 μM), MIA PaCa2 (IC_50_ = 72.22 μM), hTERT-HPNE (IC_50_ = 140.9 μM)	Pancreatic cancer		^252^
FH535+ AZD5363	p53↑	Induce autophagy-dependent cell death	HepG2 Hep3B	Hepatocellular Carcinoma		^253^
Arzanol	p62↑	Inhibit autophagy-dependent cell death	RT-112 (IC_50_ = 6.6 μM)	Bladder cancer		^257^
Autophinib	p62↑	Inhibit autophagy-dependent cell death	VPS34 (IC_50_ = 19 nM)	Breast cancer		^258^
DCZ5248	p62↑	Inhibit autophagy-dependent cell death	HCT116 (IC_50_ = 283.9 ± 64.1 nM), LS174T (IC_50_ = 107.7 ± 17.6 nM), HT-29 (IC_50_ = 111.1 ± 4.9 nM)	Colon cancer		^259^
Tetrahydroquinolin-2(1H)-one derivative 11k	p62↑	Inhibit autophagy-dependent cell death	PANC-1 (IC_50_ = 4.9 μM)	Pancreatic cancer		^260^
7-aminobenzo[cd]indol- 2(1H)-one 33	p62↑	Inhibit autophagy-dependent cell death	HT-29 (IC_50_ = 12 μM)	Colorectal adenocarcinoma		^261^
AS1842856	FOXO1↓	Inhibit autophagy-dependent cell death	HCT116 HepG2	Colon cancer, hepatocellular carcinoma		^264,266^
Gefitinib+YC-1	FOXO1↓	Inhibit autophagy-dependent cell death	NCI-H1975 (GEF: IC_50_ = 11.56 ± 2.93 μM; YC-1: IC_50_ = 23.04 ± 5.99 μM), NCI-H1944 (GEF: IC_50_ = 16.82 ± 2.10 μM; YC-1: IC_50_ = 7.74 ± 4.51 μM)	Non-small cell lung cancer		^267^
WX20120108	FOXO3↑	Induce autophagy-dependent cell death	HeLa (IC_50_ = 12.72 ± 4.46 μM) MDA-MB-231 (IC_50_ = 14.37 ± 1.49 μM)	Cervical cancer, triple negative breast cancer		^268^
Olanzapine	NF-κB/p65↓	Induce autophagy-dependent cell death	T98 LN229 U87 (IC_50_ = 100–200 μM)	Glioblastoma		^271^
Camptothecin	NF-κB/AMPK/mTOR/ULK1↓	Induce autophagy-dependent cell death	EC1 EC109	Esophageal cancer	NCT01612546 (phase 2)	^272^

↓ decrease/inhibition, ↑ increase/activation

#### Targeting p53

p53 protein is well-established as a tumor suppressor gene as well as an essential regulator of autophagy. As a nuclear transcription factor, p53 could activate the transcription of autophagy-related genes, thus inducing autophagy, while p53 in the cytoplasm has a negative regulatory role in inhibiting the occurrence of autophagy. p53 protein is involved in the initiation of autophagy. Unlike p53-induced apoptosis, many p53 target genes can stimulate autophagy, and this effect is usually achieved by inhibiting the mTOR signaling axis, a negative regulator of autophagy. Besides, p53 can also activate the expression of various autophagy-related proteins such as Atg7 and Atg10. Most cancer patients have mutations in the p53 gene, and the remaining possess defective wild-type p53 pathways.^244^ Accordingly, we primarily focus on inhibiting the expression of mutant p53 protein and restoring the function of wild-type p53 to retard further tumor development through small molecule compounds so as to achieve the goal of effective treatment of cancer.

Accordingly, blocking the expression of mutant p53 protein leads to inhibiting cell growth and proliferation, reducing the resistance to some anticancer agents as well as improving the poor prognosis.^244^ For example, small molecule gambogic acid (GA) was shown to trigger the degradation of mutant p53 and thus enhance the sensitivity of cancer cells to the chemotherapeutic agent.^245^ In cancer cells, the high expression of some proteins, such as heat shock protein 90 (HSP90), enhanced the stability of p53 mutant protein, while hsp90 inhibitors could reduce this stability and degrade mutant p53.^246^ Like Hsp90 inhibitor, 17-AAG, promoted mutant p53-R248Q degradation by stimulating autophagy.^247^ Similarly, suberoylanilide hydroxamic acid (SAHA), as an HDAC inhibitor, could induce autophagy and prevent the expression of mutant p53 protein.^248^

On the other hand, p53 protein presented a vital role in tumorigenic inhibition, and it was feasible to induce cell death by repairing the wild-type p53 pathway. *Trans*-chalcone (TC) was a stable isomer with various properties, such as antioxidant, anti-proliferation, and anti-inflammation. Treatment with TC could activate the expression of p53 gene and downregulate the β-catenin protein, thus inducing the autophagy death of hepatocellular carcinoma (HCC) cells.^249^ β-asarone activated the expression of p53 protein and p53 pathway-related proteins such as Beclin-1, AMPK, and LC3-II/I to induce autophagic cell death.^250^ Similarly, 6-Azauridine (6-AZA)-mediated autophagy death depended on the expression of p53 and AMPK.^251^ [4-NH2-2-Me(Q)H][VO(bcma)(H2O)]2H2O (T1) suppress proliferation and induced autophagy which had been shown to activate p53/p21 pathway.^252^ Furthermore, the combination of β-catenin inhibitor FH535 and Akt inhibitor AZD5363 could stimulate the expression and phosphorylation of p53 protein in the nucleus, further regulating the AMPK/mTOR/ULK1 pathway, ultimately inducing autophagy-dependent death of HCC cells^253^ (Table 8).

#### Targeting p62, FoxO, and NF-κB

p62, also known as sequestosome 1 (SQSTM1), is a ubiquitin-binding protein involved in cellular signal transduction as well as a selective substrate of autophagy.^254^ p62 directly interacts with LC3 through the LC3-interacting region (LIR) within p62 to promote the formation of autophagosomes, while the inhibition of autophagy is accompanied by insufficient degradation of the p62 protein.^255^ Therefore, p62 and LC3 are widely established as markers of autophagy flux in cancer research. p62 is regarded as a potential target for monitoring autophagy, and some small molecules could influence the expression level of p62, thus regulating autophagic cell death.^256^ Arzanol, as a novel compound, was observed to induce the accumulation of p62 and inhibit autophagy, thus improving the sensitivity of bladder cancer RT-112 cells to cisplatin.^257^ Autophinib, as a VPS34 inhibitor, could inhibit p62 degradation via suppression of starvation- or Rapamycin-triggered autophagy by targeting VPS34 in MCF7-LC3 cells.^258^ A dual inhibitor of both Hsp90 and late-autophagy, DCZ5248, promoted the LC3-II transformation and upregulated p62 level to inhibit colon cancer HCT116 cell late-autophagy.^259^ Furthermore, Shen et al. designed and synthesized a tetrahydroquinolin-2(1H)-one derivative 11k with an IC_50_ value of 4.9 μM in PANC-1 cell, which could enhance the expression of p62 to inhibit autophagy.^260^ 7-aminobenzo[cd]indol- 2(1H)-one 33 as a novel Atg4B inhibitor was shown to inhibit autophagy via decreasing the degradation of p62, as well compound 33 could sensitize HT-29 cell to oxaliplatin.^261^

In mammals, the FoxO family includes FoxO1, FoxO3, FoxO3, and FoxO6, which act as transcription factors by binding to target DNA through the forkhead domain to activate or inhibit downstream genes, thus affecting the occurrence and development of cancer.^262^ FoxO proteins could participate in the cell autophagy process to regulate cancer growth and metastasis. FoxO-autophagy regulation plays a different role according to the tumor environment, which can promote or inhibit tumor progression.^263^ Accordingly, FoxO is also modulated by various signaling pathways, including acetylation, ubiquitination, and phosphorylation.

FoxO-mediated autophagy exhibited promising therapeutic potential in regulating cancer progression. The study found that histone deacetylase inhibitors (HDACIs) inhibited the mTOR expression by activating FoxO1 and SESN3, thereby activating autophagy and inducing drug resistance.^264^ When combined with autophagy inhibitors, autophagy could be inhibited, thus inhibiting drug resistance and enhancing the therapeutic effect.^265^ Xiao et al. also reported that HDACIs induced autophagy via the AMPK-FoxO1-ULK1-Snail axis in hepatoma cells and combination with FoxO1 inhibitor, AS1842856, was more effective in the treatment with HCC.^266^ When treating NSCLC with gefitinib, the FOXO1 protein expression would increase and induce protective autophagy, thus leading to drug resistance in NSCLC cells. The combination of gefitinib and a HIF-1α inhibitor, YC-1, could significantly inhibit autophagy, as well as acquired resistance could be overcome.^267^ WX20120108, as a novel IAP inhibitor, triggered autophagy in HeLa and MDA-MB-231 cells via upregulating the FOXO3 gene and promoting ROS production.^268^ Furthermore, in an acidic microenvironment, FOXO3 could trigger autophagy by enhancing the expression of LC3-II and Beclin-1 to inhibit tumor cell growth.^269^

NF-κB as a nuclear transcription factor was overexpressed in multiple malignancies and promoted tumor cell survival. Small-molecule compounds activated autophagy via the blocking of the NF-κB pathway to exert an antitumor biologic effect.^270^ Studies conducted by Zhu et al. demonstrated that olanzapine-induced autophagy via the suppression of the NF-κB pathway also had an inhibitory effect on the MGMT-positive gene, thus leading to the inhibition of proliferation.^271^ Camptothecin (CPT) was shown to promote esophageal cancer cells’ protective autophagy via the suppression of neddylation, the accumulation of IκBα, and the blockade of the NF-κB pathway. Besides, CPT could also inhibit the AMPK/mTOR/ULK1 axis from regulating autophagy^272^ (Table 8).

#### Targeting Beclin-1

Beclin-1, a crucial autophagy regulatory gene in mammals, is homologous to yeast autophagy-related gene Atg6/Vps30. Beclin-1 is an indispensable protein for the activation of autophagy and is controlled by different transcription factors.^273^ Beclin-1 has a BH3 binding domain, which interacted with Vps34 and promoted the formation of PI3KCIII core complexes, thereby regulating autophagy.^274^ Beclin-1 is a critical tumor suppressor that participates in the process of tumor development by regulating autophagy activity.^275^ It was found that Beclin-1 was under-expressed in some malignant tumors, such as lung cancer, breast cancer, cervical cancer, ovarian cancer, etc. Therefore, Beclin-1 has been considered a potential therapeutic target for tumor therapy.

Oseltamivir, an anti-influenza virus drug, was shown to increase Beclin-1 expression and decrease p62 expression to trigger autophagy-dependent death of Huh-7 cells.^276^ Moreover, regorafenib was reported to trigger autophagy-dependent cell death via blocking the interaction of Beclin-1-Bcl-2 and further promoting the dissociation of Beclin-1 from Bcl-2.^277^ 4-Methoxydalbergione (4MOD) had been recently reported to upregulate Beclin-1 and LC3-II/LC3-I, inducing autophagy-dependent cell death and thus inhibiting the growth of J82 and UMUC cells, which accompanied with the suppression of Akt/ERK pathway.^278^ Interestingly, natural products, BA and isoliquiritigenin (ISL) were shown to stimulate autophagic cell death in ovarian cancer cells via the activation of Beclin-1^279,280^ (Table 9).

**Table 9:** Compounds targeting Beclin-1 and other targets of autophagy in cancer

Compound name	Target	Mechanism in RCD	Cancer cell line (activity)	Indication of tumor type	Clinical trial identifier	Ref.
Oseltamivir	Beclin 1↑ p62↓	Induce autophagy-dependent cell death	Huh-7 HepG2	Liver cancer		^276^
Regorafenib	Beclin 1↑	Induce autophagy-dependent cell death	U251 (IC_50_ = 11.98 μM) U87 (IC_50_ = 17.48 μM) H4 (IC_50_ = 5.04 μM) U118 (IC_50_ = 11.51 μM)	Glioblastoma multiforme		^277^
4-Methoxydalbergione (4MOD)	Beclin 1↑	Induce autophagy-dependent cell death	J82 (IC_50_ = 8.17 ± 1.91 μM), UMUC3 (IC_50_ = 14.5 ± 0.92 μM)	Bladder cancer		^278^
Baicalein (BA)	Beclin 1↑	Induce autophagy-dependent cell death	HEY cell	Ovarian cancer		^279^
Isoliquiritigenin	Beclin 1↑	Induce autophagy-dependent cell death	OVCAR5 ES-2 (IC_50_ = 10–20 μM)	Ovarian cancer		^280^
Metformin	AMPK↑ mTORC1/mTORC2↓	Induce autophagy-dependent cell death	RPMI8226 (IC_50_ = 20.2 ± 1.2 mM), U266 (IC_50_ = 17.9 ± 1.1 mM)	Multiple myeloma	NCT02948283 (phase 1)	^281^
Fluoxetine	AMPK↑	Induce autophagy-dependent cell death	MDA-MB-231 MDA-MB-436 (IC_50_ = 0.3 ± 0.7 μM)	Triple negative breast cancer		^282^
Polyphyllin VI (PPVI)	PI3K/Akt/mTOR↓ AMPK↑	Induce autophagy-dependent cell death	A549 (IC_50_ = 3.114 ± 0.072 μM) H1299 (IC_50_ = 3.713 ± 0.323 μM)	Non-small cell lung cancer		^284^
Apigenin (APG)	AMPK↑	Induce autophagy-dependent cell death	AGS SNU-638	Gastric cancer		^285^
Docosahexaenoic acid (DHA)	AMPKα/Raptor↑ mTOR/ULK1↓	Induce autophagy-dependent cell death	MCF-7	Breast cancer	NCT01282580 (phase 1)	^286^
δ-Valerobetaine	PINK1/Parkin/LC3B↑	Induce mitophagy	SW480 SW620 (IC_50_ = 1.5 mM)	Colon cancer		^287^
F0911-7667	AMPK-mTOR-ULK; SIRT1-PINK1-Parkin↑	Induce autophagy-dependent cell death; Induce mitophagy	U87MG T98G	Glioblastoma		^288^
Thioridazine (THD)	Wnt/β-catenin↓ p62↑	Induce autophagy-dependent cell death	GBM8401 (IC_50_ = 18.2 ± 1.3 μM), U87MG (IC_50_ = 12.4 ± 1.1 μM)	Glioblastoma		^289^
Dihydroartemisinin (DHA)	Wnt/β-catenin↓	Induce autophagy-dependent cell death	CAG (IC_50_ = 1–5 μM) JJN3 (IC_50_ = 5–10 μM) RPMI8226 (IC_50_ = 0.4–0.5 μM)	Multiple myeloma		^22^

↓ decrease/inhibition, ↑ increase/activation

#### Other targets

AMPK, as a critical cell energy sensor, has been proved to regulate the development of tumors through autophagy. AMPK can directly activate ULK1 and thus stimulate autophagy via the phosphorylation of Ser317 and Ser777. In addition, AMPK inhibits the phosphorylation of downstream mTOR to promote the expression of ULK1, further initiating autophagy. Some small-molecule compounds were used to target AMPK for cancer treatment. In multiple myeloma, metformin was shown to induce autophagy and cell cycle arrest via the dual repression of mTORC1 and mTORC2 pathways mediated through AMPK activation.^281^ Fluoxetine was reported to cause autophagic cell death via activating the AMPK-mTOR-ULK1 axis in TNBC cell lines.^282,283^ Moreover, polyphyllin VI (PPVI) was a natural product with potent antitumor activity, which could regulate multiple pathways to induce autophagic cell death, such as PI3K/Akt/mTOR, MEK/ERK, and AMPK/ mTOR pathways.^284^ Apigenin (APG) elicited autophagic cell death by modulating the mTOR-AMPK-ULK1 signaling pathway in GC cell lines.^285^ In addition, Docosahexaenoic acid (DHA) was shown to increase the accumulation of oxidative stress-induced growth inhibitor 1 (OSGIN1) and ROS to activate AMPKα/Raptor and inhibit mTOR/ULK1 pathways in MCF-7 cells, leading to the formation of autophagosome.^286^

PTEN-induced putative kinase 1 (PINK1)/Parkin pathway is an important pathway to mediate mitochondrial autophagy (Mitophagy). Parkin is an E3-ubiquitin ligase that, when activated, drives mitochondrial proteins’ ubiquitination to induce autophagy. δ-Valerobetaine (δVB) as a dietary metabolite was reported to activate mitophagy in SW480 and SW620 colon cancer cells via PINK1/Parkin/LC3B axis.^287^ F0911-7667, a sirtuin-1 (SIRT1) activator, could also induce mitophagy via the SIRT1–PINK1–Parkin pathway in U87MG and T98G cells, and induce autophagic cell death through regulating the AMPK-mTOR-ULK pathway.^288^ Additionally, thioridazine (THD), an antipsychotic drug, was used to enhance p62-mediated autophagy and apoptosis of glioblastoma multiform (GBM) cells by regulating the Wnt/β-catenin pathway^289^ (Table 9).

### Necroptosis signaling pathways in cancer

**Fig. 4:**
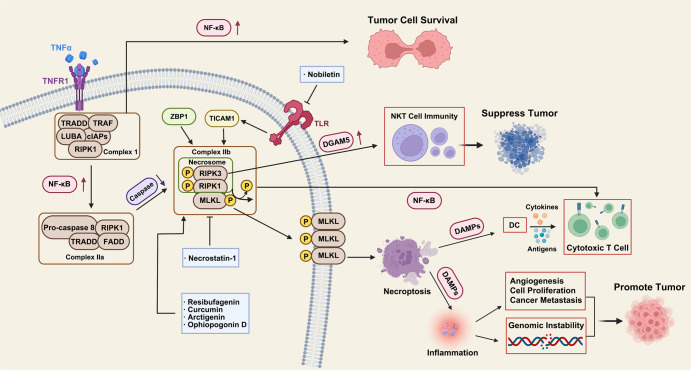
Small-molecule compounds targeting necroptosis pathways in cancer. There are three main pathways to fight tumor by targeting necroptosis. In TNF-α After binding with TNFR1 on the plasma membrane, the downstream protein molecules TRADD, TRAF, cIAPs, LUBA and RIPK1 are recruited to form complex I and activate NF-κB to promote the survival of tumor cells. Then RIPK1 promotes the recruitment of pro-caspase-8 to produce activated Caspase-8 and form complex IIa. If caspase-8 is inhibited or there is no expression of caspase-8, RIP3 is recruited to form rip1-rip3 necrosome, causing ripk3 phosphorylation, MLKL is recruited to form complex IIb and induce necroptosis. In addition, TLR ligand can also mediate RIPK3-MLKL dependent necrosis through TICAM1. ZBP1 acts upstream of RIPK3 and interacts with RIPK3 through its RHIM domain to mediate necroptosis in response to viral infection

Universally, the occurrence of cancer is closely related to cell death. In addition to apoptosis, necrotic apoptosis is also related to the development of cancer. However, studies have shown that necroptosis plays a dual role in cancer progression and development.^290^ Necroptosis is a regulatory form of cell death, which is different from apoptosis, autophagy and necrosis by releasing inflammatory mediators as a mechanism to defend against viruses. However, the potential molecular mechanism of necroptosis is complex and has not been fully elucidated. Some studies believe that RIPK1 has no kinase activity in complex I. inhibiting RIPK1 activity by necrostatin-1 has no effect on TNF-induced NF-κB signaling pathway, but it can prevent the formation of complex IIB from inhibiting necroptosis.^291,292^ Therefore, the role of RIPK1 in cells can determine whether cancer cells survive or undergo necroptosis through targeted drugs.^292^ RIPK3 activation of MLKL is a key regulatory pathway in necroptosis. The upstream inducer DR, Toll-like receptor (TLR), or virus induces the activation of RIPK3 through RIPK1, toll-like receptor adaptor molecule 1 (TICAM1), and Z-DNA–binding protein 1 (ZBP1), respectively. Additionally, the adhesion receptor (AR) activates RIPK3 via an unknown adaptor protein or kinase.^293^ Necroptosis plays a dual role in the occurrence and development of tumor cells. On the one hand, necroptosis can inhibit the proliferation, migration, and invasion of tumor cells. On the other hand, necroptosis may be involved in the growth of early tumors. Recent studies suggest that tumor cells resistant to apoptosis may be sensitive to the necroptosis pathway,^294,295^ suggesting that the study of tumor cells’ necroptosis and its regulatory mechanism is expected to become a target for the treatment of tumors (Fig. 4 and Table 10).

**Table 10:** Compounds targeting RIPK1/ RIPK3/MLKL, TLR4/TICAM1, ZBP1 and other targets of necroptosis in cancer

Compound name and structure	Target	Mechanism in RCD	Cancer cell line (activity)	Indication of tumor type	Ref.
necrostatin-1 (NEC-1)	RIPK1/RIPK3↓ and JNK/c-Jun↓	Inhibit necroptosis	HT-29 (100 μM)	Colitis associated cancer	^302^
Resibufagenin	RIPK3/ PYGL/ GLUL/ GLUDl↑	Induce necroptosis	HCT116 (IC_50_ = 5 μM)	Colorectal cancer	^303^
Reactivation of transcriptional reporter activity (RETRA)	p21↑, cyclin-D3↓, ROS↑	Induce necroptosis	C-33A (IC_50_ = 50 μM, 96 h) SiHa (IC_50_ = 60 μM, 96 h; IC_50_ = 90 μM, 72 h)	Cervical cancer	^308^
NP-ALT	tyrosine phosphorylation of p27kip1 (CDKN1B) ↓, ROS↑, CDK4↓, CDK2↓	Induce necroptosis	MCF7 ESRY537S	Breast cancer	^309^
Curcumin	cleaved caspase-3, cleaved PARP, p-RIP3 and p-MLKL↑, TLR4/TICAM1↓	Induce necroptosis	PC-3AcT MCF-7 (IC_50_ = 25 μM, 48 h; IC_50_ = 25–50 μM, 24 h) MDA-MB-231 (IC_50_ = 50 μM, 24 h; (IC_50_ = 5–10 μM, 48 h)	Prostate cancer	^310^
Arctigenin	ROS, CCN1, p-RIP3 and p-MLKL↑	Induce necroptosis	PC3 PC3AcT (IC_50_ = 40 μM, 24 h; IC_50_ = 20 μM, 48 h; IC_50_ = 10 μM, 72 h)	Prostate cancer	^311^
Ophiopogonin D’ (OPD’)	RIPK1↑	Induce necroptosis	LNCaP (IC_50_ = 5.34 μM, 24 h)	Prostate cancer	^312^
Nobiletin (NOB)	TLR4/ TICAM1/IRF3↓, TLR9/IRF7↓	Inhibit necroptosis	PC3 LNCaP (IC_50_ = 20 μM, 48 h)	Prostate cancer	^323^
Shikonin	Avoided the apoptosis resistance mediated by p-glycoprotein, Bcl-2 and Bcl XL	Induce necroptosis	MCF-7 (IC_50_ = 5 μM)	Breast cancer	^332^

↓ decrease/inhibition, ↑ increase/activation

#### Targeting RIPK1/ RIPK3/MLKL

It is generally believed that the dysfunction of necroptosis is related to the occurrence and development of tumors. For example, RIP3 expression is significantly downregulated in patients with AML, an invasive hematopoietic malignancy known to block hematopoietic differentiation and cell death. The decrease of RIP3 reduces the death of hematopoietic cells, which is related to the occurrence of AML.^296^ Another study reported that the genetic defect of RIP3 transformed flt3-itd and runxeto-driven mouse bone marrow proliferation into AML by increasing the accumulation of leukemia-initiating cells.^297^ Therefore, the role of RIP3 in the pathogenesis of AML may be determined by the cellular environment. Furthermore, low expression of MLKL is associated with reduced overall survival in patients with colon cancer after surgery.^298^ A study analyzed that RIPK1 protein and mRNA levels were significantly upregulated in tumor samples from 40 patients with colorectal adenocarcinoma, while ripk3 and p-MLKL decreased in colorectal cancer, suggesting that necroptosis may be reduced and apoptosis may be increased in tumor cells.^299^ Similarly, studies have found that the expression of RIPK3 in colorectal cancer tissues is significantly lower than that in adjacent normal tissues, and the upregulation of RIPK3 can inhibit the proliferation, migration, and invasion of colorectal cancer cells.^300^ Interestingly, RIPK3 was found to be highly expressed in tumors in a mouse inflammatory bowel cancer (CAC) model, and further analysis of colon cancer tissue chips from 168 tumors and 103 non-tumor controls found that RIPK3 was highly expressed in human colorectal cancers. However, after knocking out the RIPK3 gene in mice, the number and size of tumors were significantly reduced, and the tumor load was also reduced. RIPK3 may be involved in early tumor growth through enteritis mouse model experiments.^300^ MLKL is also downregulated in pancreatic and cervical squamous cell carcinomas, where low levels of MLKL in plasma predict poor prognosis in pancreatic and ovarian cancers.^301^ Altogether, this information provides research directions for studying necrotic proteins in tumor development. Based on the above research results, current research believes that necroptosis is a double-edged sword, and the role of RIPK1/RIPK3/MLKL in various cancer tissues still needs to be confirmed by multi-center, prospective clinical controlled experiments.

Studies have shown that traditional necroptosis inducers or existing chemotherapeutic agents can necrotize many cancer cell lines. These cell lines cover almost all common cancer types, especially colorectal cancer cells and hematopoietic system tumors, which are more sensitive to necroptosis inducers.^294^ Necrostatin-1 (NEC-1) is a specific inhibitor of necroptosis by preventing the interaction between ripk1 and ripk3, which can specifically inhibit necroptosis without affecting normal cell function and apoptosis. A study studied the antitumor effect of NEC-1 through colitis associated cancer (CAC) mouse model and considered that NEC-1 can significantly inhibit tumor growth and development by inhibiting JNK/c-Jun signal pathway.^302^ At the same time, several necrostatin related compounds with necroptosis inhibitory activity, including NEC-3, NEC-4, NEC-5, NEC-7 and so on, have been gradually studied in depth. Screening necroptosis inducers is also a new strategy for drug resistance of tumor cells. Resibufagenin induces CRC cell necroptosis by inducing RIPK3 mediated activation of glycogen phosphorylase (PYGL), glutamine synthetase (GLUL), and glutamate dehydrogenase (GLUDL) so as to inhibit tumor growth.^303^ It shows that RIPK3 is not only a molecular switch of necrotic cells but also a hub to control the metabolic state of cells.^304,305^ Some complexes have been proved to induce necrotic apoptosis of tumor cells and play an antitumor role, but they are still limited to basic research. The polypeptide Su-X targeting survivin-XIAP complex can induce necrotic apoptosis of tumor cells. The necrotic apoptosis-related proteins (p-RIPK1, p-RIP3, and p-MLKL) in the cells treated with Su-X are significantly increased, suggesting that Su-X can inhibit the development of tumors by inducing necrotic apoptosis of tumor cells.^306^ Apoptosis inducers are largely related to cancer drug resistance. Drugs that kill tumor cells through non-apoptotic pathways can bypass the traditional drug resistance. RIPK1 and RIPK3 are the key molecules of cell death and survival pathway and important potential targets for tumor treatment.^307^ Reactivation of transcriptional reporter activity (RETRA) is a small molecule that can induce the expression of the p53 regulatory gene in the mutant (MT) p53 cells. It shows a necrotic form through the phosphorylation of RIPK1/RIPK3/MLKL so that the cervical cancer cell cycle stagnates in the S phase, p21 is upregulated, cyclin-d3 is downregulated, mitochondrial hyperpolarization increases the production of ROS, and finally selectively induces cervical cancer cell necroptosis regardless of p53 state, and it has no cytotoxic effect on normal human peripheral blood mononuclear cells (PBMC).^308^ The combined application of RETRA and necroptosis inhibitor Necrostatin-1 reversed the effect of RETRA and saved the death of cervical cancer cells.^308^ It is suggested that RETRA-induced necrosis and apoptosis can be used as a potential treatment for apoptosis-resistant cervical cancer. In the clinical use of cyclin D-CDK4 inhibitor (CDK4I) in HR+ breast cancer, the problem of CDK4 inhibitor resistance is usually caused by compensatory CDK2 activity.^309^ A therapeutic liposome peptide NP-ALT inhibits the tyrosine phosphorylation of p27kip1 (CDKN1B) and the activity of CDK4 and CDK2 by inducing ROS and RIPK1 dependent necroptosis in breast cancer cells and xenotransplantation models resistant to endocrine therapy. It provides a new treatment option for HR+ tumors resistant to endocrine and CDK4 inhibitors.^309^ In recent years, traditional Chinese medicine has had obvious curative effects in the treatment and remission of cancer, and there are relatively few adverse reactions. Major bioactive curcumin derived from the rhizome of *Curcuma longa* induces necroptosis and apoptosis by increasing cleaved caspase-3 and cleaved PARP, p-RIP3, and p-MLKL proteins and finally reduces the viability of tolerant prostate cancer pc-3act cells.^310^ Arctigenin, a mitochondrial complex I inhibitor, induces necroptosis in prostate cancer cells through ROS-mediated mitochondrial damage and increased CCN1 levels, ultimately increasing p-RIP3 and p-MLKL levels. Pretreatment with the necroptosis inhibitor necrostatin-1 restored their levels and prostate cancer cell viability.^311^ Ophiopogonin D’ (OPD’), a natural compound extracted from *Ophiopon japonicus*, induces significant necroptosis in androgen-dependent LNCaP cancer cells by activating and increasing Fas ligand (FasL)-dependent RIPK1 protein expression and exerts antitumor effects.^312^

In addition, the combination of FMRP protein with RIPK1 mRNA suggests that FMRP regulates the necroptosis pathway by monitoring the metabolism of RIPK1 mRNA. The use of FMR1 anti transcription therapy in CRC cell lines will upregulate RIPK1 and cause necrotic apoptosis of CRC cells.^313^ In conclusion, the susceptibility to necrotic apoptosis inducers may be of great significance for the clinical treatment of colorectal tumors. Interestingly, radiotherapy is one of the main methods of treating cancer. More than 50% of tumor patients receive radiotherapy during their disease treatment, of which 40% can be cured by radiotherapy.^314^ However, tumor recurrence is still one of the key factors of treatment failure.^315^ Radiation-induced necroptosis results in the formation of RIPK1/RIP3/MLKL necrotic bodies by upregulating the phosphorylation of RIPK1 and RIPK3.^316^ The blocking of necroptosis regulatory genes, especially MLKL, by low-dose chemical inhibitors or gene deletion can significantly inhibit the recurrence of tumors in and out of mice, and even reduce the tumorigenicity of mice.^316^ Detecting the increase of IL-8 in colorectal cancer cells after irradiation, puts forward a new way - RIPK1/RIP3/MLKL/JNK/IL-8 is involved in tumor re proliferation mediated by necroptosis cells,^316^ MLKL/JNK/IL-8 may become a potential target to block tumor regrowth and improve the efficacy of radiotherapy. This study suggests that radiotherapy for necrotic apoptosis may be an effective way to improve the effect of radiotherapy.

#### Targeting TLR4/TICAM1

Although RIPK3 and MLKL appear to be common players in necroptosis, but RIPK1 is not involved in TLRs-mediated necroptosis.^317^ TLRs are commonly expressed in innate immune cells and tumor cells.^318^ TLR ligands mediate RIPK3-MLKL-dependent necrosis through TICAM1(also known as TRIF). Interestingly, TLR4 has both tumor-promoting and tumor-suppressive effects,^318^ and targeting TLR4 activation or inhibition may be a potential therapeutic strategy for different types of tumors. Many studies have shown that the gene expression profile of peripheral blood mononuclear cells (PBMC) in blood samples that are more specific and more convenient to collect clinically shows unique gene expression characteristics in several cancers.^319^ A significant and unique gene expression feature was found in breast cancer patients,^320^ which showed the possibility of dividing breast cancer patients into subgroups.^320^ Sporadic thyroid cancer is the most common endocrine malignancy in which the risk of papillary thyroid cancer (PTC) is associated with single nucleotide polymorphisms (SNPs). One study showed that the TICAM1 (rs8120) gene in PTC was associated with SNPs.^321^ In addition, there was a significant interaction between TICAM1 (rs8120) and FOXE1 (rs10984377), a known susceptibility locus for thyroid cancer, suggesting that multiple RCD mechanisms and host factors may interact in complex ways to increase the risk of PTC and FTC.^321^ TLR4 is overexpressed in breast tumors, thereby promoting tumor progression and metastasis. The natural product Curcumin inhibits the survival of breast cancer cells by inhibiting the TLR4-dependent TICAM1 signaling pathway, reducing the level of interferon (IFN-α/β).^322^ Nobiletin (NOB), an O-methylated flavonoid, inhibits the growth of different types of prostate cancer cells to varying degrees by inhibiting TLR4/TICAM1/IRF3 and TLR9/IRF7 signaling pathways, depending on hormonal status and aggressiveness characteristics.^323^

#### Targeting ZBP1

As an interferon-induced z-nucleic acid sensor, ZBP1, also known as DAI/DLM-1, acts upstream of RIPK3 and interacts with RIPK3 through its Rhim (RIP homotype interaction motif) domain to mediate necrosis and apoptosis in response to viral infection or TLR signal transduction.^324^ The N-terminal domain (ND) of ZBP1 is important for the ZBP1-ZBP1 homologous interaction, and the RHIM domain of its C-terminal region interacts with RIPK3, triggering RIPK3-dependent necroptosis.^325^ Notably, the virus can not only directly bind to RIPK3 but also promote the binding of host protein ZBP1 to RIPK3, ultimately activating MLKL.^322^ Interestingly, in irradiated tumor cells, zbp1-mlkl necrotic cascade induces cytoplasmic DNA accumulation and then autonomously activates CGAs sting signal to drive persistent inflammation. Resection of caspase-8 can enhance the activation of the sting pathway and the antitumor effect of radiation by activating mlkl and improve the alternative radiotherapy strategy.^326^ Metastasis involves separating tumor cells from primary tumors and acquiring migration and invasion ability. These abilities are mediated by various events, including the loss of intercellular contact, the increase of adhesive transfer, and the inability to maintain normal cell polarity. Inhibition of ZBP1-mediated necroptosis promotes tumor growth in mouse colorectal cancer and melanoma models.^327^ Other similar studies have proved that the loss of ZBP1 function relieves the regulation of many mRNAs involved in cell movement and cell cycle regulation, resulting in phenotypic changes in breast cancer, which not only increases the growth capacity of metastatic cells but also promotes cell migration.^328^ In T47D and MDA231 human breast cancer cells, targeting imp1/zbp1 regulates the local expression of many cell movement-related mRNAs, such as those encoding E-cadherin, α/β- actin, and arp2/3 complex, so as to stabilize intercellular junction and focal adhesion and inhibit tumor cell invasion.^329^ Interestingly, another study had shown that the expression of ZBP1 was significantly increased in necrotic tumors. ZBP1 is a key regulator of tumor necrosis and apoptosis. Its deletion blocks tumor necrosis and apoptosis during tumor development and inhibits tumor metastasis in MVT-1 breast cancer model, providing a potential drug target for controlling tumor metastasis.^199^

#### Other targets

Cell FLICE (FADD-like IL-1β-converting enzyme) inhibitor protein (c-FLIP) is not only a major anti-apoptotic protein but also an important cytokine and chemoresistance factor that inhibits cytokine and chemotherapy-induced apoptosis. It has been reported that the expression level of c-FLIP is elevated in colorectal cancer,^330^ and the c-FLIP isomer is involved in switching apoptosis and necrotic cell death.^331^ The c-FLIP isomer in ribosomes determines whether cell death occurs ripk3 mediated bad apoptosis or caspase-dependent apoptosis. The defect of apoptosis signal transduction and the upregulation of drug transporters in cancer cells produce clinical drug resistance by significantly limiting the effectiveness of cancer chemotherapy. Overexpression of p-glycoprotein, Bcl-2 or Bcl-xL may be the main cause of clinical tumor drug resistance. Interestingly, drugs that induce non-apoptotic cell death can overcome cancer drug resistance. Shikonin, a naturally occurring naphthoquinone sensitive breast cancer cell line, and drug-resistant cell line, showed the same necroptosis, which proved that shikonin avoided the apoptosis resistance mediated by p-glycoprotein, Bcl-2 and Bcl-xL by inducing necroptosis of drug-resistant cancer cell lines, thereby inhibiting drug resistance.^332^

As a newly discovered mode of programmed death, necroptosis is closely related to the physiological process of many cases. At present, the research on necroptosis is mostly in the basic experimental stage. Necroptosis plays an opposite role in antitumor. On the one hand, it can inhibit the proliferation and migration of tumor cells; On the other hand, it can promote tumor growth and participate in early tumor formation. Further study on the molecular mechanism of necrotic apoptosis pathway and the relationship between upstream and downstream signal molecules of related signal pathways, exploring its role in different tumor modes and finding corresponding targeted drugs are one of the directions to improve the effect of tumor treatment in the future.

### Pyroptosis signaling pathways in cancer

**Fig. 5:**
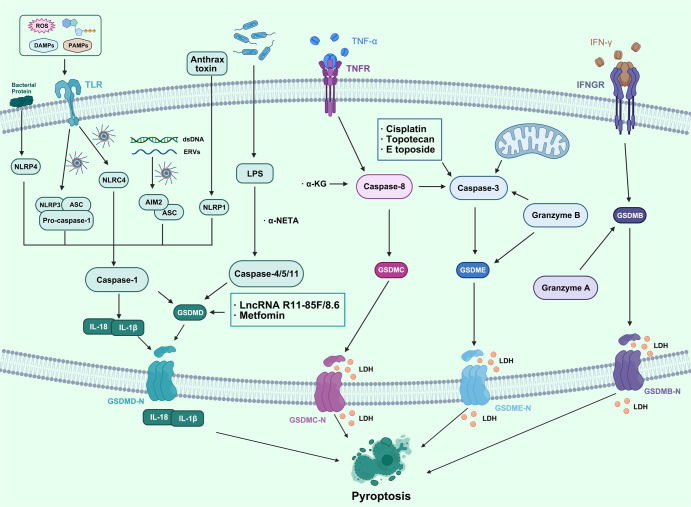
Small-molecule compounds targeting pyroptosis pathways in cancer. There are two main pathways and two other pathways that exert antitumor activity by targeting pyroptosis. Pyroptosis pathway can be divided into classical pyroptosis pathway and non-classical pyroptosis pathway. The activation of classical pyroptosis pathway is initiated by PAMPs or damps. After NLRs or ALRs recognize specific stimuli, they start to assemble to form inflammatory bodies and process to form activated caspase-1. Caspase-1 cuts GSDMD, and the N-terminal of GSDMD is located and aggregated on the cell membrane to form pores. In addition, caspase-1 cleaves pro-IL-1β and pro-IL-18 to form mature IL-1β and IL-18, and the intracellular contents are secreted outside the membrane through the membrane pore. The nonclassical focal death pathway depends on the activation of caspase-4/Caspase-5/caspase-11. After stimulated by LPS in the cytoplasm, caspase-4/Caspase-5/caspase11 can directly bind to the conserved structure lipid A of LPS, cause oligomerization and activation, further cut GSDMD, cause the N-terminal of GSDMD to disintegrate and locate in the cell membrane to form membrane pores. In caspase-3-dependent cell death, GSDME is the reaction substrate of Caspase-3. GSDME can be cleaved by activated caspase-3 to generate its N-terminal fragment, which performs pyroptosis by penetrating the plasma membrane. Granzyme B can also participate in NK cell induced pyroptosis by cleaving GSDME. In addition, TNF-γ acts on TNFR, activates the N-terminal cleavage of GSDMC mediated by caspase-8, locates in the cell membrane, forms membrane pores, and finally induces pyroptosis. IFN-γ by acting on ifngr, causes the N-terminal of GSDMB to cleave and locate to the cell membrane to form membrane pores and induce pyroptosis. Granzyme A can also induce pyroptosis by cleaving GSDMB

Pyroptosis is an inflammatory regulated cell death mediated by GSDM, which is mainly characterized by membrane perforation, cell swelling, cell content overflow, chromatin condensation, and DNA breakage.^333,334^ The human GSDM family includes six members, including GSDM-A, -B, -C, -D, -E, and DFNB59; all GSDM family members have N-terminal pore-forming domain, C-terminal self inhibitory domain, and ring domain connecting N-terminal and C-terminal domain. Among them, GSDMD and GSDME are the most complete to study the mechanism of inducing cell death. During the formation of the tumor and tumor microenvironment, pyroptosis has the dual effects of inhibiting and promoting its formation:^335^ on the one hand, inflammatory bodies released during pyroptosis can inhibit the proliferation and metastasis of tumor cells, and its mechanism is that NLRP3 inflammatory bodies produced by pyroptosis inhibit tumorigenesis by secreting inflammatory cytokines; On the other hand, the aggregation of inflammatory bodies contributes to the formation of tumor microenvironment and promotes tumor occurrence and development. Studies have shown that NLRP3 inflammasome plays an important role in the aggregation of myeloid-derived suppressor cells (MDSC) and the inhibition of antitumor T cell immune response after DC immunization.^336^ NLRP3 in tumor-associated macrophages drives the polarization of immunosuppressive CD4+ T cells in the tumor immune microenvironment of pancreatic ductal adenocarcinoma through IL-1.^337^ In addition, the production of IL-22 depends on the activation of NLRP3 inflammatory bodies and the subsequent release of IL-1 from immune cells. IL-22 is closely related to the development of a variety of tumors, such as lung cancer, skin cancer, breast cancer, and gastric cancer.^338^ A bioorthogonal chemistry system (BCS) selectively releases active GSDM to tumor cells. However, only 10–30% of tumor cells undergo pyroptosis to completely remove tumor grafts, and there is no tumor regression in immunodeficient mice.^339^ Therefore, how to use the new weapon of pyroptosis to develop new tumor treatment schemes, reduce the drug resistance of chemotherapy drugs and enhance the body’s immunity has become an urgent problem to be solved (Fig. 5 and Table 11).

**Table 11:** Compounds targeting Caspase-1/-4/-5/-11/GSDMD, Caspase-3/GSDME and other targets of pyroptosis in cancer

Compound name and structure	Target	Mechanism in RCD	Cancer cell line (activity)	Indication of tumor type	Ref.
Metformin	GSDMD ↑	Induce pyroptosis	KYSE510 KYSE140	Esophageal squamous cell carcinoma	^189^
2-(alpha-naphthoyl) ethyltrimethylammonium Iodide (α-NETA)	Caspase-4/GSDMD ↑	Induce pyroptosis	Ho8910 (IC_50_ = 16.94 μM)	Epithelial ovarian cancer	^345^
LncRNA RP1-85F18.6	ΔNp63 ↓, GSDMD ↑	Induce pyroptosis	SW480 SW620 HCT116	Colorectal cancer	^347^
Topotecan	Caspase-3/GSDME ↑	Induce pyroptosis	SH-SY5Y	Glioma	^350^
Etoposide	Caspase-3/GSDME ↑	Induce pyroptosis	SH-SY5Y	Glioma	^350^
Cisplatin	Caspase-3/GSDME ↑	Induce pyroptosis	SH-SY5Y	Glioma	^350^
Irinotecan	Caspase-3/GSDME ↑	Induce pyroptosis	SH-SY5Y	Glioma	^350^
Doxorubicin (DOX)	Caspase-3/GSDME ↑	Induce pyroptosis	HeLa	Cervical cancer	^350^
5-fluorouracil (5-FU)	Caspase-3/GSDME ↑	Induce pyroptosis	HeLa	Cervical cancer	^350^
3′,5′-diprenylated chalcone (C10)	Caspase-3/GSDME ↑	Induce pyroptosis	PC3 (IC_50_ = 4.56 ± 0.493 μM, 24 h) DU145 (IC_50_ = 7.33 ± 0.769 μM, 24 h) RWPE-1 (IC_50_ = 7.10 ± 0.682 μM, 24 h)	Prostate cancer	^360^
Dihydroartemisinin (DHA)	AIM2/caspase-3/DFNA5↑	Induce pyroptosis	MCF7 (IC_50_ = 100 μM, 36 h) MDA-MB-231 (IC_50_ = 50 μM, 24 h)	Breast cancer	^361^
α-ketoglutarate (α-KG)	ROS/ DR6↑, caspase-8/GSDMC ↑	Induce pyroptosis	HeLa	Lung cancer	^81^

↓ decrease/inhibition, ↑ increase/activation

#### Targeting Caspase-1/-4/-5/-11/GSDMD

Caspase-1-dependent cell pyroptosis, also known as the classical inflammatory corpuscle pathway, is based on the activation of inflammatory corpuscles. Caspase-4/-5/-11 dependent cell pyroptosis has nothing to do with the inflammatory corpuscle complex, so this pathway is also known as the non-classical inflammatory corpuscle pathway. There is a close connection between pyroptosis and apoptosis. Knockdown of GSDMD can block IL-1β secretion and convert pyroptosis into apoptosis, thereby promoting tumor cell killing.^340^ It has been studied to genetically engineer GSDMD by inserting other protease sites or Caspase-3/-7 cleavage sites between the N-terminal and C-terminal domains of GSDMD, which can also convert apoptosis into pyroptosis.^341^ Interestingly, the expression of GSDMD in gastric cancer cells was lower than that in adjacent normal tissue cells, which may promote the proliferation of cancer cells. GSDMD reduces the expression of Cyclin A2 and Cyclin-Dependent Kinase (CDK2) by inhibiting ERK1/2, STAT3, and PI3K/Akt in gastric cancer (GC) cells. Therefore, the decrease of GSDMD expression in GC cells increases the expression of the Cyclin/CDK complex as a substance that regulates the cell cycle, promotes the transition from the S phase to the G2 phase, and accelerates the proliferation of GC cells.^342^ However, in NSCLC, the expression level of GSDMD in tumor tissues is significantly higher than that in adjacent tissues and normal tissues.^343^ Silencing GSDMD in NSCLC cells or using GSDMD inhibitors can inhibit the progression of NSCLC to a certain extent, which indicates that the high expression of GSDMD may promote the progression of NSCLC.^343^ Studies have shown that treatment of endometrial cancer cell lines Ishikawa and HEC1A with the hydrogen-rich medium in vitro can upregulate the release of inflammatory mediator IL-1β and promote cell pyroptosis.^344^ The volume and weight of the tumor were significantly reduced compared with the untreated group when water was administered to the mice transplanted with endometrial cancer tumors by gavage.^344^ After the GSDMD gene was knocked out in endometrial cancer cells, and the above results showed no significant difference between the hydrogen-rich and non-hydrogen-rich groups.^344^ This indicates that the pyroptosis of endometrial cancer cells may be achieved through hydrogen-induced GSDMD-mediated IL-1β secretion, so hydrogen-rich-induced pyroptosis of endometrial cancer cells may become a new therapeutic method.

Due to the resistance to apoptosis, chemotherapy as the main treatment of advanced human esophageal squamous cell carcinoma (ESCC) has little effect. The upregulation of proline, glutamate, and leucine-rich protein-1 (PELP1) in advanced ESCC is highly correlated with cancer progression and poor prognosis. Metformin or pyroptosis inducer activates mir-497 by targeting the mir-497/PELP1 axis, inhibits PELP1 expression, increases the level of cleaved GSDMD, induces pyroptosis in ESCC, and improves the prognosis of ESCC.^189^ A new antitumor molecule 2-(alpha-naphthoyl) ethyltrimethylammonium Iodide (α-NETA) induces pyroptosis in different epithelial ovarian cancer (EOC) cell lines through GSDMD/ caspase-4 pathway and reduces the size of EOC tumor in mice. Knockout of caspase-4 or GSDMD seriously hindered the killing activity of α- NETA on EOC cells.^345^ Accumulating evidence suggests that abnormal expression of lncRNAs may regulate cancer cell proliferation and metastasis.^346^ LncRNA RP1-85F18.6 is highly expressed in colorectal cancer and can regulate ΔNp63 at the transcriptional and translational levels (its role is opposite to that of p53), thereby participating in the proliferation, invasion, survival, and metastasis of colorectal cancer cells, including inhibiting the apoptosis of tumor cells.^347^ It is worth noting that LncRNA RP1-85F18.6 is also involved in the pyroptosis process of colorectal cancer cells. Downregulation of LncRNA RP1-85F18.6 induces pyroptosis in CRC cells by silencing ΔNp63 and cleaving GSDMD.^347^ Studies have found that lncRNA GAS5 can promote inflammasome assembly and expression by interfering with glucocorticoid receptor expression, thereby upregulating the expression of IL-1β and IL18 inflammatory mediators mediated by caspase-1, promoting tumor cell pyroptosis, and promoting tumor cell pyroptosis.^348^ In both cell and animal experiments, ovarian tumor cell growth and migration were inhibited in a time-dependent manner; when lncRNA GAS5 was knocked out, as its expression decreased, the pyroptotic effect was weakened, and the tumor-inhibiting effect was also weakened.^348^ The above results indicate that lncRNA GAS5 can inhibit tumor progression by promoting the pyroptosis of ovarian cancer cells, and the reduction of GAS5 expression can lead to the occurrence of ovarian cancer.

#### Targeting Caspase-3/GSDME

In Caspase-3-dependent pyroptosis, GSDME is the reaction substrate of Caspase-3, and GSDME is involved in the regulation of secondary necrosis.^349,350^ Under the stimulation of apoptosis, GSDME can be cleaved by activated caspase-3 to generate its N-terminal fragment (gsdme NT), which performs pyroptosis by penetrating the plasma membrane.^349,350^ Granzyme is an exogenous serine protease released by cytotoxic T lymphocyte (CTL) and natural killer (NK) cells. After entering the target cells, it induces the apoptosis of the target cells by activating the apoptosis-related enzyme system. The human body contains five kinds of granzymes, namely granzymes A, B, H, K, and M. Granzyme participates in NK cell induced pyroptosis by cleaving GSDME.^351^ There is a two-way relationship between pyroptosis and tumor treatment. Activating chronic inflammation can promote tumor development while activating acute pyroptosis will lead to necrotic pyroptosis so as to inhibit tumor progression and achieve the effect of antitumor treatment. Some research results show that the pyroptosis caused by hypoxia in the tumor center can promote the development of tumor and reduce the survival rate of patients.^352^ Chronic inflammation caused by inflammatory mediators increases the risk of tumorigenesis. Epithelial pyroptosis releases HMGB1, which can promote the occurrence of colitis-related colorectal cancer by activating the ERK1/2 pathway.^353^ On the other hand, as inflammatory pyroptosis, pyroptosis can activate the immune system. Immune stimulants, including HMGB1, can induce the activation of dendritic cells and antitumor T cells. Tumor-infiltrating immune cells can induce pyroptosis of tumor cells.^354^ Chimeric antigen receptor gene-modified T (car-t) cells can induce GSDME-dependent pyroptosis in leukemia cells, and its mechanism involves the cleavage of activated GSDME in leukemia cells by granzyme B released by car-t cells. It is worth noting that the activity of car-t cells to induce target pyroptosis is determined by the number of perforin/granzyme B in car-t cells, rather than the number of perforin/granzyme B in existing CD8+ T cells.^354^ In addition, granzyme A has also been shown to induce pyroptosis of tumor cells by cutting GSDMB.^355^ The lysed GSDMB was introduced into mouse tumor cells, and the tumor could be effectively controlled by immune checkpoint therapy.^355^ The expression of GSDMB is induced by IFN - and the combination of IFN - and immune checkpoint blocking can greatly activate antitumor immunity. Therefore, the Granzyme family plays an important role in CTL-induced pyroptosis. The activation of pyroptosis in CTL can enhance cytotoxicity. At present, immune checkpoint inhibitors (ICI) have broad application prospects in clinical tumor treatment, but relevant data show that only 1/3 of patients respond to ICI.^356^ There is a synergistic effect between cell focal death and ICI. Inducing target cell focal death will promote anti-ICI tumors to obtain sensitivity to ICI.^339^ The mechanism is that the rupture of inflammatory cell membrane promotes the overflow of cell contents, triggers a strong inflammatory response and a large number of lymphocyte infiltration, and these significantly increased lymphocytes further induce caspase-3-dependent tumor pyroptosis, forming positive feedback to improve the antitumor effect.^357^ A special chimeric costimulatory converting receptor (CCCR), which is composed of the extracellular region of PD-1, the transmembrane and cytoplasmic region of NKG2D, and the cytoplasmic region of 41BB.^358^ CCCR modified NK92 cells showed enhanced activity against human lung cancer H1299 cells in vitro by extensively inducing pyroptosis. However, another study suggested that the inhibitory effect of antigen-specific CTL on the tumor was not related to pyroptosis.^359^ Therefore, pyrosis is a kind of pyroptosis in the form of immune stimulation, which can cooperate with ICI to improve the effect of immunotherapy.

One study found that GSDME was highly expressed in SH-SY5Y neuroblastoma, HeLa cervical cancer cells, and MeWo skin melanoma cells.^350^ GSDME positive SH-SY5Y cells showed pyroptosis characteristics after treatment with chemotherapeutic drugs topotecan, etoposide, cisplatin, or Irinotecan.^350^ After GSDME positive HeLa cells were treated with DOX or 5-fluorouracil (5-FU), the originally induced apoptosis was switched to CASP3 dependent pyroptosis.^350^ 3′,5′-degraded chalcone (C10) activates caspase-3 by inducing PKCδ/JNK pathway, induces PARP and GSDME-dependent pyroptosis, increases the proportion of sub G1 PC3 cells, and selectively inhibits the proliferation of prostate cancer cells in vitro and in vivo.^360^ Dihydroartemisinin (DHA), a derivative of artemisinin extracted from the traditional Chinese medicine *Artemisia annua*, can increase the expression of natural dermal protein E (DFNA5) and melanoma 2 (AIM2) by activating Caspase-3, and finally, induce pyroptosis to inhibit the proliferation and tumorigenicity of breast cancer cells.^361^ Knockout of aim2 and DFNA5 could significantly enhance the resistance of breast cancer cells to DHA.^361^ It reveals the new anti-cancer mechanism of DHA and also brings a promising treatment strategy for breast cancer.

#### Other targets

So far, few studies have focused on other GSDM family members except GSDMD and GSDME. In an acidic environment, α-ketoglutarate (α-KG) is inhibited by MDH1 and transformed into L-2HG, which increases the level of ROS, leads to the oxidation of the death receptor DR6 located in plasma membrane, triggers its endocytosis, recruits caspase-8-mediated GSDMC cleavage, and finally induces pyroptosis.^81^ Treatment with α-KG derivative DMΑ-KG can further improve the level of ROS and make cancer cells that originally resisted pyroptosis more vulnerable to pyroptosis induced by α-KG.^81^ It reveals that GSDMC has potential clinical value in tumor treatment. A study found two new gene association sites (17q12 and 8q24.21) related to the risk of childhood acute lymphoblastic leukemia.^362^ The peak slice size related to acute lymphoblastic leukemia in 17q12 is about 200KB, and the pyrolytic substrate GSDMB is also expressed in this fragment.^362^ At the gene level, GSDMB may affect the site expression of 17q12, thus affecting the risk of acute lymphoblastic leukemia; the relationship between GSDMB as a substrate causing cell death and the pathogenesis, treatment, and prognosis of acute lymphoblastic leukemia needs to be further studied.^362^

### Ferroptosis signaling pathways in cancer

**Fig. 6:**
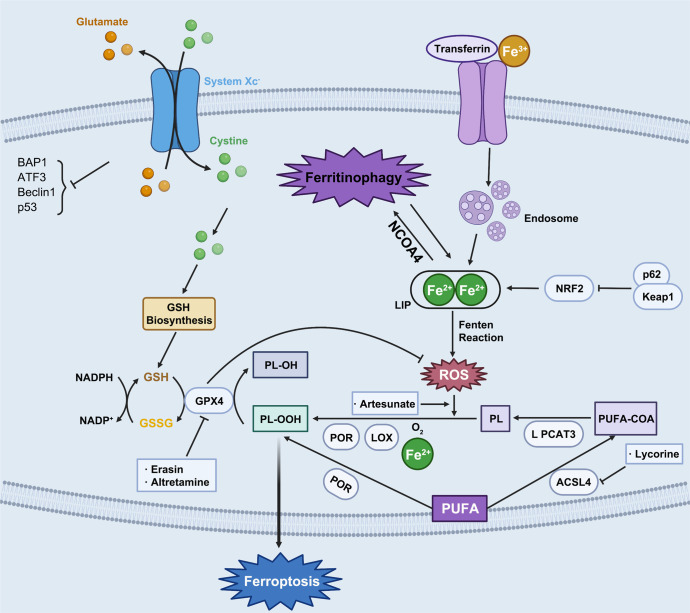
Small-molecule compounds targeting ferroptosis pathways in cancer. There are two main pathways to exert antitumor activity by targeting ferroptosis. The intracellular antioxidant stress system mainly relies on GPX4 to remove excess lipid peroxide. GPX4 of the antioxidant system will reduce the lipid peroxide PL-OOH to the corresponding lipid alcohol PL-OH, so as to reduce the burden of lipid peroxidation and protect the cell membrane from damage. Cystine enters the cytoplasm through SLC7A11, transforms into cystine, and enters the GSH biosynthesis pathway. GSH is involved in the hydrolysis of PL-OOH by GPX4. The inhibition of GSH synthesis or the inactivation of GPX4 can make the excess PL-OOH in cells unable to be cleared, resulting in cell oxidative damage and the occurrence of ferroptosis. It is worth noting that in this process, BAP1, ATF3, Beclin1 and p53 will inhibit the function of SLC7A11. When intracellular iron is overloaded, a large number of free radicals can react with PUFA of cell membrane phospholipids under the catalysis of ester oxygenase and iron, and produce a large number of PL-OOH through the promotion of POR, a positive regulatory protein of iron death phospholipid peroxidation in tumor cells, resulting in ferroptosis. In addition to the direct pathway of PUFA mediated PL-OOH production, PUFA can also be incorporated into phospholipid membrane through ACSL4, esterified into PUFA COA, esterified into PL through LPCAT3, and then oxidized into toxic PL-OOH by lipoxygenases (LOXs). In addition, the extracellular Fe^3+^ is combined with transferrin, transported into the cell through TFR1 and reduced to Fe^2+^, and then stored in the intracellular LIP with the help of intracellular NRF2. Fe^2+^ can transfer electrons to produce free radicals or ROS with oxidation ability through Fenton reaction with peroxide, so as to promote the oxidation process of LOXs

ACSl4, LPCAT3, and ALOXs (especially ALOX15) pathways mediate the oxidation of polyunsaturated fatty acids, including arachidonic acid, which is necessary for the lipotoxicity of ferroptosis. The upregulation of ACSl4 is the sign of ferroptosis.^293^ On the contrary, some antioxidant systems, especially the XC system (including core component SLC7A11), GPX4, NFE2L2, and some heat shock proteins (such as HSPs), inhibit the ferroptosis lipid peroxidation process.^293^ The ultimate goal of clarifying the potential mechanism of ferroptosis is to obtain better cancer treatment options. Therefore, based on the molecular regulation mechanism of ferroptosis, it is worthwhile to specifically target the key regulators of ferroptosis for triggering ferroptosis. Some drugs or compounds have been found to induce ferroptosis of tumor cells, which can be divided into two categories according to the mechanism of action (Fig. 6 and Table 12).

**Table 12:** Compounds targeting ACSL4/LPCAT3/ALOX15, SLC7A11/GPX4/NFE2L2 and other targets of ferroptosis in cancer

Compound name and structure	Target	Mechanism in RCD	Cancer cell line (activity)	Indication of tumor type	Ref.
Artesunate (ART)	Ras/ROS ↑	Induce ferroptosis	BxPC-3 Panc-1	Pancreatic cancer	^373^
Lycorine	GPX4 ↓, ACSL4 ↑	Induce ferroptosis	786-O (IC_50_ = 10 μM, 24 h) A498 (IC_50_ = 20 μM, 24 h) Caki-1 (IC_50_ = 5 μM, 24 h)	Renal cell carcinoma	^278^
Erastin (ERA)	GPX4 ↓	Induce ferroptosis	HT-1080 (GI_50_ = 1.7 mM)	Human fibrosarcoma	^43^
piperazine erastin analysis (PE)	GPX4 ↓	Induce ferroptosis	HT-1080 (GI_50_ = 0.9 mM)	Human fibrosarcoma	^43^
AE	GPX4 ↓	Induce ferroptosis	HT-1080 (GI_50_ = 8 nM)	Human fibrosarcoma	^43^
imidazole ketone erastin (IKE)	GPX4 ↓	Induce ferroptosis	BJeLR (IC_50_ = 3 nM) HT-1080 (GI_50_ = 310 nM)	Human fibrosarcoma	
Altretamine	GPX4 ↓	Induce ferroptosis	U-2932	Diffuse large B-cell lymphoma	^377^
FINO2	Oxidizing iron, GPX4 ↓	Induce ferroptosis	HT-1080 (10 μM)	Human fibrosarcoma	^378^

↓ decrease/inhibition, ↑ increase/activation

#### Targeting ACSL4/LPCAT3/ALOX15

Lipid peroxidation (LP) reflects the process that biofilms, lipids, and other lipid-containing molecules related to PUFA are oxidized by oxides such as ROS to form lipid peroxidate (LPO). Current research shows that the accumulation of PUFA oxide is a sign of ferroptosis, and its accumulation process mainly involves deoxygenation inhibition, mainly referring to GPX4 and ferroptosis suppressor protein 1 (FSP1) and the enhancement of peroxidation catalyzed by iron and a series of enzymes.^363^ Arachidonic acid (AA) and adrenaline (ADA) can be esterified into acyl CoA derivatives by acyl CoA synthetase long-chain family member 4 (ACSl4) and then esterified into phosphatidyl ethanolamine (PE) by recombinant lysophosphatidyl-choline acyltransferase 3 (lpcat3), and then oxidized into toxic lipid hydroperoxides by LOXs. LOXs is an iron-containing enzyme and the most important lipid oxidase in ferroptosis.^364^ Therefore, the activation of ACSl4, lpcat3, and LOXs will lead to excessive lipid peroxidation and ferroptosis. ACSL member ACSl4 is an important contributor to the ferroptosis of tumor cells.^365^ ACSl4 tends to catalyze the conversion of arachidonic acid and adrenic acid in polyunsaturated fatty acids into arachidonyl coenzyme A and adrenoyl coenzyme A, respectively. These products can participate in the synthesis of negatively charged phospholipids such as phosphatidylethanolamine or phosphatidylinositol. Phosphatidylethanolamine is a key substrate for lipid peroxidation during ferroptosis, especially when GPX4 is inhibited. Therefore, ACSl4 gene knockout or functional inhibition can effectively prevent the occurrence of ferroptosis.^8,366^ A number of studies have suggested that ACSl4 can be used as a biomarker to predict whether tumor cells can successfully die of iron.^8,365,367^ ACSl4 mediated ferroptosis can inhibit the proliferation of glioma cells, so it has the potential to become a new target for glioma treatment.^368^ Interestingly, although ACSl4 is indispensable for ferroptosis induced by erastin or (1s, 3R) - rsl3, it cannot determine the final occurrence of p53-alox12 mediated ferroptosis.^369^ Whether this process needs the participation of other members of the ACSL family remains to be studied.

The incurable cancer is called pancreatic ductal adenocarcinoma (PDAC), driven by mutations in constitutively active KRAS.^370,371^ Oncogenic KRAS reprogrammes PDAC cells to a highly anti-apoptotic state.^372^ Resistance to apoptosis makes PDAC highly resistant to the mitochondrial pattern of apoptosis-regulated cell death.^372^ Artesunate (ART), an antimalarial drug, has the highest cytotoxicity in PDAC cell lines with constitutively active KRAS, specifically inducing ROS and lysosomal iron-dependent cell death.^373^ PDAC patients have increased sensitivity to Ras-driven ferroptosis.^373^ Combined treatment with iron ptosis inhibitor ferrostatin-1 can block art-induced lipid peroxidation and cell death and increase the long-term survival and proliferation of PDAC cells.^373^ Lycorine extracted from *Amaryllidaceae general* can reduce the expression level of GPX4, increase the expression level of ACSL4, increase the expression level of 5-HETE, 12-HETE, 15-HETE and MDA, reduce the ratio of GSH/GSSG, induce tumor cells to produce ferroptosis and inhibit the proliferation of renal cell carcinoma (RCC).^278^

#### Targeting SLC7A11/GPX4/NFE2L2

Erastin (ERA) is a typical ferroptosis inducer, which can directly inhibit the cystine/glutamate antiporter system x_c_^−^ on the cell membrane, reducing the transport of extracellular cystine into cells.^40^ The deletion of cystine reduces intracellular glutathione synthesis, which indirectly inhibits downstream GPX4, accelerates the accumulation of lipid peroxides to lethal doses, and induces ferroptosis.^43^ Butionine-sulfoximine can block the synthesis of glutathione by inhibiting the activity of γ-glutamine cysteine synthase and, finally promote ferroptosis.^43^ Various drugs have been found to induce ferroptosis through a mechanism of glutathione depletion. In addition to the indirect inhibition of GPX4 by depletion of glutathione, it can also directly inhibit GPX4 and induce ferroptosis. Tumor cells such as diffuse large B-cell lymphoma and renal cell carcinoma have been found to be very sensitive to ferroptosis regulated by GPX4,^43^ and some drug-resistant tumor cells are also highly dependent on GPX4 to maintain their own survival.^374^ Therefore, GPX4 is a new target for some tumor therapy.

Due to its poor water solubility and effect, ERA hinders its further use in vivo. In order to enhance the water solubility of the scaffold, piperazine erastin analysis (PE) was obtained by introducing piperazine into the middle of the aniline ring of ERA.^43^ PE showed significant activity in the nude mouse tumor prevention model but had limited effect on the determined tumor growth.^43^ In order to improve the effect, AE was obtained by replacing piperazine with aldehyde based on PE, but it was accompanied by poor metabolic stability, and solubility.^43^ In order to achieve the best balance between reactivity, stability and solubility, the active carbonyl was transferred to the scaffold without electrophilic function, and the metabolically stable electrophilic ketone erastin analog imidazole ketone erastin (IKE) was obtained. Ike showed strong efficacy and selective lethality to BJ-derived tumor cells expressing oncogenic HRAS by inducing ferroptosis.^375^ Interestingly, the mechanism of action of a compound (MOA) is a set of target proteins and effector proteins necessary to produce its pharmacological effects in a specific cellular environment. MOA is of great significance in evaluating the efficacy and toxicity of compounds.^376^ A study found that altretamine is a new GPX4 inhibitor similar to sulfasalazine through the prediction-based MOA analysis of detecting mechanism of action by network dysregulation (demand) and combined with experiments, inhibiting the growth and development of diffuse large B-cell lymphoma (DLBCL) by inducing ferroptosis.^377^ FINO2, an endoperoxide containing 1,2-dioxane, induces ferroptosis in engineered cancer by playing a dual effect, directly oxidizing iron and indirectly inhibiting GPX4, showing a new mechanism of iron apoptosis inducer.^378^

#### Other targets

Excess iron can cause tissue damage and increase the risk of cancer. The most important mechanism of iron biotoxicity is the Fenton reaction of too much Fe^2+^ in cells, which leads to the accumulation of ROS and the production of a large number of hydroxyl radicals, resulting in the damage of cellular proteins, lipids, and DNA. Intervening in iron absorption and metabolism has become a method to treat tumors and other diseases. The application of iron-based nanoparticles can induce ferroptosis of tumor cells and inhibit tumor growth.^379^ In a word, tumor cells can increase the content of iron in cells by regulating iron metabolism, making cancer tissues more sensitive to ferroptosis. Studies have shown that intracellular iron overload can be prevented by knocking out the transferrin receptor (TFRC) on the cell surface, or the storage of iron in the inert pool can be increased by upregulating cytoplasmic ferritin to inhibit the occurrence of ferroptosis.^380^ Similarly, inhibiting the transcription factor iron-responsive element binding protein 2 (ireb2), which regulates iron metabolism, can reduce ferroptosis.^40^ In contrast, blocking intracellular iron output by knocking out solute carrier family 40 member 1 (slc40a1) will accelerate erastin-induced ferroptosis in neuroblastoma cells.^381^ In conclusion, the iron metabolism pathway and ferritin phagocytosis are the keys to regulating ferroptosis. In addition, mitochondrial lipids are also an important source of lipid peroxides during ferroptosis. Inhibition of mitochondrial tricarboxylic acid (TCA) cycle or functional mitochondrial electron transport chain (ETC) can reduce ferroptosis caused by cysteine deprivation.^382^ When cysteine is absent, cells will metabolize through glutamine decomposition, increase TCA in mitochondria, increase the production of lipid ROS, hyperpolarize mitochondrial membrane, and eventually collapse, thus inducing cell ferroptosis. In addition, mitochondrial fatty acid metabolism genes, including citrate synthase and recombinant acyl-coenzyme A synthetase long-chain family member 2 (acsf2), may be necessary genes for the occurrence of ferroptosis induced by erastin. In order to avoid the excessive accumulation of unfolded proteins in the endoplasmic reticulum, eukaryotic cells can activate a series of signal pathways to maintain endoplasmic reticulum homeostasis, which is called endoplasmic reticulum stress (ERs). Erastin can upregulate ERS response genes and induce the occurrence of ERS.^383^ Activating transcription factor 4 (ATF4) is the main signal transduction pathway of ERS involved in the activation of ferroptosis. It can increase cation transport regulators like protein 1 (CHAC1), promote the degradation of GSH, and induces the occurrence of ferroptosis. Puma, a regulator of apoptosis, is also downstream of ATF4 and upregulated in ERS induced by art, a ferroptosis inducer.^383^ There is evidence that ferrostatin-1 (fer-1) and liproxstatin-1 (lip-1) cannot reduce erastin-induced ERS by inhibiting lipid peroxidation.^383^

### Other RCD signaling pathways in cancer

#### Targeting parthanatos through PARP1/AIFM1 signaling pathways in cancer

**Fig. 7:**
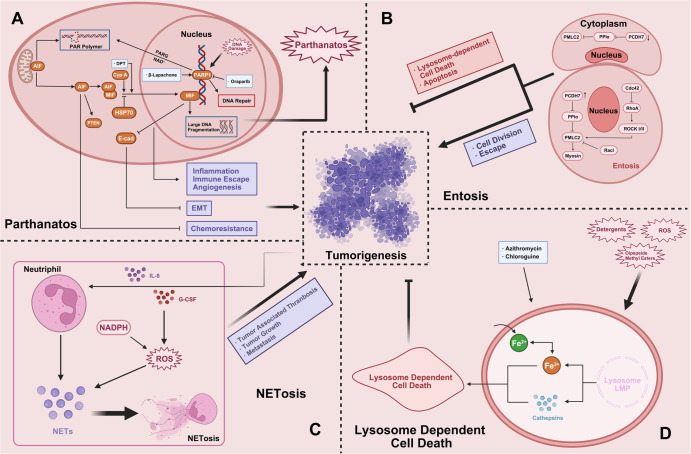
Small-molecule compounds targeting other pathways of RCD in cancer. Other main subroutines of RCD include parthanatos, entosis, NETosis and LCD. **A** When DNA is lost, PARP-1 is abnormally activated to produce a large amount of par. When the mitochondrial membrane is depolarized, the levels of ATP and NADPH decrease. AIF enters the nucleus from mitochondria, chromatin condenses and produces a large number of DNA fragments ranging from 15 KB to 50 KB, which induces the occurrence of parthanatos and promotes or inhibits tumorigenesis. **B** Entosis is an RCD form of "cannibalism" of cells. One cell engulfs and kills another cell, which is characterized by CIC structure. Cell adhesion and cytoskeleton rearrangement pathways (such as myosin, RhoA and ROCK) and other signaling molecules and regulatory factors (such as CDC42) play an important role in regulating the induction of entosis. It is worth noting that entosis can promote tumorigenesis through cell division and escape, or inhibit tumorigenesis through LCD and apoptosis. **C** The process of neutrophils secreting nets is called NETosis, which can promote tumor recurrence and metastasis. Overexpression of G-CSF and IL-8 in tumors can increase the number of neutrophils in blood, produce ROS and cause the formation of nets. In addition, NADPH oxidase can also directly produce ROS and promote the formation of nets. **D** When cells are exposed to lysosomal detergent, dipeptide methyl ester, lipid metabolites and ROS, lysosomes rupture and LCD is mediated by hydrolase or iron released by LMP, which inhibits the occurrence and development of tumors

Parthanatos is a new form of regulated cell death different from apoptosis. Parthanatos is closely related to the occurrence and development of many diseases such as tumors. Because the abnormal activation of PARP-1 is a prerequisite for inducing parthanatos, parthanatos is also known as PARP-1-mediated apoptosis. In addition, AIF and macrophage MIF are also key factors in the occurrence of parthanatos. In this process, PARP-1 acts as a DNA damage receptor, and its enzyme activity is rapidly activated after DNA damage,^384^ resulting in a sharp increase of PARP-1 activity by nearly 500 times. The generation of polymer par is dispersed in the cytoplasm or the target protein is parylated, and the dynamic balance between the generation and degradation of par in cells is broken, resulting in different lengths of par,^385^ which induces the release of AIF from mitochondria. In addition, AIF can bind to nuclease MIF and activate MIF. AIF and MIF then translocate to the nucleus, resulting in nuclear shrinkage, chromatin agglutination, and large DNA fragments ranging from 15 to 50 KB, resulting in parthanatos^386^ (Fig. 7A and Table 13).

**Table 13:** Compounds targeting other targets of regulated cell death (RCD) in cancer

Compound name and structure	Target	Mechanism in RCD	Cancer cell line (activity)	Indication of tumor type	Clinical trial identifier	Ref.
Olaparib	PARP ↓	Inhibit parthanatos		Breast cancer	NCT00516373 (phase I) NCT00777582 (phase I)	^391,392^
Niraparib	PARP ↓	Inhibit parthanatos		Breast cancer	NCT03329937 (phase I) NCT00749502 (phase I)	^393^
Rucaparib	PARP ↓	Inhibit parthanatos		Breast cancer, ovarian cancer	NCT00664781 (phase II) NCT02505048 (phase II)	
Veliparib	PARP ↓	Inhibit parthanatos		Breast cancer	NCT02210663 (phase I) NCT00892736 (phase I)	^394^
Talazoparib	PARP ↓	Inhibit parthanatos		Breast cancer	NCT01989546 (phase I/II) NCT01286987 (phase I/II)	
β-Lapachone	PARP ↑	Induce parthanatos	SK-Hep1 (4 μM)	Hepatocellular carcinoma		^396^
Deoxypodophyllotoxin (DPT)	ROS ↑, PARP ↑, nuclear translocation of AIF	Induce parthanatos	C6 (IC_50_ = 188 nM, 48h) SHG-44 (IC_50_ = 462 nM, 48h)	Glioma		^397^
Chloroquine	Caspase 9 ↑	Induce lysosome-dependent cell death	A549cisR (50 μM)	Non-small cell lung cancer		^412^
Azithromycin (AZM)	Permeability of lysosomal membrane ↑	Induce lysosome-dependent cell death	A549 (50 µM) CAL 27 (50 µM)	Lung cancer, human tongue squamous cell carcinoma		^413^
lansoprazole (LPZ)	Permeability of lysosomal membrane ↑	Induce lysosome-dependent cell death	A549 (100 µM) CAL 27 (100 µM)	Lung cancer, human tongue squamous cell carcinoma		^413^

↓ decrease/inhibition, ↑ increase/activation

Existing studies have shown that parthanatos is closely related to tumorigenesis and development.^387^ A study conducted microarray analysis on the expression of the PARP-1 gene in more than 8000 tumor samples.^388^ The results showed that the expression level of PARP-1 in breast cancer, ovarian cancer, endometrial cancer, lung cancer, skin cancer, and non-Hodgkin’s lymphoma was higher than that in the same amount of normal tissues, indicating that parthanatos was closely related to the above tumors. By constructing PARP-1 knockout mice, it was found that the risk of epithelial cancer in PARP-1 knockout mice was significantly reduced. The mechanism is that downregulating the level of PARP-1 protein can inhibit the activity of NF-κB and the expression of tumor-promoting related proteins regulated by NF-κB and inhibit the occurrence of parthanatos.^387^ In addition, PARP-1 knockout mice could significantly reduce the incidence of colorectal cancer induced by oxymethane (AOM) combined with dextran sulfate sodium (DSS). Mechanism study found that downregulating PARP-1 protein level can inhibit the occurrence of induced colorectal cancer by inhibiting the expression of cyclin D and transcription factor signal transducer and activator of transcription 3 (STAT3).^389^ The influence of parthanatos on tumorigenesis and development is mainly reflected in two aspects. On the one hand, in the process of rapid proliferation, radiotherapy, or chemotherapy, DNA is easy to be destroyed and leads to tumor cell apoptosis. One of the most important functions of PARP-1 is to participate in DNA repair, which is conducive to the survival of tumor cells. Therefore, the purpose of inducing tumor cell apoptosis can be achieved by inhibiting the activity of PARP-1. On the other hand, the occurrence of parthanatos mainly comes from the abnormal activation of PARP-1, so it can also lead to the occurrence of parthanatos in tumor cells by enhancing the activity of PARP-1 to inhibit the proliferation of tumor cells. Because PARP-1 is involved in many DNA repair pathways and the maintenance of genomic stability,^390^ the regulation of PARP-1 activity is an important means for the clinical treatment of related cancers.

Breast cancer proteins, especially BRCA1 and BRCA2, participate in homologous recombination repair (HRR). In clinical trials, PARP inhibitors are mainly concentrated in cancer patients with homologous recombination repair defects, including breast cancer and ovarian cancer patients with BRCA1 and BRCA2 mutations (gBRCA1/2m). At present, olaparib, niraparib, rucaparib, veliparib, and talazoparib inhibit the anticancer activity of parthanatos by inhibiting the catalytic activity of PARP-1 and PARP-2.^391–395^ β-Lapachone, a natural product obtained from the bark of the lapacho tree, induces parthanatos through the NQO1-dependent ROS-mediated RIPK1-PARP1-AIF pathway and promotes hepatocellular carcinoma cell death.^396^ The addition of a PARP-1-specific inhibitor blocked β-Lapachone-induced cell death.^396^ Deoxypodophyllotoxin (DPT), a natural active compound extracted from Anthriscus sylvestris, inhibits glioma growth by inducing excessive ROS, upregulating PARP-1 expression, and inducing nuclear translocation of AIF in xenograft glioma and glioma cells in vitro.^397^

#### Targeting Entosis through CDC42/RHOA/ROCK/Myosin signaling pathways in cancer

Entosis is an RCD form of "cannibalism" of cells.^293^ One cell engulfs and kills another cell, which is characterized by intracellular cell structure. After activation of entosis, similar cells are phagocytized and killed through LC3-related phagocytosis (LAP) and cathepsin B (CTSB) - mediated lysosomal degradation pathway. Cell adhesion and cytoskeleton rearrangement pathways (such as actin, myosin, RhoA, and rock) play an important role in controlling the induction of entosis. In addition to cell adhesion and cytoskeleton rearrangement pathways, other signaling molecules and regulatory factors (such as Cdc42) also participate in the regulation of entosis through different mechanisms (Fig. 7B and Table 13).

As early as more than 100 years ago, researchers observed an interesting cell sheath cell structure in human tumor tissue.^398^ At first, people speculated whether it was phagocytosis. Later, it was found that the internal cells forming this overlapping structure were living cells, and it was defined as cell in cell (CIC) structure.^399^ Due to the limitations of experimental conditions and instruments, the early research on CIC structure remained in the stage of phenomenon description. In recent years, scientists have made more in-depth research on the formation mechanism and biological function of CIC structure. Cell in cell structure refers to cell overlapping structure, which is mainly classified into the homotypic overlapping structure and heterotypic overlapping structure. CIC structure is a process in which one or more living cells exist in another cell to form a unique cell structure of cell sleeve cells and produce biological effects. It is common in tumor tissues and cells cultured in vitro. Usually, we call the cells inside the CIC structure effector cells and the cells outside the CIC structure target cells. It can be observed that the typical morphological feature of CIC structure is that after the effector cells enter the cytoplasm of the target cells, they are wrapped by the target cells, showing a "bird’s eye" shape, while the nucleus of the target cells is squeezed by the internal effector cells to form a "Crescent" shape.^400^ Entosis is a CIC structure formed between epithelial-derived cells (mainly tumor cells).^401^ Its mediated internal cell death is mainly lysosomal mediated caspase-3 (cysteine protease-3) - independent death.^401^ Some studies have found that it negatively regulates the formation of adhesion protein family molecule pcdh7 in entotic cell-in-cell structure and also found that the force ring plays a key role in the formation of entotic cell-in-cell structure.^402^ In mitotic surveillance, entosis selectively promotes aneuploid progeny cells to drill into adjacent cells to form cell-in-cell structure by activating the p53 signaling pathway and then is cleared to maintain the genomic stability of epithelial cells. This study also revealed the physiological function of entosis, found a new mechanism of abnormal cell clearance outside the regulation of the cell cycle, and revealed a new pathway for the p53 gene to maintain epithelial homeostasis and inhibit tumorigenesis at the cell level; On the one hand, it enriches the connotation of the existing mitotic surveillance mechanism; On the other hand, it expands the extension of entosis as a cell death mechanism involved in important biological processes of cancer.^402^

#### Targeting NETosis through NAPDH/ROS signaling pathways in cancer

NETosis is a form of RCD driven by NET, which is regulated by NADPH oxidase-mediated ROS production and histone citrullination.^293^ Neutrophil extracellular traps (NETs) are a form of inflammatory cell death found in 2004.^403^ They can trap bacteria, fungi, protozoa, and viruses. Nets are secreted by activated neutrophils and composed of DNA fibers, histones, and antibacterial proteins.^404^ They can fix pathogens and expose them to locally high lethal concentrations of effector proteins. The process of neutrophils secreting nets is called NETosis, which is the inflammatory cell death mode of neutrophils.^405^ NETosis involves a dynamic process of multiple signals and steps: the production of ROS, the nuclear migration of neutrophil elastase (NE), anti-myeloperoxidase antibody (MPO), histone modification, and chromatin degradation are the core mechanisms of the formation of nets.^406,407^ NETosis mediates histone citrullination, which eventually leads to chromatin deconcentration, nuclear membrane destruction, and chromatin fiber release (Fig. 7C and Table 13).

Nets are formed in the tumor microenvironment. The inducements of NETosis formation in malignant tumors include tumor cell colony-stimulating factor (G-CSF), and endothelial cell IL-8.^408^ Overexpression of G-CSF in tumors can increase the number of neutrophils in the blood, produce ROS and cause the formation of nets.^406^ Pancreatic cancer (PACA) cells can directly or indirectly induce the formation of nets.^409^ NETosis exists in animal models and tumor patients’ blood and tumor tissues. NETosis have both tumor-promoting and antitumor effects, which depend on the state of the immune system and the interaction of the tumor microenvironment.^405,407^ Studies have been devoted to the relationship between the formation of NETosis and tumorigenesis, progression, and metastasis and revealed the direct effect of NETosis on tumor cell proliferation through protease or activation signal.^410^ Tumor cells can induce the formation of NETosis in vivo and in vitro. Studies have shown that tumor cells can induce neutrophils to induce NETosis. Compared with the normal control group, neutrophils in the circulation of mouse models of chronic myeloid leukemia, breast cancer, and lung cancer were more likely to induce NETosis.^411^ The systemic effect of the tumor on the body leads to the increase of neutrophils activity forming NETosis.^411^ The close relationship between tumor cells and NETosis in the tumor microenvironment highlights the role of NETosis in tumor progression and metastasis. NETosis can awaken dormant tumor cells and promote tumor recurrence and metastasis. In addition, they can trap tumor cells in circulation and promote tumor proliferation and metastasis. The formation of NEtosis usually requires the activation of neutrophils and the production of ROS by NADPH oxidase. At present, it is still necessary to further study the possibility of NEtosis as a tumor treatment target and pharmacological interference with the formation of NEtosis. Although the relevant studies have achieved satisfactory results in the tumor model, the research on tumor patients is not satisfactory. It is necessary to focus further research on balancing the regulation and formation of NEtosis, taking NEtosis as a therapeutic target without affecting immune function.

#### Targeting lysosome-dependent cell death through LMP signaling pathways in cancer

Lysosome-dependent cell death (LCD), also known as lysosomal cell death, is a form of RCD mediated by hydrolase (cathepsin) or iron released by LMP, which is characterized by lysosomal rupture.^293^ When cells are exposed to lysosomal detergent, dipeptide methyl ester, lipid metabolites, and ROS, lysosomes rupture and then release a large number of hydrolases, leading to the occurrence of LCD. Cathepsin plays a major role in LCD. Blocking the expression or activity of cathepsin can reduce the occurrence of LCD. Lysosomal membrane permeabilization can also amplify cell death signal transduction in the case of apoptosis, autophagy-dependent cell death, and ferroptosis, which increases the complexity of the cell death pathway (Fig. 7D and Table 13).

At present, there are many mechanisms to explain lysosomal permeability. In view of the important role of lysosomal function in cancer cells, researchers have developed a variety of small molecular compounds for lysosomes, which can induce lysosomal membrane permeability or interfere with lysosomal function to kill tumor cells. For example, chloroquine can induce lysosomal membrane permeability to regulate lysosomal function, so as to restore the sensitivity of refractory non-small cell lung cancer cells to cisplatin;^412^ Azithromycin (AZM) can increase the expression of lysosomal galectin-3 spots, enhance the permeability of lysosomal membrane mediated by lansoprazole (LPZ), and significantly enhance the death of cancer cells induced by LPZ.^413^ These findings suggest that cancer cells that are not sensitive to traditional therapy may be effectively treated by using activated lysosomal cell death pathway. In addition, tumor cell lysosomes are more fragile than normal cells and are more prone to lysosomal membrane permeability and lysosomal-dependent cell death. Therefore, the intervention of lysosome-dependent cell death pathway may be an effective treatment strategy for many types of cancer. However, the current understanding of lysosome-dependent cell death is insufficient, and the related molecular targets and molecular mechanisms need to be further studied.

## Cancer treatment through multiple RCD signaling pathways

Simultaneous regulation of two or more RCD subroutines will be a promising treatment strategy for cancer. Lu01-m, a natural product with structural diversity and a variety of biological activities, induces cytotoxic activity in a variety of human prostate cancer cells through a variety of RCD mechanisms. Lu01-m mainly induces G2 / M phase cell cycle arrest and DNA damage, and finally induces apoptosis, necroptosis, autophagy, and other inhibition of tumor colony formation and tumor cell migration.^414^ Green tea extract Polyphenon E® blocks cell cycle G0/G1 checkpoint in PNT1a cells, activates caspases and cleaves poly(ADP ribose) polymerase 1 after autophagy, enabling cells to undergo anoikis.^415^ Polyphenon E® significantly enlarges the endoplasmic reticulum in PC3 cells, strongly upregulates GADD153/CHOP, and activates Puma, ultimately inducing necroptosis in prostate cancer cells.^415^ These new targets and strategies sensitize anti-apoptotic cells to other death pathways. Nobiletin extracted from citrus fruits can increase PARP levels in a dose-dependent manner, induce DNA damage and lead to apoptosis.^416^ It can also reduce mitochondrial membrane potential, induce ROS production and autophagy, induce GSDMD/ GSDME mediated pyroptosis, regulate a variety of regulatory cell death pathways, and significantly inhibit the proliferation of human ovarian cancer cells (Hocc).^416^ GSDME is highly expressed in human lung cancer. Both paclitaxel and cisplatin can significantly induce apoptosis of lung cancer cells, but cisplatin can induce more persistent caspase-3/GSDME-dependent pyroptosis than paclitaxel, suggesting that cisplatin may have additional advantages in the treatment of lung cancer with high expression of GSDME.^417^ In addition, cisplatin induces apoptosis and ferroptosis in A549 and HCT116 cells by causing reduced glutathione depletion and glutathione peroxidase inactivation, revealing a new potential mechanism of traditional chemotherapeutic drugs.^418^ A novel molecule, BAY 87-2243 (‘BAY’), induces mPTP opening and Δψ depolarization by inhibiting mitochondrial respiratory chain complex I (CI), promoting autophagosome formation, mitosis, and the associated increase in ROS, ultimately inducing ferroptosis, resulting in melanoma cell death.^419^ Knockout of autophagy-related gene 5 (ATG5) or addition of the ferroptosis inhibitor ferrostatin-1 inhibited BAY-stimulated autophagosome formation, increased cellular ROS, and tumor cell death.^419^ DHA, a semi-synthetic derivative of artemisinin, mediates cell cycle arrest in head and neck cancer cells through forkhead box protein M1 (FoxM1), reduces the expression of angiogenic factors, changes the angiogenesis phenotype of cancer cells, induces ferroptosis and apoptosis of cancer cells, and has efficient and specific antitumor activity.^420^ Heme oxygenase-1 (HO-1) has an antitumor function in cancer cells but has a cytoprotective function in normal cells.^421,422^ Piperlongumine (PL), a natural alkaloid isolated from pepper, inactivates kelch like ECH related protein-1 (Keap1) through mercaptan modification, then activates nuclear factor erythroid 2-related factor 2 (Nrf2), upregulates the expression of HO-1, induces apoptosis of breast cancer cells, promotes the production of ROS, and induces ferroptosis.^423^ However, it has no effect on normal human breast epithelial cells and finally forms a mechanism of selective killing of cancer cells^423^ (Table 14).

**Table 14:** Cancer treatment through multiple RCD signaling pathways

Compound name and structure	Target	Mechanism in RCD	Cancer cell line (activity)	Indication of tumor type	Ref.
Lu01-M	G2/M phase cell cycle arrest and DNA damage ↑	Induce apoptosis, necroptosis and autophagy	PC3 (IC_50_ = 1.03 ± 0.31 μg/mL) DU145 (IC_50_ = 2.12 ± 0.38 μg/mL) LNCaP (IC_50_ = 1.27 ± 0.25 μg/mL)	Prostate cancer	^414^
Polyphenon E®	Cell cycle G0/G1 checkpoint ↓, caspases and cleaves poly(ADP ribose) polymerase 1 ↑, GADD153/CHOP ↑	Induce anoikis and necroptosis	PC3 (IC_50_ = 145 μg/ml) PNT1a (IC_50_ = 35 μg/ml)	Prostate cancer	^415^
Nobiletin	PARP ↑, DNA damage ↑, ROS ↑, GSDMD/ GSDME ↑	Induce apoptosis, autophagy and pyroptosis	A2780 (IC_50_ = 35.31 μM) OVCAR3 (IC_50_ = 34.85 μM)	Ovarian cancer	^416^
Cisplatin	Caspase-3 / GSDME	Induce apoptosis and pyroptosis	A549 (IC_50_ = 25 μM, 48 h)	Lung cancer	^417^
Cisplatin	Glutathione depletion/ glutathione peroxidase ↓	Induce apoptosis and ferroptosis	A549 (IC_50_ = 10 μg/ml; 24 h) HCT116 (IC_50_ = 2–5 μg/ml; 48 h)	Lung cancer, colon cancer	^418^
BAY 87-2243 (‘BAY’)	CI ↓,ROS↑	Induce autophagy and ferroptosis	BG361 (IC_50_ = 4.8 ± 0.45 nM) SK-MEL-28 (IC_50_ = 2.4 ± 0.86 nM)	Melanoma	^419^
Dihydroartemisinin (DHA)	Cell cycle arrest, angiogenic factors ↓	Induce apoptosis and ferroptosis	HEP-2 (IC_50_ = 18.1 µM, 72h) CNE-1 (IC_50_ = 18.4 µM, 72h)	Head and neck cancer	^420^
Piperlongumine (PL)	Keap1 ↓, NRF2/HO-1 ↑, ROS ↑	Induce apoptosis and ferroptosis	MCF-7	Breast cancer	^423^

↓ decrease/inhibition, ↑ increase/activation

## Combination therapy of targeted small-molecule compounds in cancer

### Combination therapy for synthetic lethality in cancer

Synthetic lethality is a genetic interaction between two genes in which mutations in a pair of genes result in cell death, while the mutation of either gene is not lethal.^424^ Some DNA double-strand break repair (DSBR) genes have synthetic lethal relationships with oncogenes or tumor suppressor genes, and these genetic abnormalities could be targeted to kill cancer cells selectively.^425^ It can be a new direction of targeted tumor therapy to search for a target gene that has a synthetic killing effect with oncogenes and exert synthetic lethality. Therefore, combination therapy based on synthetic lethality is a viable and promising therapeutic strategy for cancer treatment.

PARP inhibitors are the first clinically approved cancer drugs designed to exploit synthetic lethality.^426^ At present, PARP inhibitors therapy has been successfully used to treat advanced breast and ovarian cancer patients harboring BRCA1/2 mutations and exhibiting homologous recombination (HR) deficiency.^427^ But some of these patients showed signs of resistance that limit the efficacy of PARP inhibitor monotherapy. Therefore, various research groups adopted a combination therapy strategy to induce cancer cells to produce synthetic lethal effects. In BRCA-mutant TNBC patients,^428^ there was increased expression of the MYC gene, which reduced the survival rate. The combination of MYC inhibitor, dinaciclib, and PARP inhibitor, niraparib, induced a potent synthetic lethal effect on TNBC cells with MYC overexpression.^429^ Sirtuin2 (SIRT2) is a kind of epigenetic regulator, and its disorder is the main factor in inducing cancer.^430^ SIRT2 inhibitor and sorafenib combination treatment exhibited potent ability to induce MCF-7 cell apoptosis, as well potential synthetic lethality effect.^431^ Aurora-A overexpression has been observed in breast cancer and is associated with poor prognosis. Aurora-A inhibitor, MLN8237 as a single agent, was ineffective in prolonging patient survival, so a strategy of combined inhibition of Aurora-A and Haspin may cooperatively regress the viability of breast cancer cells in vitro and in vivo.^432^ Moreover, IMMU-132, an antibody conjugate drug, and PARP inhibitors could significantly inhibit breast cancer cell growth and were well tolerated regardless of BRCA1/2 status. Currently, this combination strategy is being tested in phase II clinical trials in patients with advanced or metastatic solid tumors (NCT03992131).^433^ In addition, CHEK1 inhibitor, LY2603618, and Aurora-A inhibitor, alisertib, had a synthetic lethal effect. The combined treatment could trigger apoptosis of ovarian cancer cells and enhance the therapeutic effect of chemotherapy drugs.^434^

Studies have shown that thyroid carcinoma cells with BRAF V600E mutation were resistant to PLX4032, a BRAF inhibitor, and the dual blocking of EGFR and BRAF continuously inhibited the ERK/Akt pathway and led to an increased level of apoptosis as well induction of synthetic lethality.^435^ In BRAF-mutant and RAS/RAF wild-type CRC cells, double blocking of NEDD8 and EGFR pathways led to cell growth arrest and increased apoptosis induction.^436^ Sun et al. reported the novel synthetic lethal partners, Src and PARP1, that exerted a pronounced anticancer effect on HCC cells and could suppress the resistance to PARP1 inhibition.^437^ Moreover, a combination of sorafenib and selumetinib was also an effective therapy in HCC cells having high p-ERK levels. The efficacy and safety of the mixture in advanced HCC were demonstrated in phase I/II clinical trial (NCT01029418).^438^ In an FLT3(ITD)-positive AML cells, FLT3 inhibitor, AC220 combined with PARP1 inhibitor significantly inhibited proliferating leukemia stem cells and delayed disease onset.^439^ The combination of APG-2575 and ibrutinib could effectively inhibit the DLBCL cells with high expression of Bcl-2 and had a synergistic antitumor effect, which could improve the clinical treatment of lymphoma to a certain extent.^440^ Besides, a phase II clinical trial (NCT04494503) tested the safety, pharmacokinetic, pharmacodynamic, and efficacy of APG-2575 combined with ibrutinib in patients with relapsed/refractory CLL and small lymphocytic lymphoma (SLL). Additionally, Pan et al. had found that Bcl-2 inhibition and p53 activation could overcome apoptosis resistance and induce synthetic lethality in AML models.^441^ In phase II clinical trial, the safety and early efficacy of idasanutlin in combination with ABT-199 in young patients with neuroblastoma, AML, and ALL were evaluated (NCT04029688). There are many combined treatment strategies for synthetic lethality, and we have selected several representative examples for discussion and summary in Table 15.^442–444^

**Table 15:** Combination therapy for synthetic lethality in cancer

Compound 1	Compound 2	Coordination mechanism	Tumor type	Ref.
Dinaciclib (CDK inhibitor)	Niraparib (PARP inhibitor)	Induce apoptosis	Triple negative breast cancer	^429^
SIRT2 inhibitor I	Sorafenib (Multikinase inhibitor)	Induce apoptosis	Breast cancer	^431^
CHR-6494 (Haspin inhibitor)	MLN8237 (Aurora-A inhibitor)	Induce apoptosis	Breast cancer	^432^
Alisertib (AURKA inhibitor)	LY2603618 (CHEK1 inhibitor)	Induce apoptosis	Ovarian cancer	^434^
Gefitinib (EGFR inhibitor)	Vemurafenib (BRAF inhibitor)	Induce apoptosis	Thyroid carcinomas	^435^
Pevonedistat (NEDD8-activating enzyme inhibitor)	Cetuximab (EGFR inhibitor)	Induce apoptosis	Colorectal cancer	^436^
Dasatinib (Src inhibitor)	Olaparib (PARP inhibitor)	Induce apoptosis	Hepatocellular carcinoma	^437^
Selumetinib (ERK inhibitor)	Sorafenib (multikinase inhibitor)	Induce apoptosis	Hepatocellular carcinoma	^438^
AC220 (FLT3 inhibitor)	BMN673 (PARP1 inhibitor)	Induce apoptosis	Acute myeloid leukemia	^439^
APG-2575 (BCL-2 inhibitor)	Ibrutinib (BTK inhibitor)	Induce apoptosis	Diffuse large B-cell lymphoma	^440^
ABT-199 (Bcl-2 inhibitor)	Idasanutlin (MDM2 antagonist)	Induce apoptosis	Acute myeloid leukemia	^441^
PCI-34051/AR-42 (HDAC inhibitors)	KPT-9274 (NAMPT inhibitor)	Induce apoptosis	Acute myeloid leukemia	^442^
Panobinostat (HDAC inhibitor)	OTX015 (BRD inhibitor)	Induce apoptosis	Glioblastoma	^443^
Dasatinib (FGFR1 inhibitor)	Olaparib (PARP inhibitor)	Induce apoptosis	Pancreatic cancer	^444^

### Combination therapy for synergistic effects in cancer

Synergistic therapy has been widely applied in clinical treatment as a strategy to improve the efficacy of anticancer therapy. The appropriate drug combinations could acquire better curative effects with fewer adverse reactions and produce synergistic effects by acting on multiple pathogenesis of the disease.^445^ The combination of natural active compounds with therapeutic drugs should be a promising therapeutic strategy that can overcome various clinical drug deficiencies and improve the in vivo efficacy of drugs.^446^ This group combined piperine with celecoxib to synergistically exert an antiproliferation effect on colon cancer cells. Besides, the co-treatment could cause mitochondrial dysfunction and the activation of caspase to trigger apoptosis.^447^ Tetrandrine as a bisbenzylisoquinoline alkaloid extracted from *Stephania tetrandrine S*. Moore, when combined with protein kinase A inhibitor H89, the therapeutic efficacy enhanced notably.^448^ It was found that tetrandrine and H89 could synergistically inhibit tumor cell growth and induce apoptosis and autophagy by regulating ROS-induced PKA and ERK signaling pathways. In addition, it showed that c-Myc amplified cancer cells were more sensitive to tetrandrine/H89 combined therapy treatment.^448^ Additionally, co-treatment of curcumin analog PGV-1 and citrus flavonoid compound diosmin enhanced the cytotoxic effect on 4T1 cancer cells. Targeted regulation of cyclin-dependent kinase 1 (CDK1), KIF11, and AURKA proteins blocked the cell cycle and increased the number of mitotic catastrophes, resulting in senescence and death of 4T1 cancer cells.^449^

Chemotherapy is a principal treatment for cancer, but chemotherapy targets not only tumor cells but also healthy cells, leading to a variety of adverse effects.^450^ Therefore, combining chemotherapy drugs with other anticancer compounds could reduce side effects and synergistically increase the antitumor activity of drugs. In this work, combined treatment of curcumin and 5-FU could significantly increase the apoptosis rate of cancer cells, prolong the survival of immunodeficient mice, as well reduce the toxicity and adverse effects of 5-FU.^451^ Likewise, 5-FU had a synergistic effect with natural product withaferin-A in inhibiting proliferation. This combination could modulate endoplasmic reticulum (ER) stress, suppress the β-catenin pathway, induce cell arrest at the G2M phase, and thus induce CRC cells’ autophagy and apoptosis.^452^ Recently, flubendazole and 5-FU had been found to synergically inhibit cell proliferation and promote cell death by targeting p53 protein and activating ferroptosis.^453^ As we all know, dietary polyphenols could be used as a chemical sensitizer to enhance drug efficacy and reduce chemotherapy resistance. It was shown that the dietary flavonoid fisetin and paclitaxel possessed a synergistic effect on A549 NSCLC cells by activating mitotic catastrophe and promoting autophagic cell death of cancer cells which was indicated as a novel chemotherapy approach for NSCLC treatment.^454^ In addition, it was reported that arsenic trioxide (ATO) could induce apoptosis of breast cancer cells at high concentrations, but it was easy to cause side effects.^455^ Therefore, this group used the hTERT inhibitor BIBR1532, combined with ATO, to make cells sensitive at a low concentration of ATO, which could synergistically inhibit the survival, proliferation, and accelerated apoptosis of breast cancer cells.^455^

Targeted drug combination therapy is a prospective clinical therapeutic strategy that could induce apoptosis and autophagic cell death by regulating some targeted proteins and signaling pathways. WEE1 was overexpressed in some tumors, and inhibition or downregulation of WEE1 could lead to mitotic catastrophe. WEE1 inhibitors play a key role in the treatment of tumors.^456^ Combined inhibition of BET and WEE1 could synergistically attenuate the growth of NSCLC cells.^457^ Besides, the study had shown that BET inhibitor increased WEE1 inhibitor AZD1775-induced DNA double-strand breaks and cytotoxicity. The blockade of BET protein BRD4 would downregulate the nonhomologous end-joining (NHEJ) activity. When combined with the WEE1 inhibitor, it also diminished myelin transcription factor 1 (MYT1) expression, thereby promoting mitotic catastrophe.^457^ Moreover, PARP inhibitor rucaparib and PI3K inhibitor BKM120 had shown synergetic antitumor effects on GBM cell lines. BKM120 could reduce HR repair molecule expression, thus increasing the level of rucaparib-induced apoptosis, which could significantly improve the antitumor efficacy.^458^ A recent study found that the combination of CBL0137 and HDAC inhibitor panobinostat could synergistically suppress the growth of MYCN-amplified neuroblastoma cancer cells, accompanied by the induction of IFN response and the inhibition of DNA damage repair.^459^ Additionally, Ponatinib, a tyrosine kinase inhibitor (TKI), combined with asciminib could produce a synergistic apoptosis-induced effect in BCR-ABL1 mutant CML cell lines and murine Ba/F3 cells.^460^ AC220 (Quizartinib), an FLT3 receptor tyrosine kinase inhibitor, was used for AML treatment in clinical. In order to improve its anticancer effect, it is necessary to explore the potential synergistic effect of AC220 and other small molecules. A present study found that when treated in combination with the autophagy inhibitor TAK-165, AC220 could induce cancer cell death by activating chaperone-mediated autophagy.^461^ These results suggest that targeted autophagy should be regarded as an effective strategy to enhance the efficacy of cancer therapy. A study manifested that combined treatment with Fin56, a ferroptosis inducer, and Torin 2, an mTOR inhibitor, could synergistically inhibit the viability of bladder cancer cells, as well as induce ferroptosis and autophagy-dependent cell death through the glutathione peroxidase 4 (GPX4) protein degradation increased.^462^ Moreover, metformin combined with sulfasalazine, a ferroptosis inducer, had a synergistic effect on activating ferroptosis and repressing breast cancer cell proliferation^463^ (Table 16).

**Table 16:** Combination therapy for synergistic effects in cancer

Compound 1	Compound 2	Coordination mechanism	Tumor type	Ref.
Piperine	Celecoxib	Induce apoptosis	Colon cancer	^447^
Tetrandrine	H89 (Protein kinase A inhibitor)	Induce apoptosis and autophagy-dependent cell death	Breast cancer, hepatic carcinoma	^448^
Diosmin	PGV-1	Activate mitotic catastrophe	Triple negative breast cancer	^449^
Curcumin	5-fluorouracil (5-FU)	Induce apoptosis	Colorectal cancer	^451^
Withaferin-A	5-fluorouracil (5-FU)	Induce apoptosis and autophagy-dependent cell death	Colorectal cancer	^452^
Flubendazole	5-fluorouracil (5-FU)	Activate ferroptosis	Castration-resistant prostate cancer	^453^
Fisetin	Paclitaxel	Activate mitotic catastrophe and autophagy-dependent cell death	Non-small cell lung cancer	^454^
BIBR1532	Arsenic trioxide (ATO) As_2_O_3_	Induce apoptosis	Breast cancer	^455^
JQ1 (BET inhibitor)	AZD1775 (WEE1 inhibitor)	Activate mitotic catastrophe	Non-small cell lung cancer	^457^
Rucaparib (PARP inhibitor)	BKM120 (PI3K inhibitor)	Induce apoptosis	Glioblastoma	^458^
CBL0137	Panobinostat (HDAC inhibitor)	Induce apoptosis	Neuroblastoma	^459^
Ponatinib (Tyrosine kinase inhibitor)	Asciminib	Induce apoptosis	Chronic myeloid leukemia	^460^
AC220	TAK-165 (Autophagy inhibitor)	Inhibit autophagy-dependent cell death	Breast cancer	^461^
Fin56 (Ferroptosis inducer)	Torin 2 (mTOR inhibitor)	Induce ferroptosis and autophagy-dependent cell death	Bladder cancer	^462^
Metformin	Sulfasalazine (Ferroptosis inducer)	Activate ferroptosis	Breast cancer	^463^

### Combination therapy for reduced drug resistance in cancer

Primary and acquired drug resistance is one of the most important signs of cancer. Some new combination therapy strategies focus on the development of new targeted therapies. Chemotherapy, as a common tumor treatment strategy, leads to high mortality due to the frequent occurrence of drug resistance during treatment. Bcl-2 inhibitors have been shown to reverse chemoresistance and enhance sensitivity to other chemotherapeutic drugs in cancer.^464^ The combination of resveratrol (RSV) and anticancer drug docetaxel (DTX) stimulates cell apoptosis by inhibiting Bcl-2 and increasing Bax, induces necroptosis, reverses DTX resistance and synergistically enhances the anticancer effect of DTX.^465^ Acquired drug resistance is usually due to the reactivation of MEK-ERK1/2 pathway caused by NRAS mutation, increased BRAF copy number, and abnormal BRAF splicing.^466–468^ The efficacy of BRAFi and/or MEKI is associated with tumor T cell infiltration. The loss of CD8 + T cells and the inflow of tumor-associated macrophages are associated with acquired drug resistance to metastatic melanoma.^469^ The development of resistance to BRAFi+MEKi in metastatic melanoma remains a major clinical challenge. A study in BRAFi+MEKi-resistant melanoma by using etoposide to re-induce pyroptosis, inhibition of the ERK1/2 pathway, and cleavage of GSDME-induced pyroptosis produced a more potent antitumor immune response, overcoming treatment resistance.^470^ It is suggested that targeting this regulated cell death pathway represents a potential strategy for salvage treatment of patients with BRAFi + MEKI resistant melanoma. Cetuximab, an EGFR inhibitor, is commonly used as a targeted therapy for patients with breast cancer. Because long-term use is easy to leads to anti-EGFR treatment resistance, cetuximab resistance is the main clinical drug resistance problem in the treatment of EGFR overexpression cancer. The level of mir-155-3p is upregulated in breast cancer cells.^471^ GSDME is the direct binding target of mir-155-5p.^472^ Cetuximab combined with mir-155-5p antagomir can promote the pyroposis of EGFR overexpressed breast cancer cells, induce apoptosis, enhance the antitumor effect of cetuximab, and provide an alternative treatment for breast cancer patients by upregulating GSDME-N and cleaved caspase-1.^472^ Colorectal cancer (CRC) is the third most commonly diagnosed cancer in the world. Despite the continuous improvement of the treatment level of colorectal cancer, there are still a considerable number of advanced patients with postoperative metastasis or patients with metastasis at the initial diagnosis, and most of them involve the liver, lung, peritoneum, and distant lymph nodes.^314^ In addition to chemotherapy, there are different targeted therapies for metastatic colorectal cancer (mCRC). Anti EGFR antibodies (such as cetuximab or panimab) combined with chemotherapy is an effective treatment for patients with RAS wild-type MCRC only.^473^ Since downstream KRAS mutations exist in about 50% of CRC, the effectiveness of EGFR inhibitor combination therapy is usually limited by intrinsic drug resistance. The combination of β-elemene and cetuximab can induce ferroptosis and inhibit the migration of CRC cells mutated in KRAS by inducing ROS accumulation, consuming GSH, lipid peroxidation, upregulating HO-1 and transferrin, downregulating GPX4, SLC7A11, FTH1 and SLC40A1.^474^ The above effects were eliminated after the use of ferroptosis inhibitor.^474^ By inducing ferroptosis, it is expected to provide a prospective strategy for CRC patients with KRAS mutation. In addition, the combination of Andrographis and 5-FU, the active component from *Andrographis paniculata*, in xenograft animal models and CRC cell lines, inhibits CRC in a dose-dependent manner by activating ferroptosis and inhibiting β- catenin/Wnt signaling pathway, which is more effective than 5-FU or Andrographis alone.^475^ Apoptosis has been shown to be involved in the development of sorafenib resistance in HCC. Nuclear factor erythroid 2-related factor 2 (Nrf2) overexpression inhibits apoptosis and contributes to the chemical resistance of some cancers.^476^ However, Nrf2 plays a dual role in the treatment of cancer, depending on the type and stage of cancer. P62-keap1-nrf2 pathway plays an important role in protecting HCC cells from ferroptosis by upregulating ROS-related genes.^477^ Alkaloid trigonelline reverses drug resistance by inhibiting Nrf2 in vitro and tumor xenotransplantation model, thus increasing the anti HCC activity of erastin and sorafenib^477^ (Table 17).

**Table 17:** Combination therapy for reduced drug resistance in cancer

Compound 1	Compound 2	Coordination mechanism	Tumor type	Ref.
resveratrol (Nrf2 activator)	docetaxel (microtubule depolymerization inhibitor)	Induce apoptosis and necroptosis	Prostate carcinoma	^465^
Doxorubicin (topoisomerase-II inhibitor)	Etoposide (topoisomerase-II inhibitor)	Induce pyroptosis	Melanoma	^470^
Cetuximab (EGFR inhibitor)	miR-155-5p antagomir (5′-ACCCCUAUCACGAUUAGCAUUAA-3′)	Induce apoptosis and pyroptosis	Triple-negative breast cancer	^471^
β-elemene (apoptosis inducer)	Cetuximab antibody (EGFR inhibitor)	Induce ferroptosis	Colon cancer	^474^
Andrographis (NF-κB inhibitor)	5-fluorouracil (5-FU)	Induce ferroptosis	Colon cancer	^475^
Trigonelline (Nrf2 inhibitor)	Erastin (ferroptosis inducer)	Induce ferroptosis	Hepatocellular carcinoma	^477^
Trigonelline (Nrf2 inhibitor)	Sorafenib (Raf inhibitor)	Induce ferroptosis	Hepatocellular carcinoma	^477^

## Novel therapeutic strategies targeting RCD subroutines in cancer

At present, with the development of molecular biology, the molecular typing of most cancers has gradually become clear, and the small molecule targeted therapeutic drugs under its guidance is also undergoing phase I-III clinical research. However, the progress of targeted therapy is slow, and some cancers are not sensitive to endocrine therapy and molecular targeted therapy. However, the existing traditional chemotherapy has some problems, such as strong side effects and drug resistance. Therefore, optimizing the existing clinical treatment methods and looking for new and effective cancer treatment methods have become the current research hotspot.^478^

Some drugs can effectively induce cancer cells to produce RCD, but they can not be directly used in clinical cancer treatment because of their poor water solubility, nephrotoxicity, and other toxic side effects. Recently, studies have shown that exosomes and nanosystems play a great role in the treatment of cancer. In addition, nanoparticles have unique advantages in disease treatment because of their good hydrophilicity and targeting. Nanoparticles combined with drugs or physical methods targeting ferroptosis are a new and potential treatment strategy. Ferritin nanoparticles, known as erastin and rapamycin binding, are a nanodrug inspired by Abraxane. When ferritin nanoparticles are swallowed by cancer cells, the nanosystem can release erastin and rapamycin to inhibit systeam X_c_^−^ and autophagy, respectively, resulting in ferroptosis in tumor cells.^479^ In addition, the organic metal network (mon-p53) wrapped with p53 plasmid combined with iron can also induce the occurrence of ferroptosis by releasing iron and p53 plasmid to inhibit systeam X_c_^−^. In mice with cancer, mon-p53 treatment not only inhibited tumor growth but also prolong the life of mice.^480^ In addition, a novel cascaded copper-based metal-organic framework therapeutic nanocatalyst using HKUST-1 amplifies the treatment of hepatocellular carcinoma by integrating the cyclooxygenase-2 (COX-2) inhibitor meloxicam and the chemotherapeutic drug sorafenib.^481^ In recent years, a variety of regulatory cell death forms have been found and characterized by their corresponding molecular mechanisms. These mechanisms can stimulate autophagy-dependent cell death, apoptosis, necroptosis, pyroptosis, ferroptosis, and so on, providing a new choice for cancer treatment research. In order to effectively improve the intracellular iron level, promote the ferroptosis induction pathway, establish an effective ferroptosis/pyroptosis mediated system, and create an efficient ferroptosis/pyroptosis double induction nano delivery system TF-LipoMof@PL.^482^ This system is loaded with the metal organic skeleton (MOF) of the pH-sensitive lipid layer modified by transferrin.^482^ The iron in the cell is enriched by the iron-containing MOF. The modified transferrin on the lipid layer further promotes the endocytosis of MOF.^482^ The upregulated TfR expression on the surface of various tumor cells not only provides a natural advantage for more effective iron-mediated iron endocytosis, but also provides a target for therapeutic drug delivery.^483^ PL is loaded as a ferroptosis inducer in an iron-containing MOF coated with a dope pH-sensitive lipid layer decorated with transferrin. This system can induce Fenton reaction, provide H_2_O_2_ for the double induction system, increase ROS in tumor cells, cause ferroptosis and pyroptosis in cells, and achieve an effective anticancer effect.^482^ In recent years, a large number of studies have found that exosomes have the advantages of low immunogenicity, high biocompatibility, and high efficiency, and have good advantages in drug delivery. A research team constructed a folic acid (FA)-labeled Erastin-loaded exosome preparation (erastin@FA-exo) to target folate receptor-overexpressing TNBC cells. Compared with free Erastin, Erastin@FAexo increased the uptake rate of Erastin in MDA-MB-231 cells and induced ferroptosis by inhibiting Systeam X_c_^−^.^484^

## Concluding remarks and future perspectives

Of note, the dynamic balance between cell survival and death is not only a necessary condition to maintain the homeostasis of the organisms, but one of the important conditions for the growth and development. When excessive cell proliferation or normal cell death is inhibited, the incidence of malignant tumors increases greatly. Thus, the two significant characteristics of malignant tumors are uncontrolled cell proliferation and escaping programmed cell death.^485^ Tumor has many phenotypes, which are the basis of tumor cell growth and metastasis. Resistance to programmed cell death (PCD) is one of its important phenotypes.^486,487^ In the traditional sense, cell death is divided into regulatory and non-regulatory types. With the deepening of research, more and more regulatory cell death modes have been found and named. In this review, we summarize 9 subroutines of regulated cell death (RCD), including autophagy-dependent cell death, apoptosis, necroptosis, pyroptosis, ferroptosis, parthanatos, entosis, netotic cell death and lysosome-dependent cell death.^488^ Autophagy-dependent cell death played the Jannus role in the occurrence and development of tumors. In the early stage of tumor occurrence, autophagy-dependent cell death exerted a preventive effect in controlling or killing cancer cells, while in the formed tumor cells, autophagy-dependent cell death could maintain the survival of cancer cells and promote development. Apoptosis has been recognized as a key intracellular process that maintain organism homeostasis and promote survival. Inhibition or resistance of apoptosis often leads to the occurrence of tumors. In recent years, in order to reduce toxicity, reduce the risk of disease progression, promote individualized treatment of tumors and improve the prognosis of patients, researchers have done a lot of research in the direction of tumor treatment by targeting RCD. In this review, we summarize those 104 drugs treat tumors by mainly targeting apoptosis, 65 drugs treat tumors by mainly targeting autophagy dependent cell death, 12 drugs treat tumors by mainly targeting pyroptosis, 8 drugs treat tumors by mainly targeting ferroptosis, and 10 drugs treat tumors by mainly targeting other targets of regulated cell death (RCD). Long-term activation or inhibition of a single RCD subroutine often leads to drug resistance. We summarized 8 antitumor drugs that target multiple RCD subroutines and 36 combination therapies that target RCD.

Compared with ACD, RCD is controlled by specific signal transduction pathways and can be regulated by genetic signals or drug intervention. Loss of control over single or mixed types of regulatory cell death can lead to a variety of human diseases, including cancer. Different lethal subroutines in RCD will affect the progress of cancer and the response to treatment. In the early stage of the disease, cancer cells may have the characteristics of anti-cancer treatment because of the mutations that destroy the RCD pathway, and avoiding RCD is one of the important signs of cancer. Through the simultaneous regulation of multiple RCD signal pathways by a drug or gene, the drug resistance of cancer cells to a specific type of RCD can be avoided, so as to achieve the purpose of treatment. Further in-depth studies of the complexity of RCD in the body, especially in malignant tumors, is not only conducive to deepen a better understanding of intracellular signal molecules and the maintenance of homeostasis, but of great significance for the confirmation of therapeutic targets, the development of new small-molecule drugs and the intricate mechanisms of drug resistance. Based on the current research results, focusing on the crosstalk between different RCD pathways may be a new direction of cancer treatment research in the future.
